# Copper(ii)-containing tungstotellurates(vi): syntheses, structures and their catalytic performances in selective oxidation of thioethers†

**DOI:** 10.1039/d0ra02609c

**Published:** 2020-06-12

**Authors:** Xuanyao Li, Peihe Li, Jinghai Liu, Zhengguo Lin, Changwen Hu

**Affiliations:** Key Laboratory of Cluster Science of Ministry of Education, Beijing Key Laboratory of Photoelectronic/Electrophotonic, School of Chemistry and Chemical Engineering, Beijing Institute of Technology Beijing 100081 China linzhengguo11@163.com cwhu@bit.edu.cn; Inner Mongolia Key Laboratory of Carbon Nanomaterials, Nano Innovation Institute (NII), College of Chemistry and Materials Science, Inner Mongolia University for Nationalities Tongliao 028000 China lipeihe1988@aliyun.com

## Abstract

We report the syntheses and structures of two new copper(ii)-containing tungstotellurates(vi) Na_12_[Te^VI^_2_W_8_O_38_Cu_2_(H_2_O)_2_]·7H_2_O (Te_2_W_8_Cu_2_) and Na_6_[Te^VI^W_6_O_24_Cu(NH_2_CH_2_CO_2_)_2_]·6H_2_O (TeW_6_Cu). The two compounds were synthesized by a simple one-pot method and characterized by single-crystal X-ray diffraction (XRD), powder XRD, FT-IR spectroscopy, elemental analysis, and thermogravimetric analysis in the solid state. Furthermore, their catalytic properties for the selective oxidation of thioethers were also studied systematically. The catalytic experiment results indicate that the tungstotellurate(vi) Te_2_W_8_Cu_2_ is an effective heterogeneous catalyst for the selective oxidation of thioethers to sulfoxides or sulfones by an H_2_O_2_ oxidant at room temperature. Under the ambient conditions, Te_2_W_8_Cu_2_ can convert 99% of methyl(phenyl)sulfane to sulfoxides or sulfones with 96% or 99% selectivity, respectively, and the utilization rate of H_2_O_2_ is up to 80%. Furthermore, Te_2_W_8_Cu_2_ as a heterogeneous catalyst is stable in the reaction and could be reused at least five cycles with conserved activity.

## Abstract 2

Two copper(ii)-containing tungstotellurates(vi) were synthesized and present very high catalytic activity for the selective oxidation of thioethers under ambient conditions.

## Graphical abstract

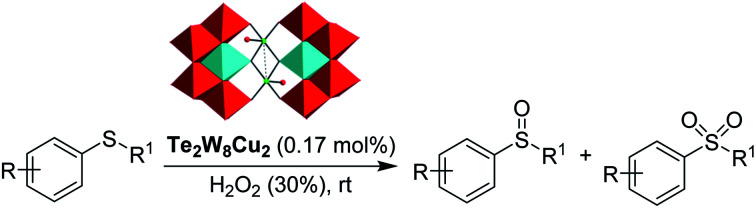

## Introduction

The selective oxidation of thioethers into the corresponding sulfoxides is a very important process in organic synthesis chemistry, because the sulfoxides are a type of essential building block in pharmaceuticals, agrochemicals, and other fine chemicals.^1^ Many significant methods have been developed for the synthesis of sulfoxides.^2,3^ Traditionally, the process was carried out with a wide variety of oxidant agents, such as peracids, dioxiranes, NaIO_4_, MnO_2_, CrO_3_, SeO_2_, and PhIO.^4^ However, these oxidation reactions relied upon strong or environmentally-unfriendly oxidants, some of which are hazardous or toxic. Therefore, developing an environmentally benign method and using easily available oxidants in thioethers oxidation is attractive and needs to be further explored. H_2_O_2_ can be used as oxidant in the oxidation of thioethers because it's a green oxidant and water is the only by-product.^5^ However, how to improve the utilization ratio and reduce the consumption of H_2_O_2_ with good catalytic selectivity in the oxidation reaction of thioethers still remains a challenge. In recent years, some polyoxometalates (POMs) were used as effective catalysts for the oxidation of thioethers using H_2_O_2_ as oxidant.^6–8^ In those reported procedures, low conversion of thioethers and the service life and reusability of the POM catalysts also need to be further improved. Consequently, based on the above reported results and our previous work on the oxidation of sulphides,^9^ We believe that more attractive catalytic systems can be developed along this line to further increase the utility of these thioethers oxidation reactions by using some new stable and recyclable POM catalysts.

As we all know, POMs are a large class of discrete anionic metal–oxygen clusters of early transition metals with high oxidation states, such as Mo, W, V, Nb, Ta,^10–13^ which present abundant structural diversity, favourable electrical carrier properties and great potentials in medicine, catalysis and material science.^14^ The most significant subset of POMs is the heteropolyoxometalates that incorporate various anion templates as heterogroups, such as pyramidal groups ({Sb^IV^O_3_}, {Bi^III^O_3_}, {Se^IV^O_3_}), tetrahedral groups ({P^V^O_4_}, {Si^IV^O_4_}, {Ge^IV^O_4_}, {As^V^O_4_}), and octahedral groups ({I^VI^O_6_}, {Cr^VI^O_6_}, {Te^VI^O_6_}) *et al.*^15–17^ It is well-known that the heterogroups play a key role in the structures and properties of these POMs, especially the redox properties, which is very important to their redox catalytic activities.^18^ Since heteropolytungstates are usually synthesized in acidic media by the condensation of molybdate or tungstate with relevant heteroanions,^19^ great achievements have been made for Keggin and Dawson type POMs by incorporating different tetrahedral heterogroups into their anionic cluster structures.^20^ It is worth mentioning that the POMs with octahedral hererogroups usually present interesting redox properties and very few POMs with octahedral heterogroups were reported, especially with the [Te^VI^O_6_]^6−^ heterogroup.^21^ To the best of our knowledge, many Anderson–Evans type [TeW_6_O_24_]^6−^ POM derivatives were reported such as {[Ni(H_2_O)_3_]_2_[TeW_6_O_24_]},^21^ {[Ca(H_2_O)_4_]_2_(TeW_6_O_24_)}^22^ and [4-H_2_-methyl-imz]_6_[TeW_6_O_24_]·Te(OH)_6_.^23^ In 2010, Ali and coauthors employed Anderson–Evans type POM {[Cu(en)_2_]_3_[TeW_6_O_24_]} as catalyst for the epoxidation of cyclohexene and styrene showed good catalytic efficiency.^24^ Recently, we reported the self-assembly of a series Ln(iii)-containing tungstotellurates(vi) with interesting photoluminescence properties.^25^

In order to develop new stable and recyclable POM catalysts for selective oxidation of thioethers, in this work, we prepared two new copper(ii)-containing tungstotellurates(vi) by a simple one-pot synthetic method. The two tungstotellurates(vi) 
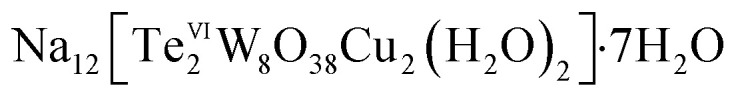
 (Te_2_W_8_Cu_2_) and Na_6_[Te^VI^W_6_O_24_Cu(NH_2_CH_2_CO_2_)_2_]·6H_2_O (TeW_6_Cu) were fully characterized in solid state and their heterogeneous catalytic performances in selective oxidation of thioethers were also studied systematically in the presence of 30% H_2_O_2_.

## Experimental

### Materials and methods

Na_6_TeW_6_O_24_·22H_2_O (TeW_6_) was prepared according to the published procedure and characterized by IR spectroscopy.^26^ All reagents were purchased from commercial sources, and used without further purification. Elemental analyses were determined by inductively coupled plasma mass spectrometry (ICP-MS) with PerkinElmer NexlON 350X spectrometer and the Elemental Analyser (C, H, N). FT-IR spectra (KBr pellets) were recorded with a Nicolet 170SX-FT/IR spectrometer. Thermogravimetric analyses were carried out with a TG-DTA 6200 device at a heating rate of 10 °C min^−1^ under nitrogen atmosphere. Powder X-ray diffraction (PXRD) data were recorded on a Bruker D8 instrument equipped with graphite-monochromatized Cu Kα radiation (*λ* = 0.154060 nm; scan speed = 8° min^−1^; 2*θ* = 5–50°) at room temperature.

### Synthetic procedures

#### Synthesis of 
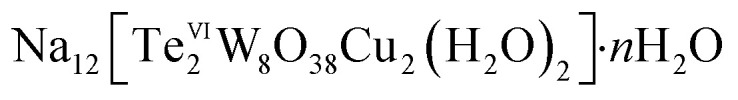
 (*n* = 7–38)

A mixture of Na_2_WO_4_·2H_2_O (1.5 g, 4.55 mmol), Te(OH)_6_ (0.1 g, 0.44 mmol), dimethylamine hydrochloride (1.5 g, 0.26 mmol), glycine (0.025 g, 0.05 mmol), Cu(OAc)_2_·H_2_O (0.075 g, 0.02 mmol) were dissolved in water (20 ml). The suspended solution was stirred for 2 hours. After filtration, the blue solution was kept in open air for slow evaporation, pH value of the solution is 8.9. The green crystals obtained after 1 week at room temperature (yield: 47% based on Te). Selected IR (2% KBr pellet, *ν*/cm^−1^): 1631(m), 940(m), 884(s), 871(m), 689(m), 615(w), elemental analysis (%) for Na_12_[Te_2_W_8_O_38_Cu_2_(H_2_O)_2_]·7H_2_O: calcd Na 9.52, W 50.73, Te 8.80, Cu 4.38; found Na 8.80, W 51.50, Te 8.89, Cu 4.70.

#### Synthesis of Na_6_[Te^VI^W_6_O_24_Cu(NH_2_CH_2_CO_2_)_2_]·*n*H_2_O (*n* = 6–20)

The synthetic procedure is exactly extending process of that of Te_2_W_8_Cu_2_. After removal of the green crystals of Te_2_W_8_Cu_2_ following the above procedure, led to blue crystals of TeW_6_Cu within 2–3 days (yield: 21% based on Te). Selected IR (2% KBr pellet, *ν*/cm^−1^): 1647(m), 1604(m), 1436(s), 1389(s), 1373(m), 1210(m), 1165(m), 1067(m), 959(m), 928(m), 903(w), 895(w), 699(m), 637(m), 551(m), 460(m), elemental analysis (%) for Na_6_[TeW_6_O_24_Cu(NH_2_CH_2_CO_2_)_2_]·6H_2_O: calcd. C 2.32, N 1.352, Na 6.66, W 53.2, Te 6.16, Cu 3.07; found C 2.32, N 1.608, Na 7.15, W 53.1, Te 6.38, Cu 3.07.

### X-ray crystallography

Crystal data for all compounds were collected at 150 K on a Bruker APEX 2 DUO CCD single-crystal diffractometer equipped with a sealed Mo tube and a graphite monochromator (*λ* = 0.71073 Å). The selected crystals were selected by examination under mineral oil using a polarising microscope and mounted in a Hampton cryoloop with oil and placed within one minute under a stream of cold N_2_. The structure solution and refinement were carried out by the *SHELXTL* program package (Bruker), and all structures were solved by direct methods and refined by the full-matrix least-squares method (Σ*w*(|*F*_o_|^2^ − |*F*_c_|^2^)^2^).^27^ The hydrogen atoms of waters were not incorporated in the refinements, and all other atoms were refined with anisotropic thermal parameters. The crystal data and structure refinement details for the two compounds are discussed and summarized in Table S1.† The crystallographic data have been deposited to the Cambridge Crystallographic Data Centre (CCDC) as entries CCDC-1991106, CCDC-1991107.

### Catalytic oxidation of thioethers

The selective oxidation of thioethers using different catalysts were carried out as follows. Thioethers (0.5 mmol) and 30% H_2_O_2_ (0.6 mmol or 1.25 mmol) were dissolved in EtOH/MeCN (1 ml) in a pressure tube (10 ml), then added catalyst (0.17 mol%) with a sealing cap at room atmosphere. The reaction mixture was vigorously stirred until the completion as indicated by GC. The reaction mixture was filtered through a short pad of celite and quenched with water. Then the mixture was extracted with dichloromethane (10 ml × 3), dried over MgSO_4_, and evaporated under reduced pressure to afford the crude product, which was further purified by flash chromatography on silica gel with *n*-hexane/EtOAc to obtain the corresponding sulfoxides or sulfones. In the recycle experiments, the catalyst was filtered after the reaction and washed with dichloromethane and dried under vacuum at 50 °C and used for the next cycle.

## Results and discussion

### Synthesis and structure description

As described in the synthetic procedures above, the two copper(ii)-containing tungstotellurates(vi) Te_2_W_8_Cu_2_ and TeW_6_Cu were prepared by simple one-pot reaction with a time-resolved process in which the hydrated salts of the two compounds were isolated successively from the same mother liquor as bulk-pure materials. The crystals of Te_2_W_8_Cu_2_ were isolated firstly and then the crystals of TeW_6_Cu. We noticed that the glycine ligand plays a very important role in the synthesis of the two compounds although it is absent in the final structure of Te_2_W_8_Cu_2_. Moreover, heteropolytungstates are usually formed in condensation reactions of tungstate oxoanions with relevant heteroanions in an acid medium, but the two copper(ii)-containing tungstotellurates(vi) were obtained straightly in the reaction solution without addition of appropriate acid and the pH value is 8.9, which means the two compounds can be constructed in the presence of Te(OH)_6_ which can provide enough protons during the condensation reactions.

**Fig. 1:**
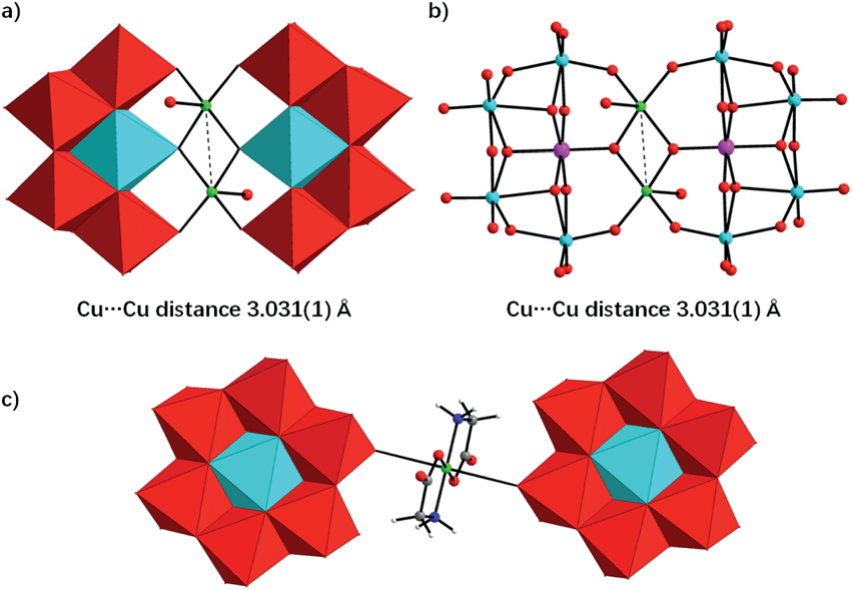
Polyhedral/ball-and-stick representation of the core structures of Te_2_W_8_Cu_2_ (a and b) and TeW_6_Cu (c). Color code: WO_6_, red octahedron; TeO_6_, turquoise octahedron, Cu, green balls, O, red balls, C, light gray balls, N, blue balls, H, white balls.

Single-crystal X-ray diffraction analysis reveals that Te_2_W_8_Cu_2_ is a di-copper-containing tungstotellurate(vi) which crystallizes in the triclinic lattice with space group *P*1̄. As shown in Fig. 1a, the polyanion cluster 
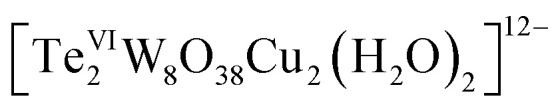
 comprises two {TeW_4_O_19_} subunits linked *via* two five-coordinate Cu^II^ ions in an anti-conformation. The {TeW_4_O_19_} subunit is a divacant fragment of the well-known Anderson–Evans structure {TeW_6_O_24_}, in which four edge-sharing WO_6_ octahedron formed a slightly distorted di-vacant hexagon and the central octahedral cavity is occupied by a {Te^VI^O_6_} heterogroup. The Te–O bond length are in the range of 1.881(4) Å to 1.968(4) Å, and the average distance is 1.928(4) Å. The two {TeW_4_O_19_} subunits are further connected by two Cu^II^ ions *via* multiple W–O–Cu and Te–O–Cu bonds. Each Cu^II^ center adopts a distorted square-pyramidal coordination sphere which tetra-coordinated by four terminal O atoms in the square plane from two {TeW_4_O_19_} subunits, and the Cu–O bond length is between 2.092(2) to 2.108(2) Å, which is comparable to the Cu–O bond lengths reported. The last coordinate site of Cu^II^ center is occupied by one terminal H_2_O at the conic node position of the square-pyramidal. The two Cu^II^ ions are not within the same planar of {TeW_4_O_19_} subunits and connected *via* two μ_3_-O atoms from the {TeO_6_} heterogroups, the distance between the two Cu^II^ ions is 3.031(1) Å and the angle of Te–O–Cu is averaging 126.2°, the other bond lengths and angles are summarized in Table S2.† The individual 
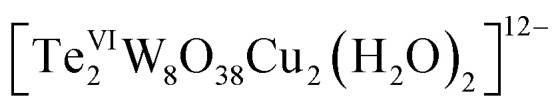
 polyanions can be further linked by sodium ions to form a 3D structure in solid state, as shown in Fig. S1a.†

For compound TeW_6_Cu, X-ray diffraction analysis reveals that it consists of one dimensional organic–inorganic hybrid polymeric chains having zig–zag architecture, which crystallizes in the triclinic lattice with space group *P*1̄ in form of sodium salts. As shown in Fig. 1b, the polymeric chain is formed by Anderson–Evans type [Te^VI^W_6_O_24_]^6−^ polyoxoanion and {Cu(gly)_2_} complex *via* the multiple O–Cu–O bonds. The polyoxoanion [Te^VI^W_6_O_24_]^6−^ in TeW_6_Cu is similar to the structures reported for other Anderson–Evans type anions with *D*_3d_ symmetry, in which a {TeO_6_} octahedron is surrounded by six edge-sharing {WO_6_} octahedra. The Te–O bond distances and O–Te–O bond angles are exhibited in Table S2,† and it can conclude that there is only mild distortion of the {Te^VI^O_6_} octahedra. The Cu^II^ centers in the chain present a distorted octahedral coordination sphere which is tetra-coordinated by two O atoms and two N atoms positioned in the equatorial plane from the carboxylate and amine groups of two glycine ligands, and the other two coordinate sites at the apical position are occupied by two O atoms from the neighbouring [Te^VI^W_6_O_24_]^6−^ polyoxoanions. The individual polymeric chains can be further linked by sodium ions to form a 3D structure in solid state, as shown in Fig. S1b.†

### Catalysis

Selective oxidation of thioethers to the versatile utility of sulfoxides and sulfones is considered to be a topical interest in organic synthesis. As mentioned above, POMs can be used as effective catalysts for the oxidation of thioethers using H_2_O_2_ as oxidant. In this work, in order to further develop a more attractive catalytic system for the selective oxidation of thioethers, we selected three different tungstotellurates(vi), the classic Anderson–Evans type POM TeW_6_, the organic–inorganic hybrid complex TeW_6_Cu, and the di-copper-containing POM Te_2_W_8_Cu_2_, as heterogeneous catalysts for this reaction and optimized the reaction conditions systematically. Moreover, we also verified that the Te_2_W_8_Cu_2_ is an effective heterogeneous catalyst with good conversion and selectivity for the oxidation of different thioethers to corresponding sulfoxides or sulfones at room temperature.

In order to investigate the catalytic performance and effects of the different catalysts for selective oxidation of thioethers, Te_2_W_8_Cu_2_, TeW_6_Cu, TeW_6_, CuO and Cu(OAc)_2_ were used as catalysts, and the catalytic reaction experiments of the oxidation of thioethers were investigated systematically by using H_2_O_2_ as oxidant under different conditions. At the beginning of our studies, in order to find out the best catalyst and optimize the reaction conditions for this catalytic system, methyl phenyl sulfide (1a) was employed as the model substrate for the oxidation reaction. The catalytic study results were summarized in Table 1. Notably, when the reaction was carried out in the absence of catalysts, only trace of product methyl phenyl sulfoxide (2a) was observed, and no methyl phenyl sulfone (3a) was observed, and the intact substrate 1a can be recovered (Table 1, entry 1). The copper(ii) salts and oxides, such as Cu(OAc)_2_ and CuO were first evaluated in the reaction (1a to 2a), although the selectivity of 2a was 99%, while the conversion of 1a were 19% and 31%, respectively. Considering the Anderson–Evans type TeW_6_ is the basic structure of TeW_6_Cu, we also tested the activity of TeW_6_ in the model reaction (1a to 2a). The results show that the conversion of 1a was 88% and the selectivity of 2a was 84%. Continuing experiments, the activity of TeW_6_Cu and Te_2_W_8_Cu_2_ were also examined in the model reaction, as shown in Table 1. Interestingly, according to the results of the catalysts screening, the final conversion of 1a and selectivity of 2a and 3a indicate that Te_2_W_8_Cu_2_ is the best catalyst for the both selective oxidation reactions of 1a to 2a and 1a to 3a (Table 1, entries 1–7) under ambient conditions. The selectivity of 2a is up to 96% when using MeOH or EtOH as solvent (Table 1, entry 3). Compared to 1.0 equiv. H_2_O_2_ (30%), the 1.2 equiv. H_2_O_2_ (30%) showed almost equal quality conversion rate (99%) of 1a (Table 1, entries 3–4). Furthermore, the selectivity of 3a is up to 99% when using MeCN as solvent and H_2_O_2_ (30%) (2.5 equiv.) as oxidant (Table 1, entry 6), which is much better than using H_2_O_2_ (30%) (2.0 equiv.) as oxidant. Based on the results summarized in Table 1, the optimized reaction conditions for this catalytic system are as follow: (1a to 2a): 0.17 mol% Te_2_W_8_Cu_2_, 1.2 equiv. of H_2_O_2_ (30%) in 1.0 ml of MeOH or EtOH at room temperature for 8 h; (1a to 3a) 0.17 mol% Te_2_W_8_Cu_2_, 2.5 equiv. of H_2_O_2_ (30%) in 1.0 ml of MeCN at room temperature for 6 h.

**Table 1:** Optimization of reaction conditions^a^

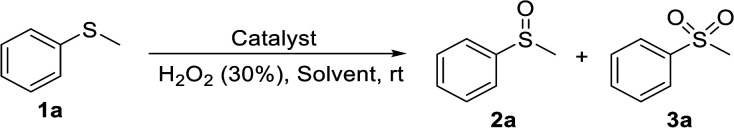					
Entry	Catalyst	Conv.^b^ (%)	2a Sel.^b^ (%)	3a Sel.^b^ (%)	UR (%) (H_2_O_2_)
1^c^	None	3	99	0	2.5
2^c^	TeW_6_Cu	89	86	14	64
3^c^	Te_2_W_8_Cu_2_	99	96	4	80
4^d^	Te_2_W_8_Cu_2_	91	96	4	73
5^e^	TeW_6_Cu	99	7	93	74
6^e^	Te_2_W_8_Cu_2_	99	1	99	79
7^f^	Te_2_W_8_Cu_2_	99	21	79	79

a Reaction conditions: 1a (0.5 mmol), catalyst (0.17 mol%), solvent (1 ml).

b Yields determined by GC analysis using 1-adamantanol as an internal standard.

c H_2_O_2_ (30%) (1.2 equiv.), MeOH or EtOH (1 ml), 8 h.

d H_2_O_2_ (30%) (1.0 equiv.), MeOH or EtOH (1 ml), 8 h.

e H_2_O_2_ (30%) (2.5 equiv.), MeCN (1 ml), 6 h.

f H_2_O_2_ (30%) (2.0 equiv.), MeCN (1 ml), 8 h. UR = utilization rate.

**Fig. 2:**
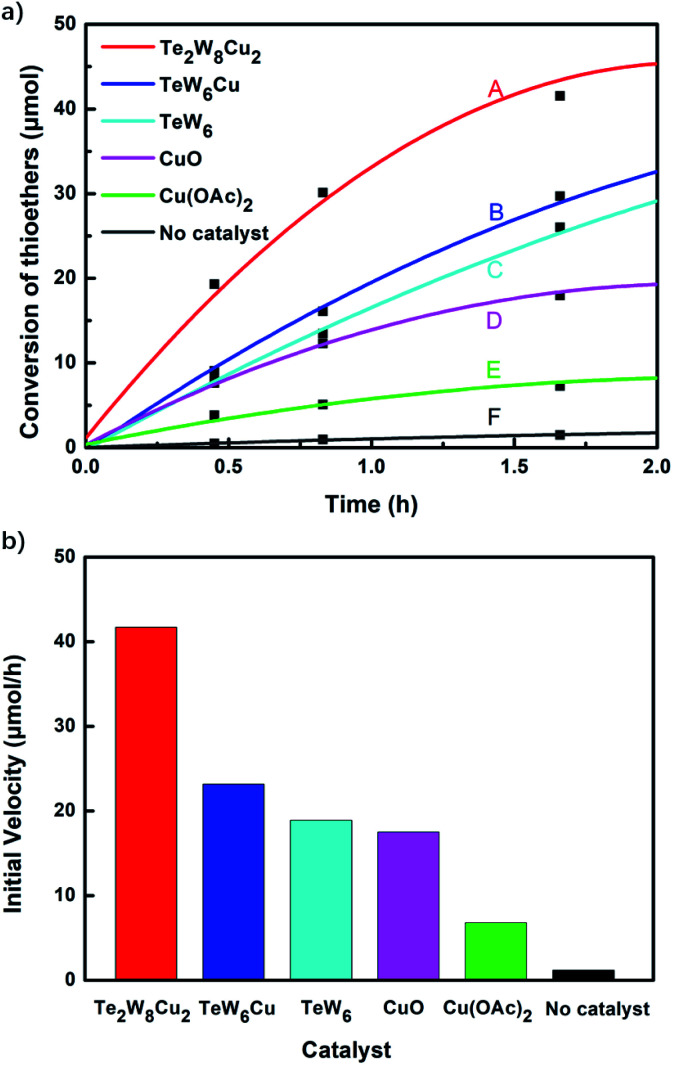
The conversion of thioanisole 1a with reaction time in the oxidation of thioanisole reaction by the catalyst of Te_2_W_8_Cu_2_, TeW_6_Cu, TeW_6_, CuO and Cu(OAc)_2_ (a). The initial velocity of the reaction by using different catalysts (b). (Reaction conditions: thioanisole 1a (0.5 mmol), catalyst (0.17 mol%), 30% H_2_O_2_ (1.0 equiv.), EtOH (1 ml), rt. All catalyst has the same number of moles of Cu, except TeW_6_. 1-adamantanol as an internal standard).

**Fig. 3:**
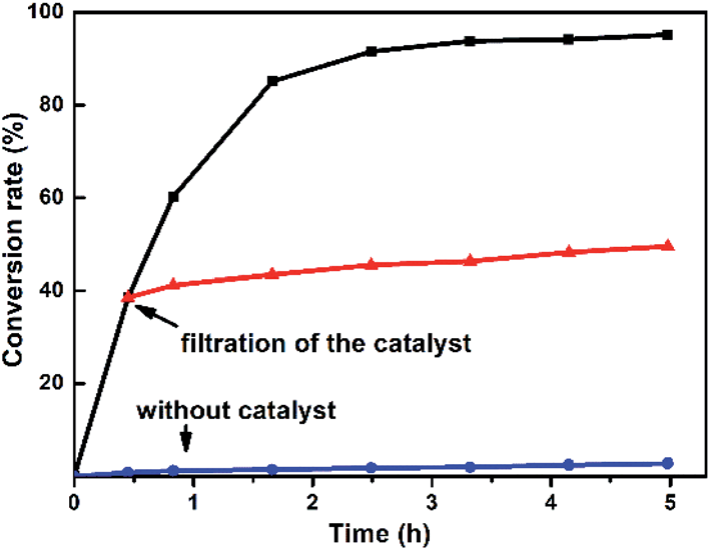
Leaching test for the oxidation of thioanisole 1a to 2a by using Te_2_W_8_Cu_2_ as catalyst. (Reaction conditions: 1a (0.5 mmol) Te_2_W_8_Cu_2_ (0.17 mol%), 30% H_2_O_2_ (1.2 equiv.), EtOH (1.0 ml), rt).

The variation of different catalysts Te_2_W_8_Cu_2_, TeW_6_Cu, TeW_6_, CuO and Cu(OAc)_2_ with reaction time in the oxidation reaction is demonstrated in Fig. 2a, and their kinetic studies indicated that if the reaction was carried out using Te_2_W_8_Cu_2_ as catalyst, the reaction proceeded much faster than using other catalysts, which means the Te_2_W_8_Cu_2_ present best activity for this reaction. The initial velocity order of the different catalysts is Te_2_W_8_Cu_2_ > TeW_6_Cu > TeW_6_ > CuO > Cu(OAc)_2_ > no catalyst (41.70 > 23.18 > 18.88 > 17.50 > 6.79 > 1.17, conversion moles per unit time). There are two possible reasons may explain these results. Firstly, many other POMs were used as effective catalyst for the oxidation of thioethers due to their excellent redox properties, and the tungstotellurates(vi) Te_2_W_8_Cu_2_, TeW_6_Cu, TeW_6_ with {Te^VI^O_6_} heterogroup can also present interesting oxidation catalytic activity which is much better than the simple cupric salts and cupric oxides for the selective oxidation of thioethers. Sencondly, by introducing the Cu^II^ ions into the structures of the tungstotellurates(vi) Te_2_W_8_Cu_2_ and TeW_6_Cu, the Cu^II^-containing tungstotellurates(vi) are more active than the simple Anderson–Evans type tungstotellurates(vi) TeW_6_, because the Cu^II^ centers can affect their redox properties and offer the new active sites for the catalysts during the catalytic reaction. More importantly, the structure and the coordination environment of Cu^II^ in Te_2_W_8_Cu_2_ and TeW_6_Cu are different, which can also affect their catalytic activity for selective oxidation of thioethers. As proved by single crystal analyses, for Te_2_W_8_Cu_2_, there are two Cu^II^ centers in the structure, and each Cu^II^ center is five-coordinated square-pyramidal coordination sphere, which make it beneficial to active the substrates (sulfide and H_2_O_2_). While in TeW_6_Cu, the Cu^II^ center is six-coordinated with an octahedra coordination sphere, and the Cu^II^ center is coordination-saturated, resulting in strong coordination of the ligand to Cu. The results make the Cu^II^ in TeW_6_Cu is less active to the substrates. So the activity of Te_2_W_8_Cu_2_ is higher than TeW_6_Cu. Based on the above results, Te_2_W_8_Cu_2_ can be proved as the best optimal catalyst for the oxidation reaction under this conditions. Moreover, the heterogeneous nature of Te_2_W_8_Cu_2_ was also investigated by a leaching experiment. As shown in Fig. 3, when the conversion of 1a reached to about 38% at 0.5 h, the catalyst was filtered off and the filtrate continued to react under the same conditions for 6 h, the reaction almost stopped and the conversion of 1a increased slightly due to the transformation of 1a itself without catalyst under such reaction conditions. The result revealed that the catalyst of Te_2_W_8_Cu_2_ is a heterogeneous catalyst in this reaction and stable without leaching under the optimized conditions.

As far as we know, the mechanism of catalytic oxidation reaction using H_2_O_2_ as oxidant usually shows two different pathways, the radical pathway and the peroxo species pathways. To further understand the mechanism of our system, the oxidation of thioethers using H_2_O_2_ as oxidant and Te_2_W_8_Cu_2_ as catalyst, radical trap experiments on the oxidation of methyl(phenyl)sulfane were employed (ESI, Table S3†). The reaction was carried out under the optimized conditions, after adding Ph_2_NH as oxygen-radical scavenger, 1,4-benzoquinone as superoxide (˙O^2−^/˙O_2_H scavenger) and *tert*-butyl alcohol as a hydroxyl radical scavenger,^28^ no obvious changes on the conversion and selectivity were observed (ESI, Table S3†). The results indicate that the mechanism of the reaction is not a radical pathway, which is consistent with previous literature that POMs tend to form peroxo-metal species in the presence of H_2_O_2_.^29^ So, we proposed that an active peroxo species formed during the reaction by using H_2_O_2_ as oxidant and Te_2_W_8_Cu_2_ as catalyst, although it has not been tested in this work.

According to the study of the catalytic performance of different catalysts for selective oxidation of thioethers, we noticed that the tungstotellurates(vi) Te_2_W_8_Cu_2_, TeW_6_Cu and TeW_6_ shown good catalytic potentials for this system, we think the {Te^VI^O_6_} heterogroup may sufficiently affect the redox property of the tungstotellurate(vi) catalysts which might be good for their catalytic activity of selective oxidation of thioethers. Moreover, the copper-containing tungstotellurates(vi) Te_2_W_8_Cu_2_ and TeW_6_Cu present better catalytic activity than the classic Anderson–Evans type tungstotellurates(vi) TeW_6_, which means introducing Cu^II^ centers in to the POM based complexes can improve the catalytic activity of tungstotellurates(vi), especially the Te_2_W_8_Cu_2_ with two Cu^II^ centers. Because the heterogeneous catalyst Te_2_W_8_Cu_2_ shows best catalytic activities for the selective oxidation of thioethers (Table 1), we studied systematically the catalytic performance of selective oxidation of different thioethers to corresponding sulfoxides using H_2_O_2_ (30%) (1.2 equiv.) as oxidant. A variety of organic sulfur compounds were subjected to this highly chemoselective catalytic system. As shown in Table 2, all of the desired sulfoxides products were obtained in moderate to good yield. Surprisingly, electron-donating as well as electron-withdrawing substituted phenyl ring thioanisole gave the desired sulfoxides (2a–2f) in high yields with good selectivities and high H_2_O_2_ utilization rate. Ethyl phenyl sulfide 1g can be converted into ethyl phenyl sulfoxide 2g with 99% conversion and 93% selectivity. The sterically more hindered substrate diphenylsulfane 1h were also investigated in this catalytic system. Fortunately, the corresponding product 3h was obtained in moderate yield. It is worth noting that less reactive dibutylsulfane 1i could be efficiently oxidized into the desired sulfoxides 2i in high yields with 98% selectivity.

**Table 2:** Selective oxidation of thioethers to sulfoxides^a^

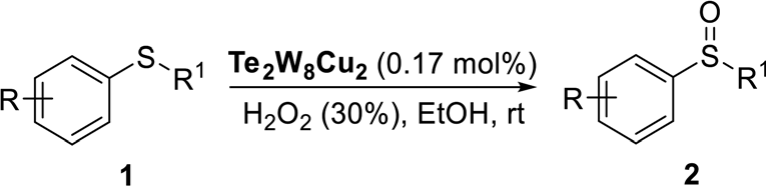					
Entry	Substrate 1	Conv.^b^ (%)	Product 2	Sel^b^. (%)	UR (%) (H_2_O_2_)
	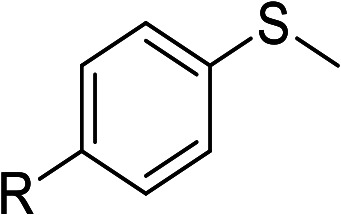		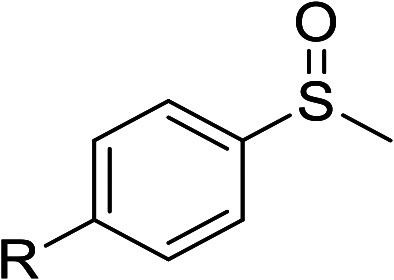		
1	R = H	99	2a	96	80
2	R = Me	99	2b	95	79
3	R = MeO	99	2c	98	81
4^c^	R = Ac	99	2d	91	75
5^c^	R = Cl	99	2e	93	77
6^c^	R = NO_2_	95	2f	90	71
	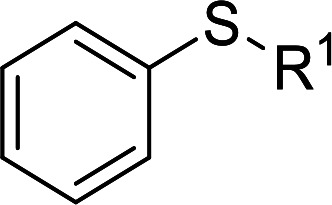		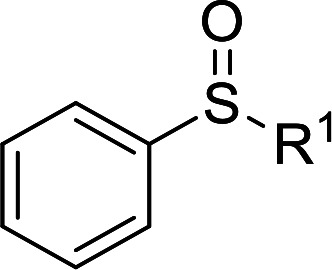		
7	R^1^ = Et	99	2g	93	77
8^d^	R^1^ = Ph	90	2h	66	54
	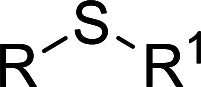		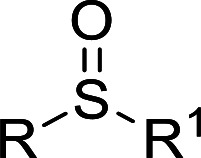		
9	R = R^1^ = *n*Bu	99	2i	98	81

a Reaction conditions: 1 (0.5 mmol), catalyst Te_2_W_8_Cu_2_ (0.17 mol%), EtOH (1 ml), H_2_O_2_ (30%) (1.2 equiv.), 8 h.

b Isolated yield.

c 12 h.

d 15 h.

Furthermore, we also explored its catalytic activity for selective oxidation of different thioethers to corresponding sulfones using H_2_O_2_ (30%) (2.5 equiv.). As shown in Table 3, a broad array of organic sulfur compounds worked well to afford the corresponding sulfones in good to excellent yields (Table 3, entries 1–9). It is worth noting that sterically hindered substrate diphenylsulfane 1h and less reactive dibutylsulfane 1i gave the corresponding sulfones 3h and 3i in 71% and 99% selectivity, respectively. Interestingly, we noticed that the molar ratio of H_2_O_2_ and Te_2_W_8_Cu_2_ plays a very important role for the selectivity of thioethers to corresponding sulfoxides and sulfones. This results are much better than other catalytic system using H_2_O_2_ as oxidant, and in this work we have developed a good method to improve the utilization ratio and reduce the consumption of H_2_O_2_ with good catalytic selectivity in the oxidation reaction of thioethers by using Te_2_W_8_Cu_2_ as catalyst. It is interesting to see that different oxidized products can be performed in high selectivity by using different solvents. The reason could be that the hydrogen bond and interaction between the reactants and solvents are different in ethanol and acetonitrile, which could solvate H_2_O_2_ effectively and thereby reduce its availability at the surface of catalyst.

**Table 3:** Selective oxidation of thioethers to sulfones^a^

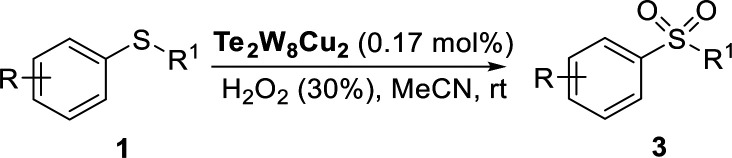					
Entry	Substrate 1	Conv.^b^ (%)	Product 3	Sel.^b^ (%)	UR (%) (H_2_O_2_)
	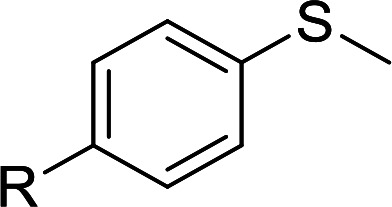		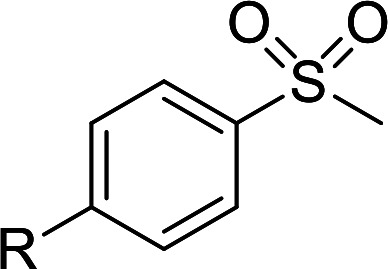		
1	R = H	99	3a	99	79
2	R = Me	99	3b	99	79
3	R = MeO	99	3c	99	79
4^c^	R = Ac	99	3d	98	78
5^c^	R = Cl	99	3e	97	77
6^c^	R = NO_2_	99	3f	96	77
	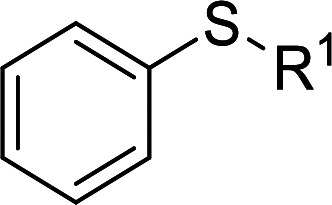		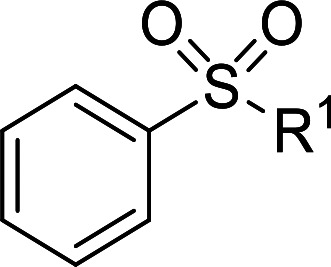		
7	R^1^ = Et	99	3g	99	79
8	R^1^ = Ph	96	3h	71	54
	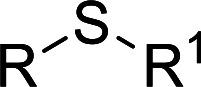		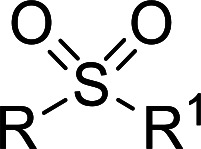		
9^d^	R = R^1^ = *n*Bu	99	3i	99	79

a Reaction conditions: 1 (0.5 mmol), catalyst Te_2_W_8_Cu_2_ (0.17 mol%), MeCN (1 ml), H_2_O_2_ (30%) (2.5 equiv.), rt, 6 h.

b Isolated yield.

c 10 h.

d 40 °C, 10 h.

**Fig. 4:**
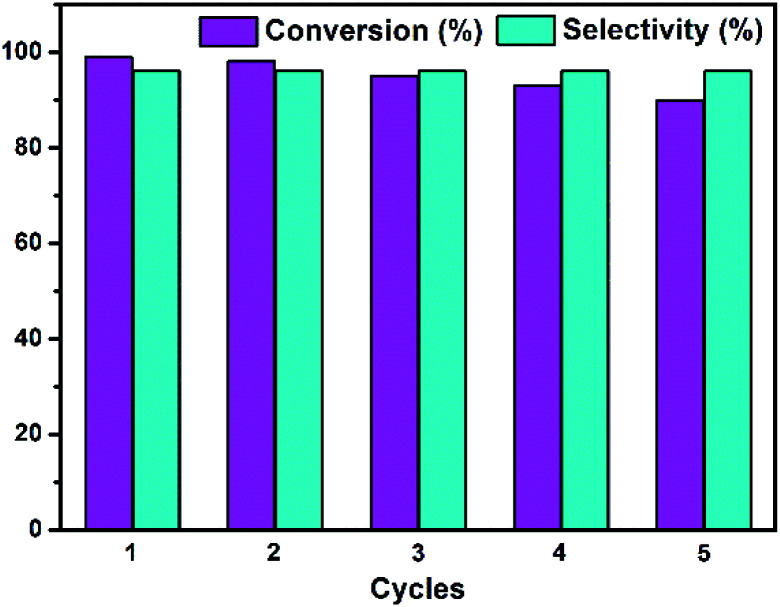
Recycle test for the oxidation of thioanisole 1a to 2a by Te_2_W_8_Cu_2_. (Reaction conditions: 1a (0.5 mmol) Te_2_W_8_Cu_2_ (0.17 mol%), 30% H_2_O_2_ (1.2 equiv.), EtOH (1.0 ml) at rt.).

As mentioned above, we have investigated the heterogeneous nature of Te_2_W_8_Cu_2_ by a leaching experiment, which reveals that the catalyst is a heterogeneous catalyst in this reaction and stable without leaching under the optimized condition. Therefore, in order to the service life and reusability of the catalyst, we also evaluated the recyclability and stability of Te_2_W_8_Cu_2_ for the selective oxidation of thioethers. As shown in Fig. 4, the catalytic reactivity of Te_2_W_8_Cu_2_ remains without obviously decreasing the conversion and selectivity after 5 cycles, which means the catalyst is recyclable and reusable after the reaction. The results of comparison of FT-IR spectra of the catalyst before and after the recycle reactions also confirms that the structure of Te_2_W_8_Cu_2_ is stable without decomposition in the reaction (ESI, Fig. S5†). This results indicate that the structure of Te_2_W_8_Cu_2_ is very favourable for this catalytic system. The recyclability and stability of TeW_6_ and TeW_6_Cu were also evaluated (ESI, Fig. S6 and S7†). Interestingly, the stability order of the catalyst is Te_2_W_8_Cu_2_ > TeW_6_Cu > TeW_6._ The results indicate that the new catalyst becomes more stable than POMs when the Cu^II^ centers were introduced into the POMs.

## Conclusions

In summary, we have synthesized two new copper-containing tungstotellurates(vi) Te_2_W_8_Cu_2_ and TeW_6_Cu by a simple one pot reaction which were characterized by single-crystal X-ray diffraction(XRD), powder XRD, FT-IR spectroscopy, elemental analysis, and thermogravimetric analysis in solid state. The catalytic properties of the tungstotellurates(vi) for the selective oxidation of thioethers were also studied systematically. The compound Te_2_W_8_Cu_2_ showed very high catalytic activity for the selective oxidation of thioethers to sulfoxides (conversion: up to 99%, selectivity: up to 98%) and sulfones (conversion: up to 99%, selectivity: up to 98%) by H_2_O_2_ under ambient conditions. In addition, the Te_2_W_8_Cu_2_ catalyst was stable in the environmentally benign catalytic system and could be reused at least five cycles without a significant loss of reactivity on the model reaction. This work offer a new strategy for the selective oxidation of thioethers by using tungstotellurates(vi) as heterogeneous catalyst, and the utilization ratio of the oxidant H_2_O_2_ is up to about 80%.

## Conflicts of interest

There are no conflicts to declare.

## Supplementary Material

RA-010-D0RA02609C-s001

RA-010-D0RA02609C-s002
