# Supplementary material for: Copper(ii)-containing tungstotellurates(vi): syntheses, structures and their catalytic performances in selective oxidation of thioethers
Source: RSC Adv. 2020 Jun 12;10(38):22515–21. doi: 10.1039/d0ra02609c (PMC9054611; doi:10.1039/d0ra02609c)
Supplement: RA-010-D0RA02609C-s001 [file RA-010-D0RA02609C-s001.pdf]

Electronic Supplementary Material (ESI) for RSC Advances.
This journal is © The Royal Society of Chemistry 2020

Supplemental material

General Information

Experimental Procedures of Catalytic Study

Fig S1. Packing diagram structure of $\mathbf{Te_2W_8Cu_2}$ and $\mathbf{TeW_6Cu}$

Fig S2. IR spectra of $\mathbf{Te_2W_8Cu_2}$ and $\mathbf{TeW_6Cu}$

Fig S3. PXRD curve of $\mathbf{Te_2W_8Cu_2}$ and $\mathbf{TeW_6Cu}$

Fig S4. Thermogavimetric Analysis of $\mathbf{Te_2W_8Cu_2}$ and $\mathbf{TeW_6Cu}$

Fig S5. IR spectra of fresh $\mathbf{Te_2W_8Cu_2}$ and recovered $\mathbf{Te_2W_8Cu_2}$

Fig S6. IR spectra of fresh $\mathbf{TeW_6Cu}$ and recovered $\mathbf{TeW_6Cu}$

Fig S7. IR spectra of fresh $\mathbf{TeW_6}$ and recovered $\mathbf{TeW_6}$

Fig S8. Recycle test for the oxidation of thioanisole **1a** to **2a** by $\mathbf{TeW_6Cu}$

Fig S9. Recycle test for the oxidation of thioanisole **1a** to **2a** by $\mathbf{TeW_6}$

Table S1. Crystal data and structure refinement for $\mathbf{Te_2W_8Cu_2}$ and $\mathbf{TeW_6Cu}$

Table S2. Selected bond lengths and angles for $\mathbf{Te_2W_8Cu_2}$ and $\mathbf{TeW_6Cu}$

Table S3. Effect of radical trap on the oxidation reaction

NMR Spectroscopic data and Spectra of products

General Information

All the solvents and reagents were purchased from the suppliers and used without further purification unless otherwise stated. Unless stated otherwise, all reactions were carried out in pressure tube (10 mL) with a sealing cap under air atmosphere. The yields reported are for isolated yields unless otherwise stated. ^{1}H NMR (500 MHz) and ^{13}C NMR (125 MHz) spectra were recorded in $CDCl_3$ at room temperature on a Bruker using TMS as the internal reference. IR spectra were obtained on a FT-IR spectrometer using KBr plates (thin film). Column chromatography was performed on silica gel (200-400 mesh). GC analyses were performed on a Shimadzu GC-2014 equipped with a capillary column (HP-5 30 m × 0.25 μm × 0.32mm) using a flame ionization detector.

Experimental Procedures of Catalytic Study

General procedure for the formation of sulfoxides (2a)

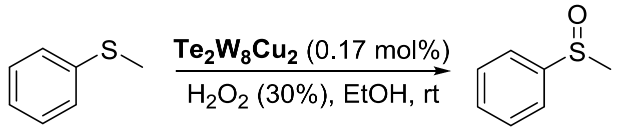

1a **2a**

A mixture of methyl(phenyl)sulfane **1a** (62.0 mg, 0.50 mmol), 30% H_2O_2 (0.6 mmol, 1.2 equiv.) in EtOH (1 mL), then add $Na_{12}[Te_2W_8O_{38}Cu_2(H_2O)_2]\cdot 7H_2O$ (0.17 mol%) in a pressure tube (10 mL) with a sealing cap under air atmosphere. The reaction mixture was vigorously stirred at room temperature until the compietion as indicated by TLC. The reaction mixture was filtered through a short pad of celite and quenched with water. Then the mixture was extracted by dichloromethane (10 ml x 3), dried over $MgSO_4$, and evaporated under reduced pressure to afford the crude product, which was further purified by flash chromatography on silicagel with n-hexane/EtOAc to give *tert*-butyl (benzoyloxy)carbamate (**2a**, 67.0 mg, 96%).

General procedure for the formation of sulfoxides (3a)

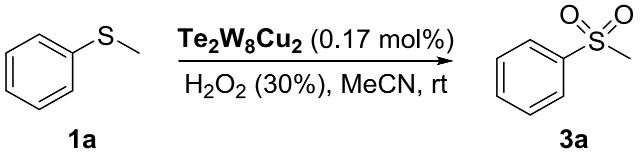

1a **3a**

A mixture of methyl(phenyl)sulfane **1a** (62.0 mg, 0.50 mmol), 30% H_2O_2 (1.25 mmol, 2.5 equiv.) in MeCN (1 mL), then add $Na_{12}[Te_2W_8O_{38}Cu_2(H_2O)_2]\cdot 7H_2O$ (0.17 mol%) in a pressure tube (10 mL) with a sealing cap under air atmosphere. The reaction mixture was vigorously stirred at room temperature until the compietion as indicated by TLC. The reaction mixture was filtered through a

short pad of celite and quenched with water. Then the mixture was extracted by dichloromethane (10 ml x 3), dried over $MgSO_4$, and evaporated under reduced pressure to afford the crude product, which was further purified by flash chromatography on silicagel with n-hexane/EtOAc to give *tert*-butyl (benzoyloxy)carbamate (**3a**, 77.0 mg, 99%).

After the 5 cycles of reaction, the catalyst was filtered off and washed with dichloromethane, dired in oven at 50 °C for 5 h. The Fourier transform infrared (FT-IR) spectra of (a) "fresh" **$Te_2W_8Cu_2$** and (b) "recovered" **$Te_2W_8Cu_2$** showed that the **$Te_2W_8Cu_2$** was stable in this reaction system by the characteristic peaks appeared at 937, 879, 690, 610, 552, 450 cm^{-1}.

a)

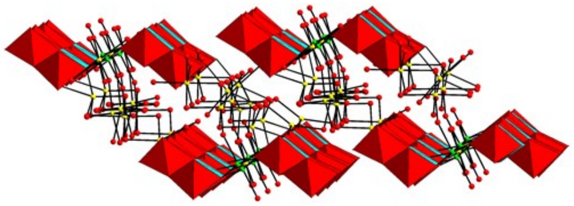

b)

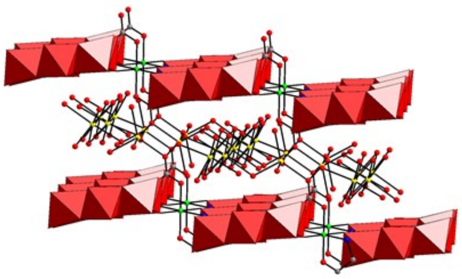

Fig S1. Packing diagram structure of **$Te_2W_8Cu_2$** and **TeW_6Cu**

Note: The two structures were refined by the *SHELXTL* program package (Bruker), and all structures were solved by direct methods and refined by the full-matrix least-squares method ($\Sigma w(|F_o|^2 - |F_c|^2)^2$)). The absorption correction was carried by SADABS-2013.4-1 (Bruker APEX 2) after the diffraction data was collected and reduced. We would like to point out that it seems to be very difficult to model all H atoms of the water molecules in the two POM structures with accurate location due to the big difference of diffraction properties between the heavy W atoms and light H atoms in the same structures, which is very common in POM chemistry. Because the accurate locations of most these hydrogen atoms are still not very clear based on the current diffraction data (Mo K\a source), and the H atoms location information are not very important for us to understand the core structures of these POM compounds, the hydrogen atoms of waters were not incorporated in the refinement. In the two POM compounds, the O atoms labeled as O1w-O40w in compound **1** and O1w-O10w in compound **2** are all water molecules without H atoms, and there are no bridging species O2-, OH- in the structures.

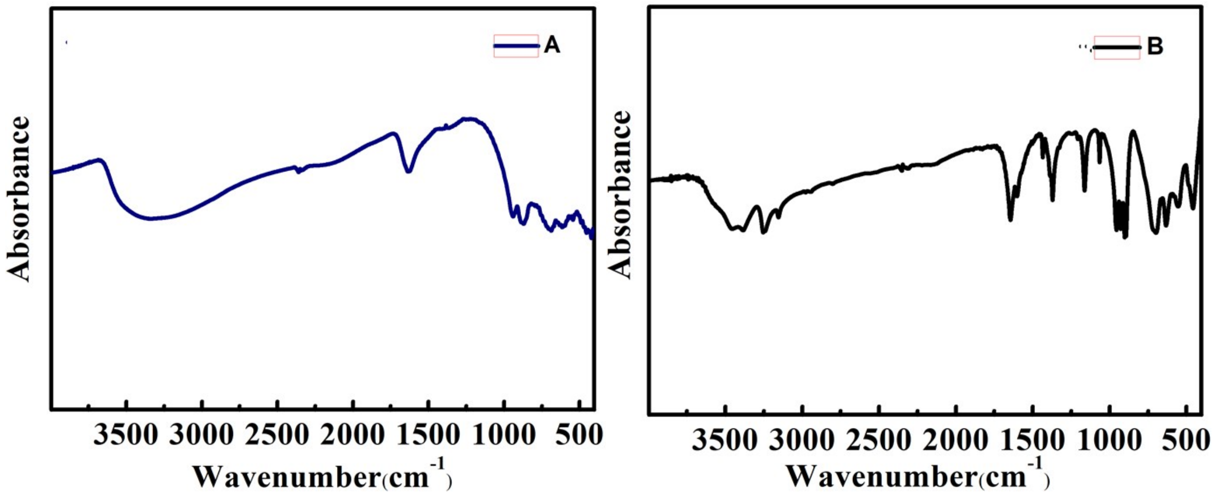

Fig S2. IR spectra of $\mathbf{Te_2W_8Cu_2}$ (A) and $\mathbf{TeW_6Cu}$ (B).

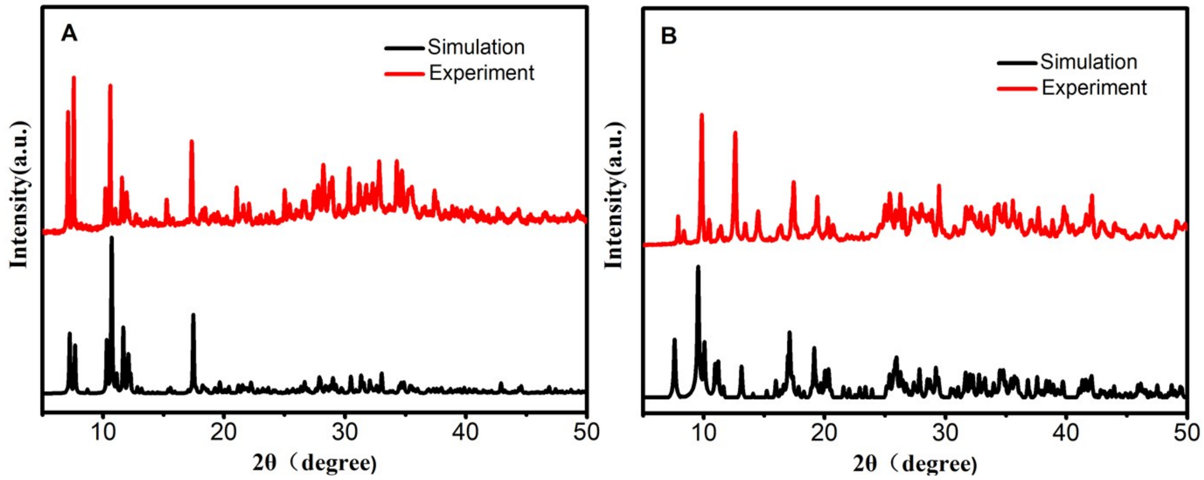

Fig S3. PXRD curve of $\mathbf{Te_2W_8Cu_2}$ (A) and $\mathbf{TeW_6Cu}$ (B).

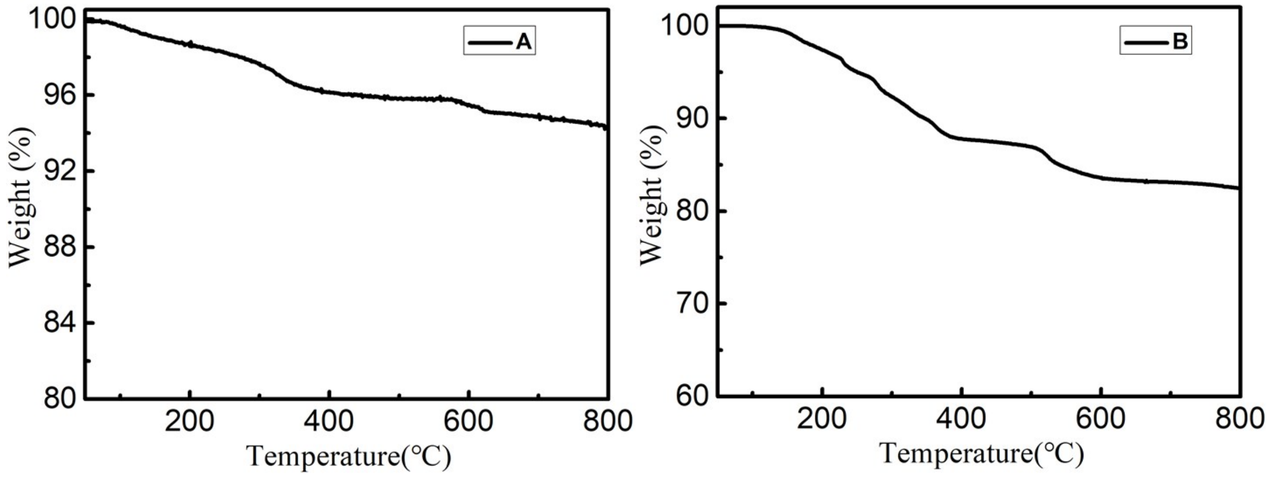

Fig S4. Thermogavimetric Analysis of $\mathbf{Te_2W_8Cu_2}$ (A) and $\mathbf{TeW_6Cu}$ (B).

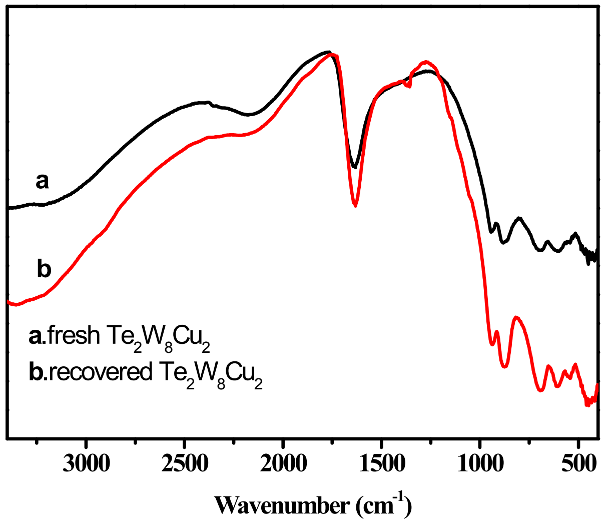

Figure S5. IR spectra offresh $\mathbf{Te_2W_8Cu_2}$ (a) and recovered $\mathbf{Te_2W_8Cu_2}$ (b)

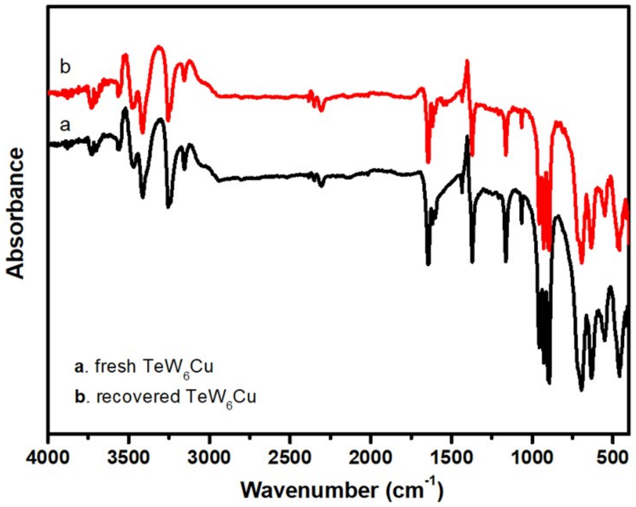

Figure S6. IR spectra of fresh **TeW_6Cu** (a) and recovered **TeW_6Cu** (b)

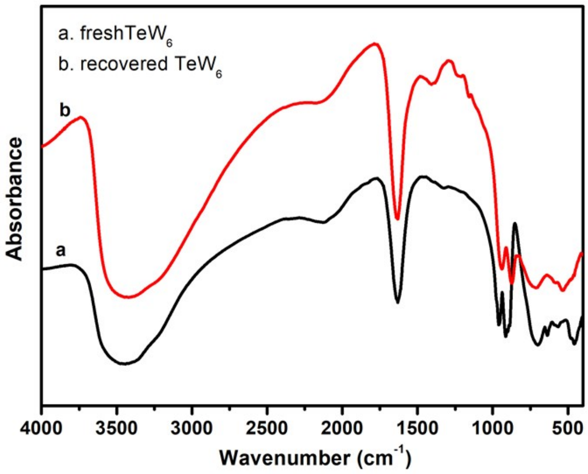

Figure S6. IR spectra of fresh **TeW_6** (a) and recovered **TeW_6** (b)

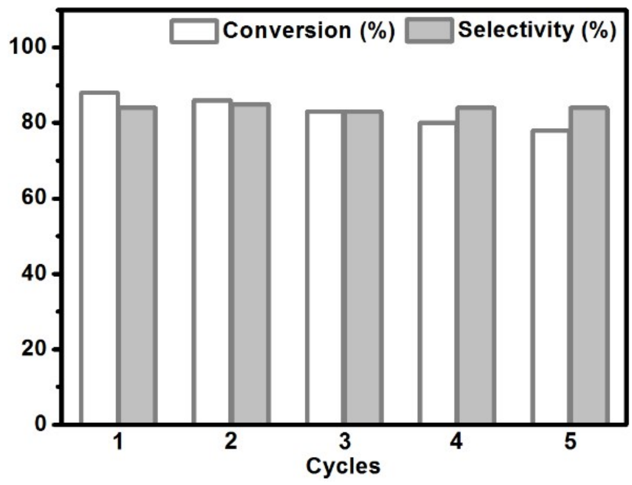

Fig S8. Recycle test for the oxidation of thioanisole **1a** to **2a** by $\mathbf{TeW_6Cu}$

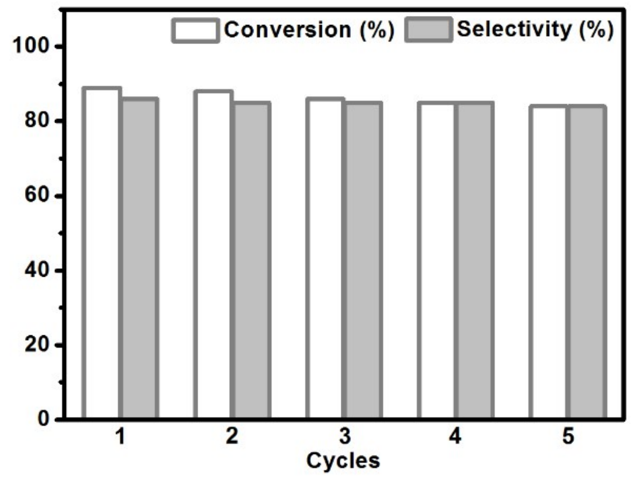

Fig S9. Recycle test for the oxidation of thioanisole **1a** to **2a** by $\mathbf{TeW_6}$

Table S1. Crystal data and structure refinement for compound $\mathbf{Te_2W_8Cu_2}$ and $\mathbf{TeW_6Cu}$.

Empirical formula	$Cu_2H_{80}Na_{12}O_{78}Te_2W_8$	$C_4H_{48}CuN_2Na_6O_{48}TeW_6$
Formula weight, g/mol	3457.60	2324.62
Crystal system	Triclinic	Triclinic
Space group	P-1	P-1
a, Å	13.6212(15)	10.4840(8)
b, Å	16.7386(19)	10.6800(8)
c, Å	17.399(2)	12.0437(9)
α, °	95.705(3)	76.3600(17)
β, °	103.882(3)	78.2885(17)
γ, °	112.109(3)	61.5611(16)
Volume, $Å^3$	3487.7(7)	1145.84(15)
Z	2	1
D_{calc}, g/cm^3	3.292	3.369
Absorption coefficient, mm^{-1}	14.702	16.219
F(000)	3180	1061
Theta range for data collection, °	1.233 to 28.483	1.750 to 28
Completeness to Θ_{max}	99.9 %	99.8
Reflections collected	42718	10433
Independent reflections	17436	4020
R(int)	0.0545	0.0472
Absorption correction	Semi-empirical from equivalents	Semi-empirical from equivalents
Data / restraints / parameters	17436 / 2088 / 922	5514 / 455 / 327
Goodness-of-fit on F^2	1.038	1.032
R_1,[a] wR_2[b] ($I > 2\sigma(I)$)	$R_1 = 0.0356$, $wR_2 = 0.0988$	$R_1 = 0.0364$, $wR_2 = 0.0905$
R_1,[a] wR_2[b] (all data)	$R_1 = 0.0441$, $wR_2 = 0.1056$	$R_1 = 0.0375$, $wR_2 = 0.0914$
Largest diff. peak and hole, e/$Å^3$	1.656 and -2.621 e.A^-3	4.092 and -3.632

[a] $R_1 = \Sigma||F_o| - |F_c||/\Sigma|F_o|$. [b] $wR_2 = [\Sigma w(F_o^2 - F_c^2)^2/\Sigma w(F_o^2)^2]^{1/2}$.

Table S2. Selected bond lengths and angles for $\mathbf{Te_2W_8Cu_2}$ and $\mathbf{TeW_6Cu}$

$\mathbf{Te_2W_8Cu_2}$		$\mathbf{TeW_6Cu}$	
Cu(1)-O(30)	1.923(4)	Cu(1)-O(12)	1.947(5)
Cu(1)-O(26)#1	1.945(4)	Cu(1)-O(12)#1	1.947(5)
Cu(1)-O(20)	1.977(4)	Cu(1)-N(1)	1.991(7)
Cu(1)-O(20)#1	1.982(4)	Cu(1)-N(1)#1	1.991(7)
Cu(1)-O(2W)	2.327(5)	O(2)-Te(1)	1.921(5)
Cu(1)-Cu(1)#1	3.0304(13)	O(5)-Te(1)	1.937(5)
Cu(2)-O(1)	1.914(4)	O(6)-Te(1)	1.937(5)
Cu(2)-O(13)#2	1.967(4)		
Cu(2)-O(5)#2	1.970(4)		
Cu(2)-O(5)	1.991(4)		
Cu(2)-O(1W)	2.341(4)		
Cu(2)-Cu(2)#2	3.0269(13)		
O(4)-Te(1)	1.968(4)		
O(5)-Te(1)	1.920(4)		
O(6)-Te(1)	1.968(4)		
O(7)-Te(1)	1.954(4)		
O(10)-Te(1)	1.889(4)		
O(11)-Te(1)	1.881(4)		
O(20)-Te(2)	1.917(4)		
O(22)-Te(2)	1.885(4)		
O(23)-Te(2)	1.953(4)		
O(28)-Te(2)	1.969(4)		
O(34)-Te(2)	1.886(4)		
O(37)-Te(2)	1.963(4)		
O(30)-Cu(1)-O(26)#1	90.51(17)	O(12)-Cu(1)-O(12)#1	180
O(30)-Cu(1)-O(20)	96.77(17)	O(12)-Cu(1)-N(1)	84.6(2)
O(26)#1-Cu(1)-O(20)	162.97(17)	O(12)#1-Cu(1)-N(1)	95.4(2)
O(30)-Cu(1)-O(20)#1	170.84(17)	O(12)-Cu(1)-N(1)#1	95.4(2)
O(26)#1-Cu(1)-O(20)#1	90.30(17)	O(12)#1-Cu(1)-N(1)#1	84.6(2)
O(20)-Cu(1)-O(20)#1	80.11(18)	O(2)-Te(1)-O(2)#5	180
O(30)-Cu(1)-O(2W)	88.86(18)	O(2)-Te(1)-O(6)	85.0(2)
O(26)#1-Cu(1)-O(2W)	98.53(17)	O(2)#5-Te(1)-O(6)	95.0(2)
O(20)-Cu(1)-O(2W)	96.99(16)	O(2)-Te(1)-O(6)#5	95.0(2)
O(20)#1-Cu(1)-O(2W)	100.05(17)	O(2)#5-Te(1)-O(6)#5	85.0(2)
O(1)-Cu(2)-O(13)#2	90.26(17)	O(6)-Te(1)-O(6)#5	180
O(1)-Cu(2)-O(5)#2	170.03(17)	O(2)-Te(1)-O(5)	95.1(2)
O(13)#2-Cu(2)-O(5)#2	90.68(16)	O(2)#5-Te(1)-O(5)	84.9(2)
O(1)-Cu(2)-O(5)	96.35(17)	O(6)-Te(1)-O(5)	85.3(2)
O(13)#2-Cu(2)-O(5)	163.92(17)	O(6)#5-Te(1)-O(5)	94.7(2)
O(5)#2-Cu(2)-O(5)	80.33(18)	O(2)-Te(1)-O(5)#5	84.9(2)
O(1)-Cu(2)-O(1W)	91.42(17)	O(2)#5-Te(1)-O(5)#5	95.1(2)
O(13)#2-Cu(2)-O(1W)	97.85(16)	O(6)-Te(1)-O(5)#5	94.7(2)
O(5)#2-Cu(2)-O(1W)	98.28(16)	O(6)#5-Te(1)-O(5)#5	85.3(2)
O(5)-Cu(2)-O(1W)	96.63(16)	O(5)-Te(1)-O(5)#5	180
Te(1)-O(5)-Cu(2)#2	125.8(2)		

Te(1)-O(5)-Cu(2)	127.4(2)		
Cu(2)#2-O(5)-Cu(2)	99.67(18)		
Te(2)-O(20)-Cu(1)	126.9(2)		
Te(2)-O(20)-Cu(1)#1	125.5(2)		
Cu(1)-O(20)-Cu(1)#1	99.89(18)		
O(11)-Te(1)-O(10)	96.77(17)		
O(11)-Te(1)-O(5)	94.19(17)		
O(10)-Te(1)-O(5)	94.08(17)		
O(11)-Te(1)-O(7)	93.70(17)		
O(10)-Te(1)-O(7)	93.04(17)		
O(5)-Te(1)-O(7)	168.68(17)		
O(11)-Te(1)-O(4)	85.64(16)		
O(10)-Te(1)-O(4)	175.11(17)		
O(5)-Te(1)-O(4)	89.98(17)		
O(7)-Te(1)-O(4)	82.54(16)		
O(11)-Te(1)-O(6)	175.34(17)		
O(10)-Te(1)-O(6)	84.82(17)		
O(5)-Te(1)-O(6)	90.05(17)		
O(7)-Te(1)-O(6)	81.83(16)		
O(4)-Te(1)-O(6)	92.45(16)		
O(22)-Te(2)-O(34)	96.80(17)		
O(22)-Te(2)-O(20)	93.36(17)		
O(34)-Te(2)-O(20)	94.64(17)		
O(22)-Te(2)-O(23)	93.08(17)		
O(34)-Te(2)-O(23)	92.56(17)		
O(20)-Te(2)-O(23)	169.71(17)		
O(22)-Te(2)-O(37)	175.39(17)		
O(34)-Te(2)-O(37)	85.39(17)		
O(20)-Te(2)-O(37)	90.50(17)		
O(23)-Te(2)-O(37)	82.75(17)		
O(22)-Te(2)-O(28)	84.94(17)		
O(34)-Te(2)-O(28)	174.44(17)		
O(20)-Te(2)-O(28)	90.52(17)		
O(23)-Te(2)-O(28)	82.06(17)		
O(37)-Te(2)-O(28)	92.51(16)		

Table S3. Effect of radical trap on the oxidation of methyl(phenyl)sulfane catalyzed by {$Te_2W_8Cu_2$}.[a]

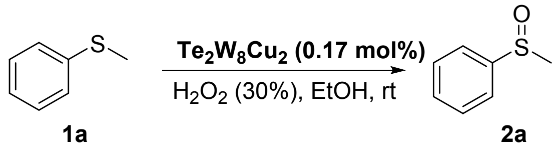

Entry	Radical trap	mmol	Conv. (%)[b]	Sel. (%)[b]
1	-	-	99	96
2	Ph_2NH	0.6	99	96
3	*p*-benzoquinone	0.6	99	94
4	*tert*-butyl alcohol	0.6	99	93

[a]Reaction conditions: **1a** (0.5 mmol), catalyst (0.17 mol%, 2.5 mg ± 0.5 mg), H_2O_2 30% (1.2 equiv., 0.6 mmol), EtOH (1 ml), rt, 8 h. [b]Yields determined by GC analysis using 1-adamantanol as an internal standard.

NMR Spectroscopic Data of Products

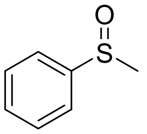

Table 2, entry 1

(methylsulfinyl)benzene (2a). Oil (67 mg, 96% yield); R_f= 0.35 (hexane:EtOAc = 2:1); ^{1}H NMR (500 MHz, $CDCl_3$) δ 7.63–7.59 (m, 2H), 7.52–7.43 (m, 3H), 2.68 (s, 3H); ^{13}C NMR (125 MHz, $CDCl_3$) δ 145.5, 130.8, 129.2, 123.3, 43.8.

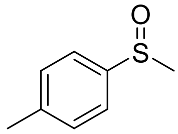

Table 2, entry 2

1-methyl-4-(methylsulfinyl)benzene (2b). Oil (73 mg, 95% yield); R_f = 0.4 (hexane:EtOAc = 2:1); ^{1}H NMR (500 MHz, $CDCl_3$) δ 7.53 (d, J = 8.1 Hz, 2H), 7.33 (t, J = 12.1 Hz, 2H), 2.69 (s, 3H), 2.40 (s, 3H); ^{13}C NMR (125 MHz, $CDCl_3$) δ 142.3, 141.4, 129.9, 123.5, 43.9, 21.3.

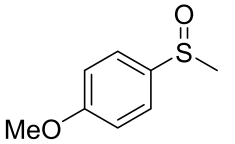

Table 2, entry 3

1-methoxy-4-(methylsulfinyl)benzene (2c). Oil (83 mg, 98% yield); R_f= 0.30 (hexane:EtOAc = 2:1); ^{1}H NMR (500 MHz, $CDCl_3$) δ 7.56–7.49 (m, 2H), 7.00–6.92 (m, 2H), 3.78 (s, 3H), 2.63 (s, 3H); ^{13}C NMR (125 MHz, $CDCl_3$) δ 161.7, 136.4, 125.2, 114.6, 55.3, 43.8.

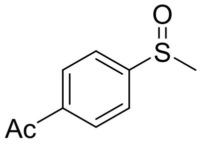

Table 2, entry 4

1-(4-(methylsulfinyl)phenyl)ethan-1-one (2d). White sliod (82 mg, 91% yield); mp: 106-107 °C; R_f = 0.31 (hexane:EtOAc = 1:1); ^{1}H NMR (500 MHz, $CDCl_3$) δ 8.06 (d, J = 8.4 Hz, 2H), 7.71 (d, J = 8.4 Hz, 2H), 2.72 (s, 3H), 2.61 (s, 3H); ^{13}C NMR (125 MHz, $CDCl_3$) δ 197.0, 150.9, 139.0, 129.1, 123.7, 43.8, 26.7.

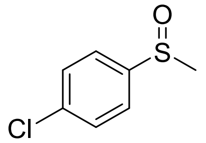

Table 2, entry 5

1-chloro-4-(methylsulfinyl)benzene (2e). Oil (81 mg, 93% yield); R_f= 0.35 (hexane:EtOAc = 2:1); ^{1}H NMR (500 MHz, $CDCl_3$) δ 7.59 (d, J = 8.4 Hz, 2H), 7.51 (d, J = 8.3 Hz, 2H), 2.71 (s, 3H); ^{13}C NMR (125 MHz, $CDCl_3$) δ 144.2, 137.2, 129.6, 124.9, 44.0.

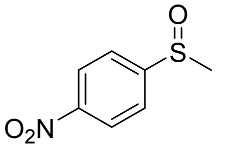

Table 2, entry 6

1-(methylsulfinyl)-4-nitrobenzene (2f). White sliod (79 mg, 85% yield); mp: 152-153 °C; R_f= 0.29 (hexane:EtOAc = 1:1); ^{1}H NMR (500 MHz, $CDCl_3$) δ 8.40 (d, J = 8.7 Hz, 2H), 7.85 (d, J = 8.7 Hz, 2H), 2.80 (s, 3H); ^{13}C NMR (125 MHz, $CDCl_3$) δ 153.2, 149.5, 124.6, 124.4, 43.8.

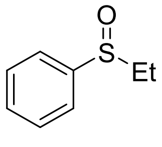

Table 2, entry 7

(ethylsulfinyl)benzene (2g). Oil (71 mg, 93% yield); R_f= 0.39 (hexane:EtOAc = 3:1); ^{1}H NMR (500 MHz, $CDCl_3$) δ 7.62–7.55 (m, 2H), 7.52–7.44 (m, 3H), 2.87 (dq, J = 14.8, 7.4 Hz, 1H), 2.73 (dq, J = 14.7, 7.4 Hz, 1H), 1.16 (t, J = 7.4 Hz, 3H); ^{13}C NMR (125 MHz, $CDCl_3$) δ 143.3, 130.9, 129.1, 124.1, 50.2, 5.9.

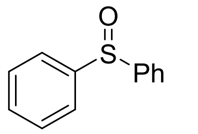

Table 2, entry 8

sulfinyldibenzene (2h). Oil (59 mg, 59% yield); R_f = 0.32 (hexane:EtOAc = 2:1); ^{1}H NMR (500 MHz, $CDCl_3$) δ 7.68–7.60 (m, 4H), 7.50–7.40 (m, 6H); ^{13}C NMR (125 MHz, $CDCl_3$) δ 145.6, 131.0, 129.3, 124.8.

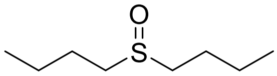

Table 2, entry 9

1-(butylsulfinyl)butane (2i). White sliod (79 mg, 98% yield); mp: 152-153 °C; R_f= 0.46 (hexane:EtOAc = 3:1); ^{1}H NMR (500 MHz, $CDCl_3$) δ 2.71–2.54 (m, 4H), 1.81–1.65 (m, 4H), 1.45 (pd, J = 13.7, 7.2 Hz, 4H), 0.93 (t, J = 7.4 Hz, 6H); ^{13}C NMR (125 MHz, $CDCl_3$) δ 52.1, 24.5, 22.0, 13.6.

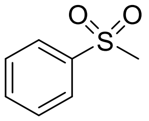

Table 3, entry 1

(methylsulfonyl)benzene (3a). Oil (77 mg, 99% yield); R_f = 0.51 (hexane:EtOAc = 3:1); ^{1}H NMR (500 MHz, $CDCl_3$) δ 7.94 (d, J = 7.4 Hz, 2H), 7.66 (t, J = 7.4 Hz, 1H), 7.57 (t, J = 7.7 Hz, 2H), 3.05 (s, 3H); ^{13}C NMR (125 MHz, $CDCl_3$) δ 140.5, 133.7, 129.3, 127.3, 44.5.

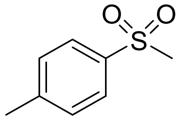

Table 3, entry 2

1-methyl-4-(methylsulfonyl)benzene (3b). White sliod (84 mg, 99% yield); mp: 83-84 °C; R_f= 0.53 (hexane:EtOAc = 3:1); ^{1}H NMR (500 MHz, $CDCl_3$) δ 7.79 (d, J = 8.2 Hz, 2H), 7.34 (d, J = 8.3 Hz, 2H), 3.01 (s, 3H), 2.42 (s, 3H); ^{13}C NMR (125 MHz, $CDCl_3$) δ 144.6, 137.6, 129.9, 127.3, 44.6, 21.6.

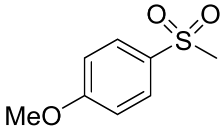

Table 3, entry 3

1-methoxy-4-(methylsulfonyl)benzene (3c). White sliod (92 mg, 99% yield); mp: 118-119 °C; R_f = 0.45 (hexane:EtOAc = 3:1); ^{1}H NMR (500 MHz, $CDCl_3$) δ 7.87 (d, J = 8.9 Hz, 2H), 7.03 (d, J = 8.9 Hz, 2H), 3.89 (s, 3H),

3.03 (s, 3H); ^{13}C NMR (125 MHz, $CDCl_3$) δ 163.7, 132.3, 129.5, 114.5, 55.7, 44.8.

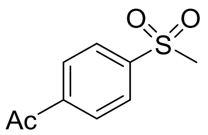

Table 3, entry 4

1-(4-(methylsulfonyl)phenyl)ethan-1-one (3d). White sliod (97 mg, 98% yield); mp: 128-129 °C; R_f = 0.39 (hexane:EtOAc = 3:1); ^{1}H NMR (500 MHz, $CDCl_3$) δ 8.11 (d, J = 8.4 Hz, 2H), 8.03 (d, J = 8.4 Hz, 2H), 3.07 (s, 3H), 2.65 (s, 3H); ^{13}C NMR (125 MHz, $CDCl_3$) δ 196.6, 144.2, 140.9, 129.1, 127.8, 44.3, 26.9.

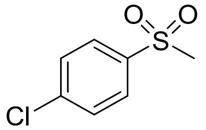

Table 3, entry 5

1-chloro-4-(methylsulfonyl)benzene (3e). White sliod (92 mg, 97% yield); mp: 95-96 °C; R_f= 0.42 (hexane:EtOAc = 3:1); ^{1}H NMR (500 MHz, $CDCl_3$) δ 7.86 (d, J = 8.6 Hz, 2H), 7.52 (d, J = 8.6 Hz, 2H), 3.03 (s, 3H); ^{13}C NMR (125 MHz, $CDCl_3$) δ 140.4, 139.0, 129.7, 128.9, 44.5.

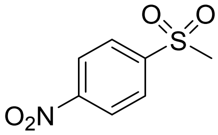

Table 3, entry 6

1-(methylsulfonyl)-4-nitrobenzene (3f). White sliod (96 mg, 96% yield); mp: 143-144 °C; R_f= 0.36 (hexane:EtOAc = 3:1); ^{1}H NMR (500 MHz, $CDCl_3$) δ 8.41 (d, J = 8.7 Hz, 2H), 8.15 (d, J = 8.7 Hz, 2H), 3.12 (s, 3H); ^{13}C NMR (125 MHz, $CDCl_3$) δ 150.8, 145.9, 128.9, 124.6, 44.2.

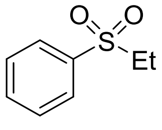

Table 3, entry 7

(ethylsulfonyl)benzene (3g). Oil (84 mg, 99% yield); R_f= 0.39 (hexane:EtOAc = 3:1); ^{1}H NMR (500 MHz, $CDCl_3$) δ 7.88 (d, J = 8.0 Hz, 2H), 7.64 (t, J = 7.4 Hz, 1H), 7.55 (t, J = 7.8 Hz, 2H), 3.10 (q, J = 7.4 Hz, 2H), 1.25 (t, J = 7.4 Hz, 3H); ^{13}C NMR (125 MHz, $CDCl_3$) δ 138.5, 138.5, 133.6, 133.6, 129.2, 128.1, 50.5, 7.4.

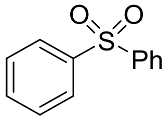

Table 3, entry 8

sulfonyldibenzene (3h). White sliod (74 mg, 68% yield); mp: 123-125 °C; R_f= 0.42 (hexane:EtOAc = 3:1); ^{1}H NMR (500 MHz, $CDCl_3$) δ 7.94 (d, J = 7.1 Hz, 4H), 7.55 (t, J = 7.4 Hz, 2H), 7.49 (t, J = 7.5 Hz, 4H); ^{13}C NMR (125 MHz, $CDCl_3$) δ 141.6, 133.2, 129.3, 127.6.

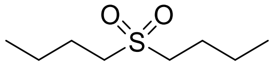

Table 3, entry 9

1-(butylsulfonyl)butane (3i). White sliod (88 mg, 99% yield); mp: 40-41 °C; R_f = 0.39 (hexane:EtOAc = 3:1); ^{1}H NMR (500 MHz, $CDCl_3$) δ 2.97–2.83 (m, 4H), 1.80–1.72 (m, 4H), 1.52–1.36 (m, 4H), 0.99–0.86 (m, 6H); ^{13}C NMR (125 MHz, $CDCl_3$) δ 52.4, 23.9, 21.7, 13.5.

^{1}H NMR and ^{13}C NMR of **2a.**

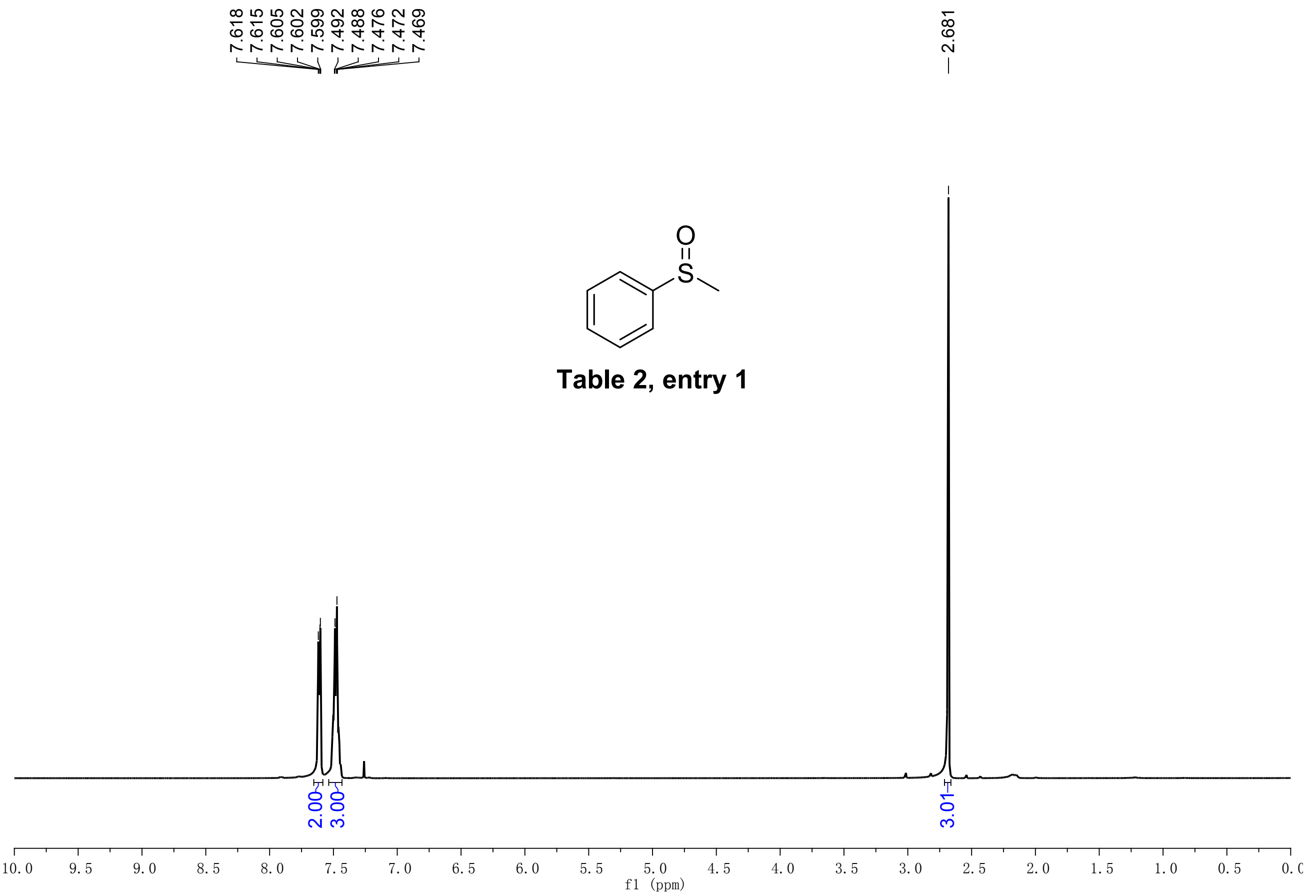

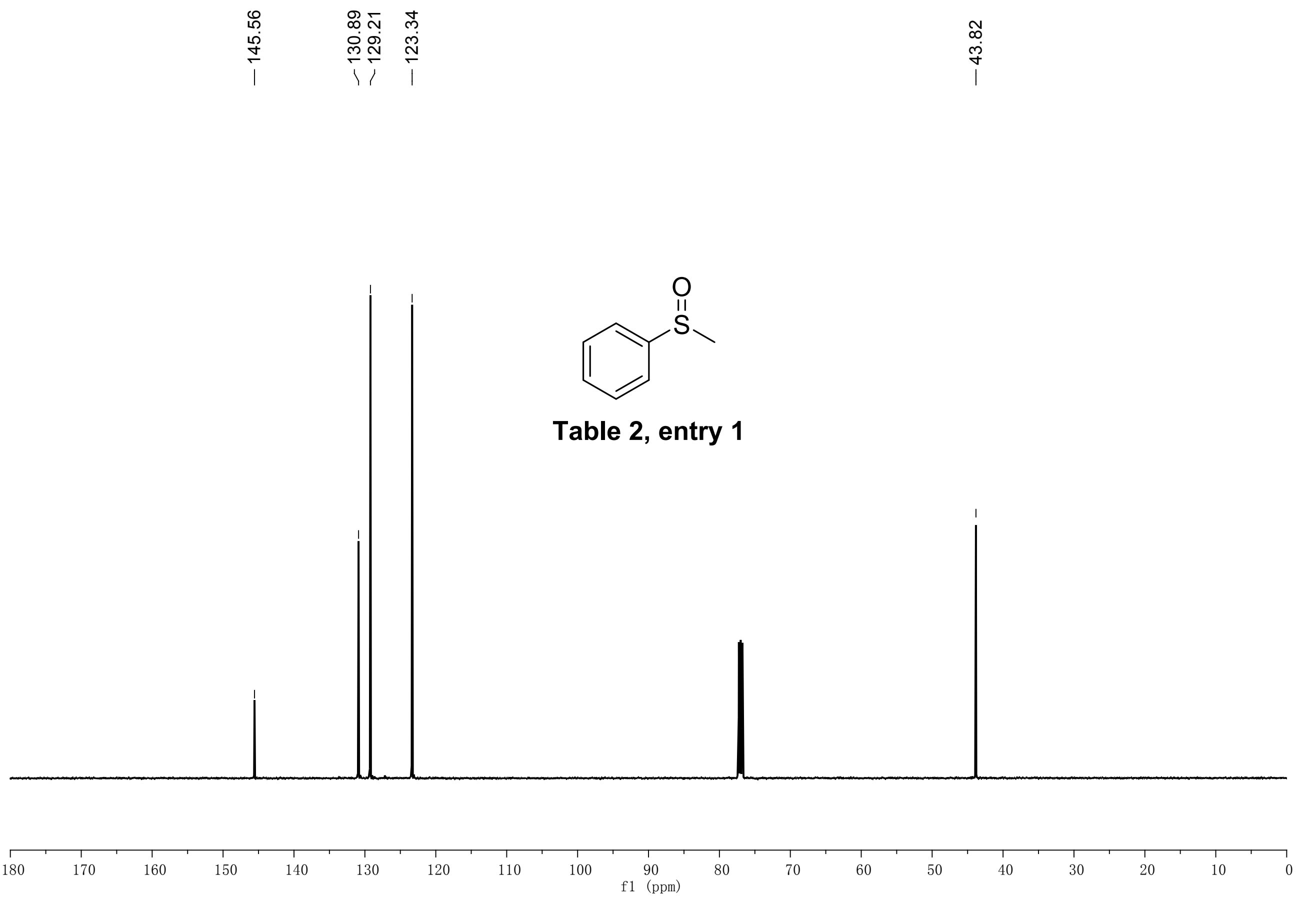

^{1}H NMR and ^{13}C NMR of **2b.**

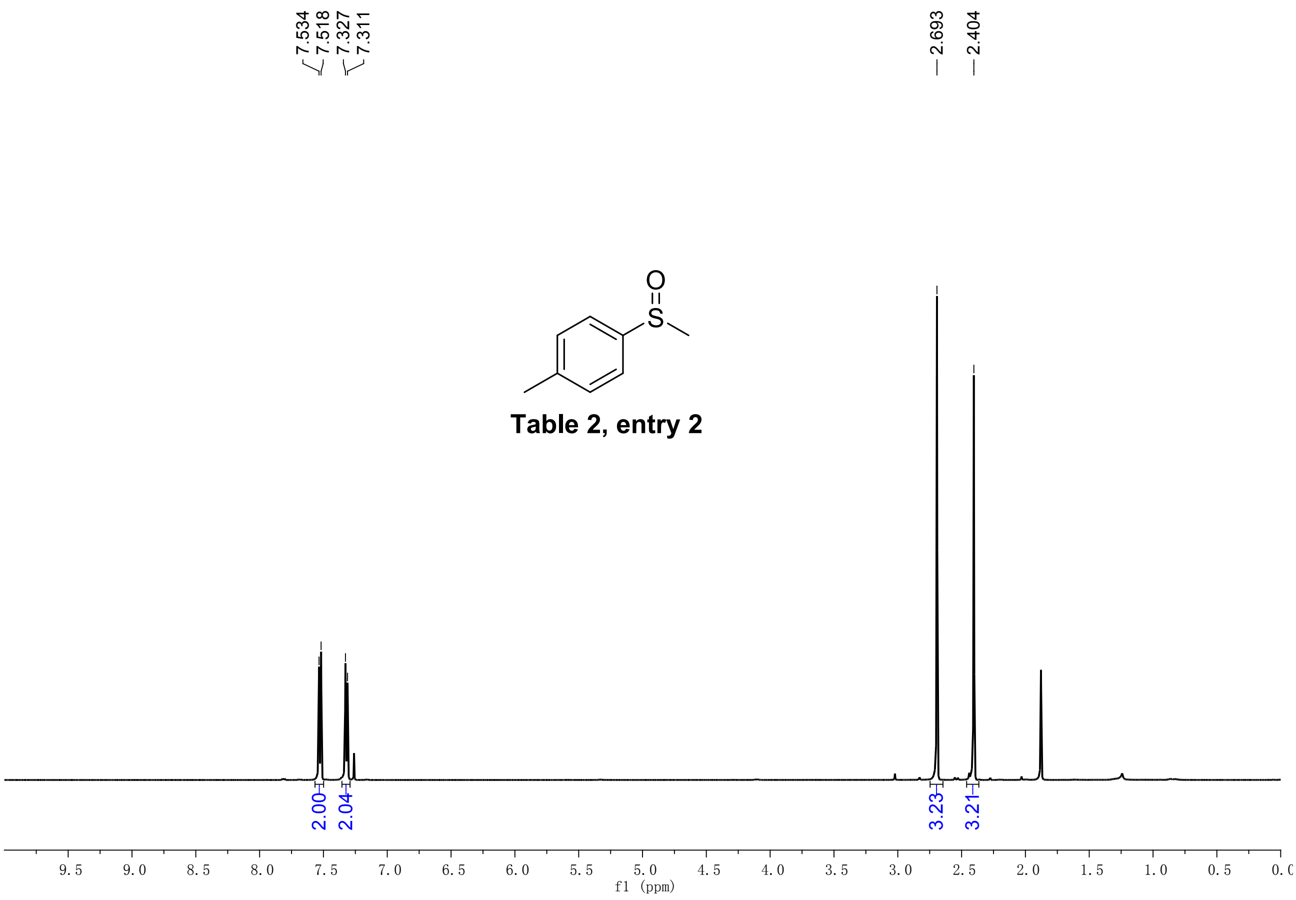

Table 2, entry 2

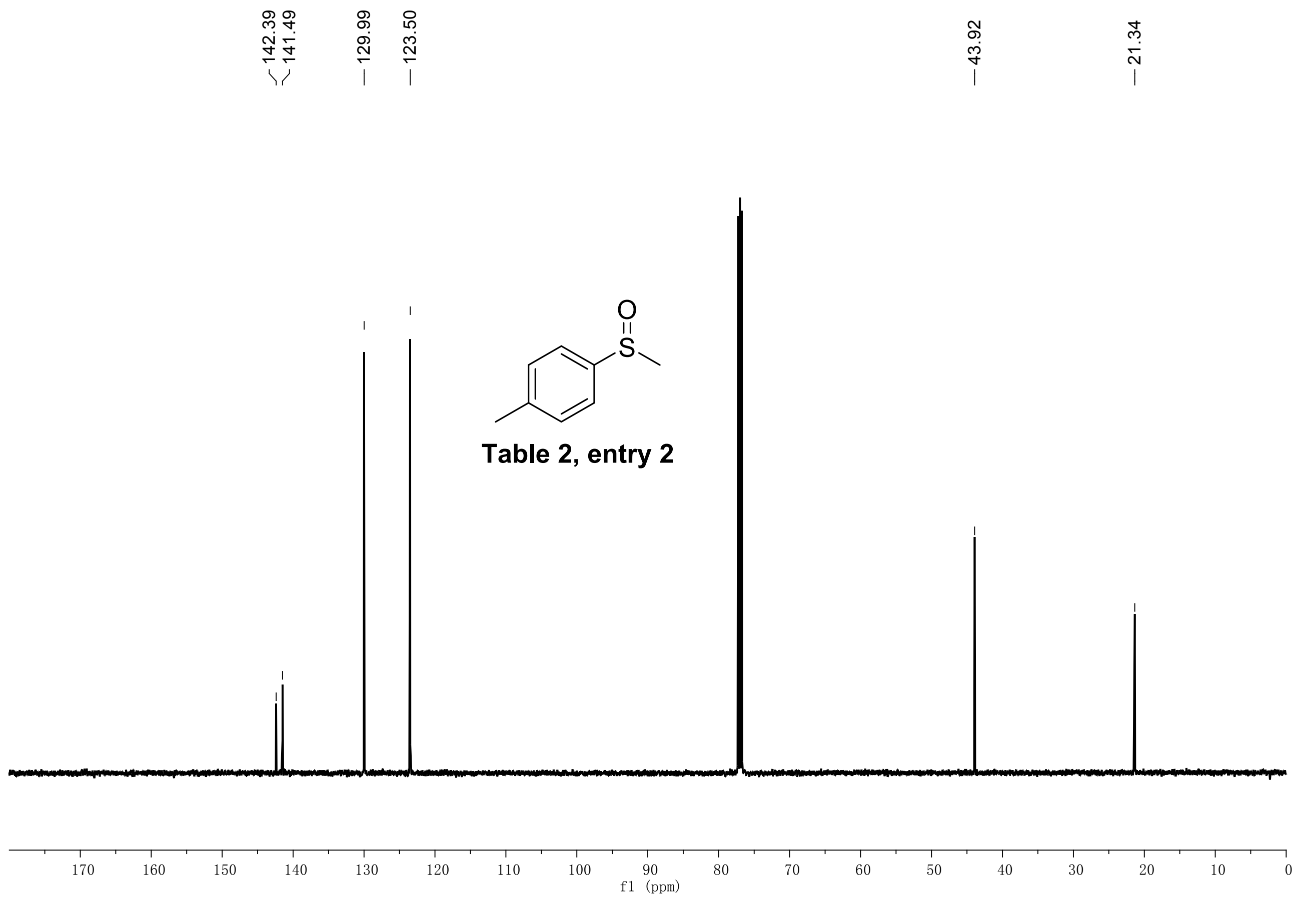

^{1}H NMR and ^{13}C NMR of **2c.**

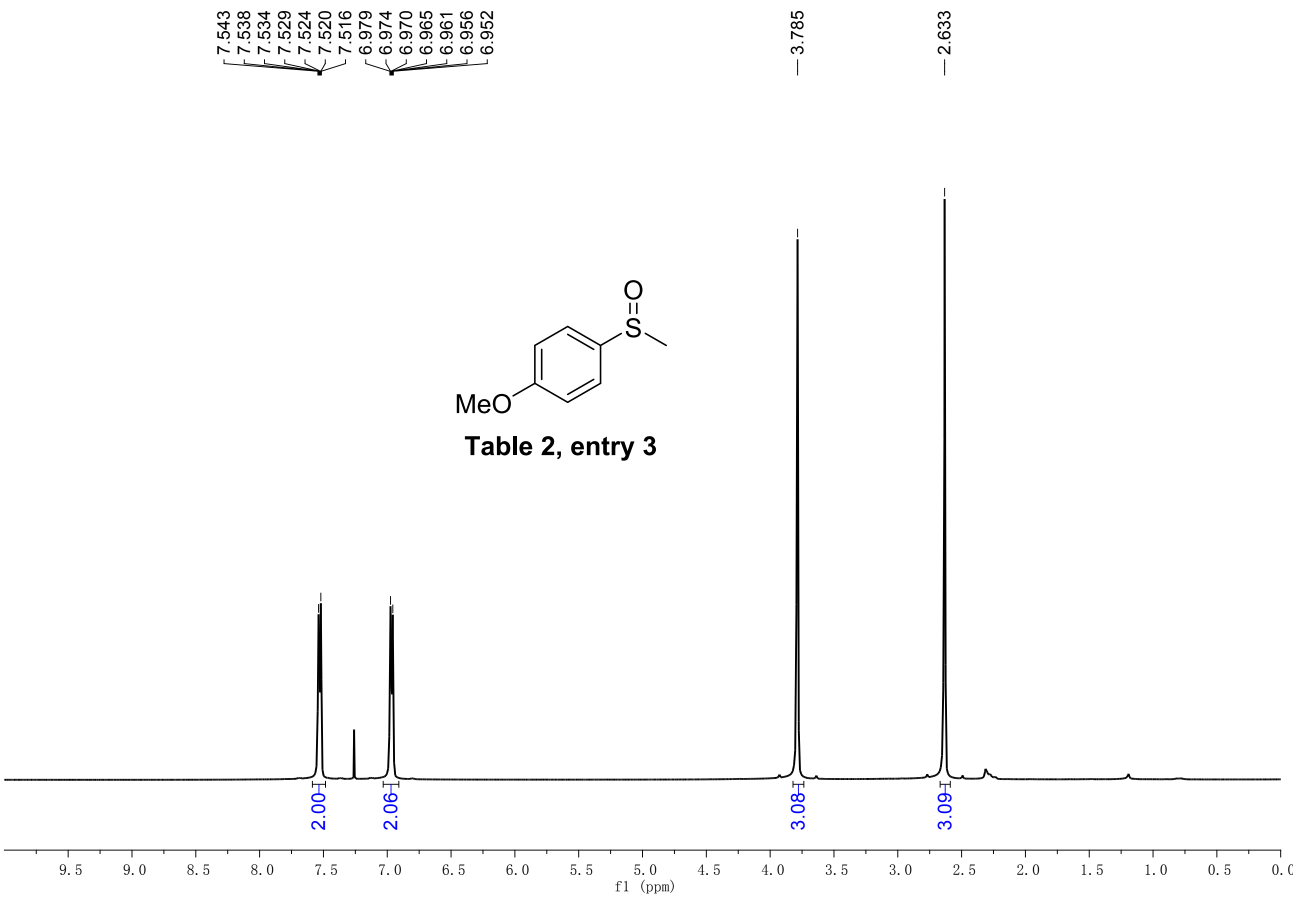

Table 2, entry 3

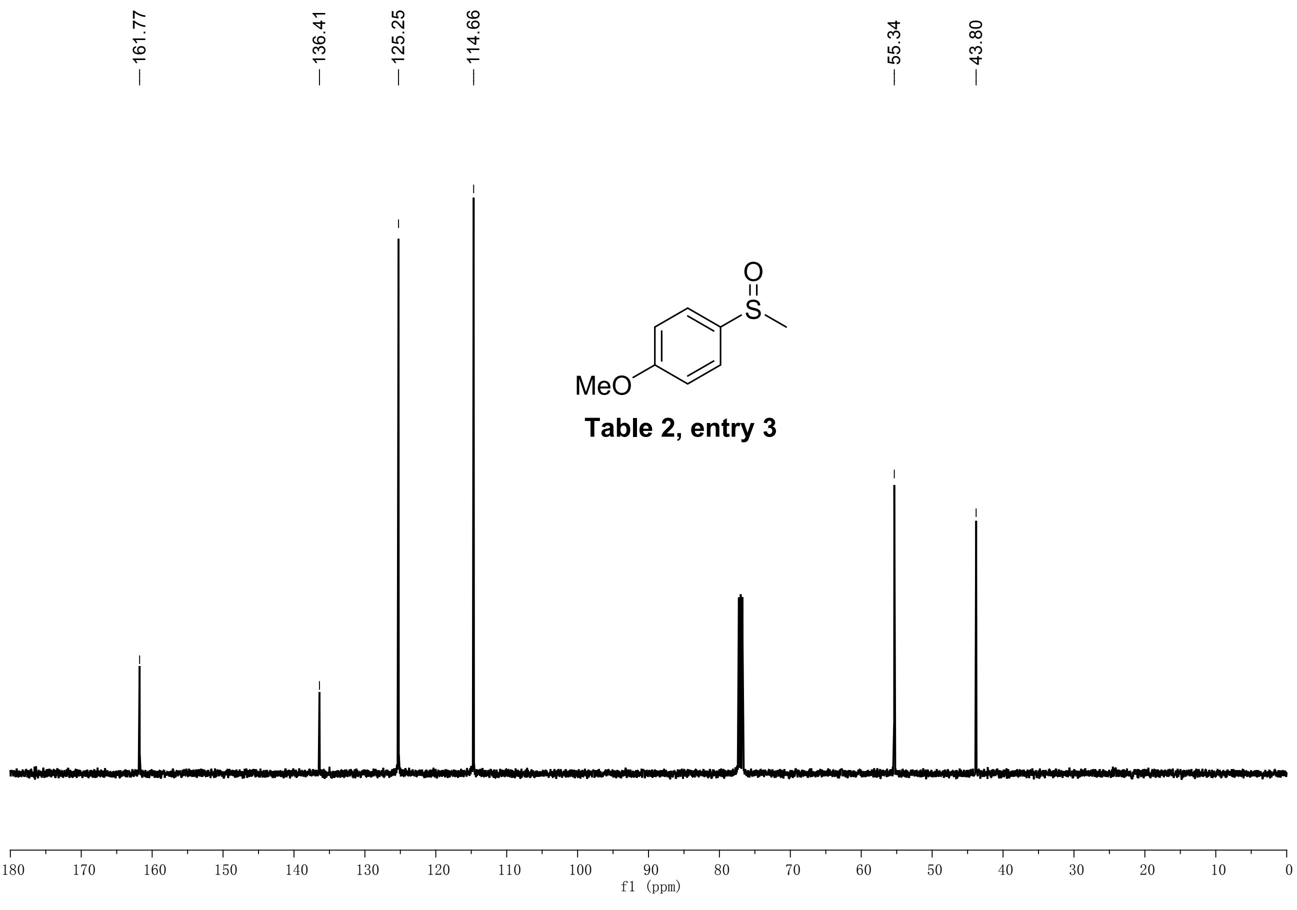

^{1}H NMR and ^{13}C NMR of **2d.**

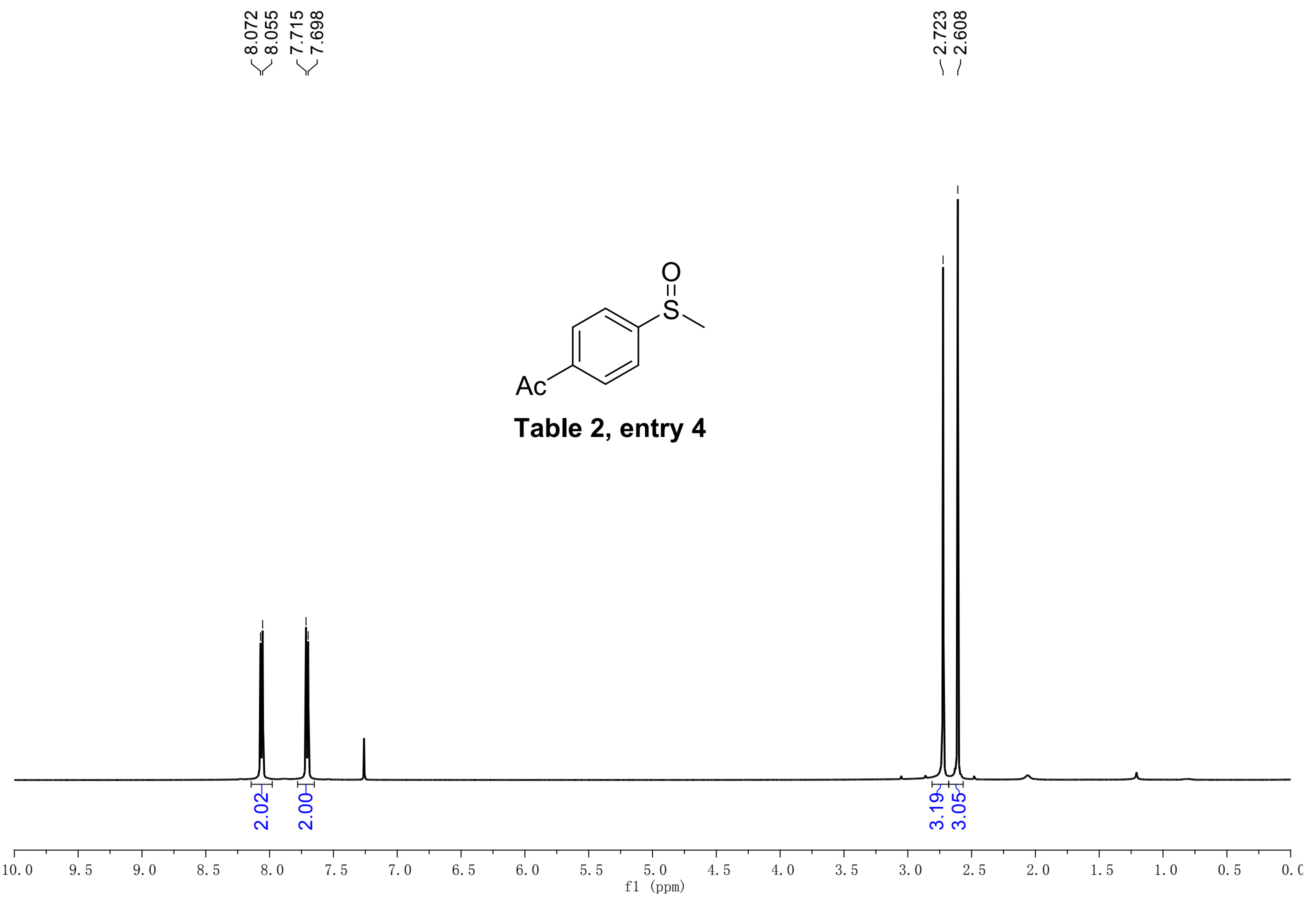
8.072
8.055
7.715
7.698
2.723
2.608
O
S
Ac
Table 2, entry 4
2.02
2.00
3.19
3.05
10.0 9.5 9.0 8.5 8.0 7.5 7.0 6.5 6.0 5.5 5.0 4.5 4.0 3.5 3.0 2.5 2.0 1.5 1.0 0.5 0.0
f1 (ppm)

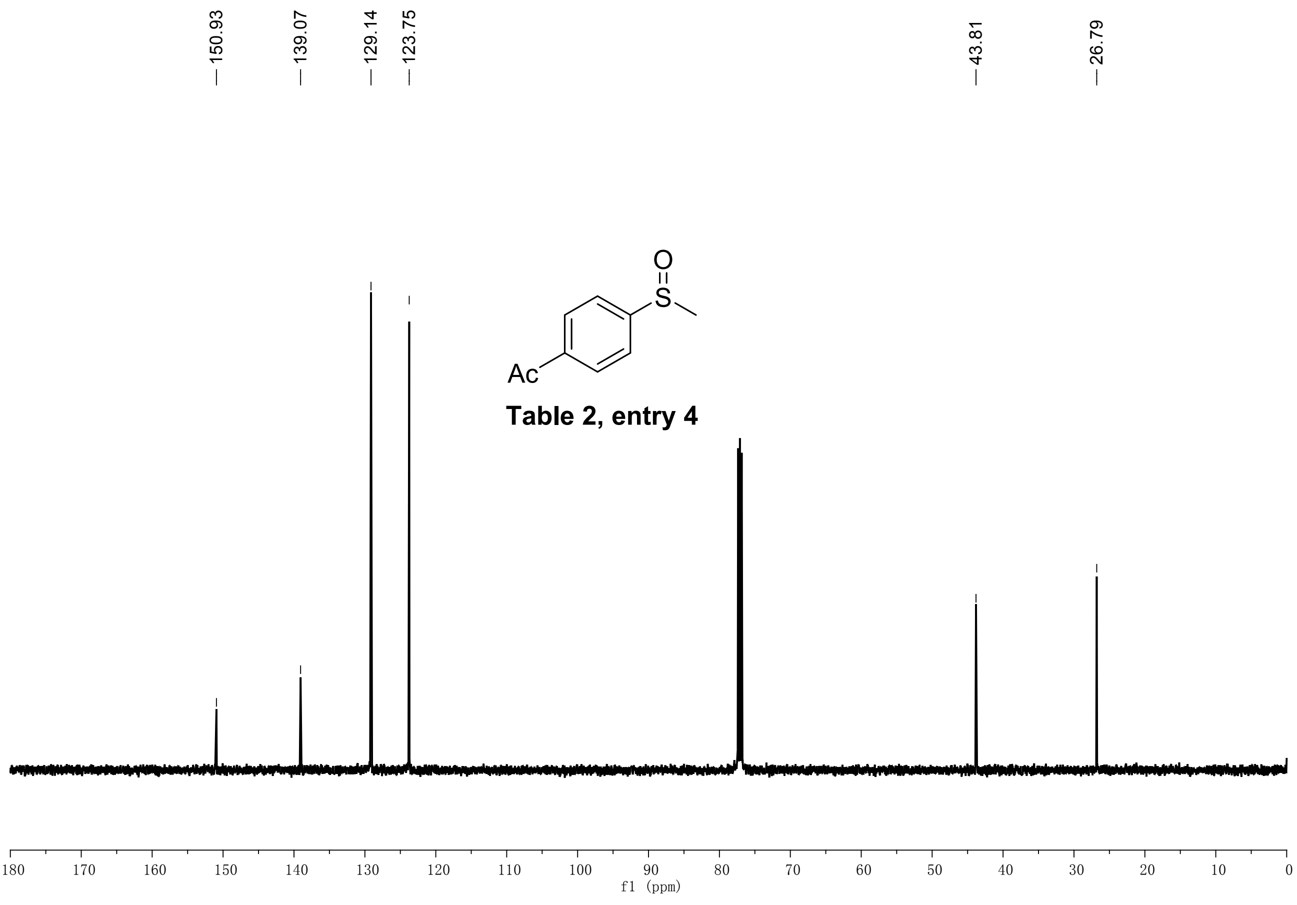

^{1}H NMR and ^{13}C NMR of **2e.**

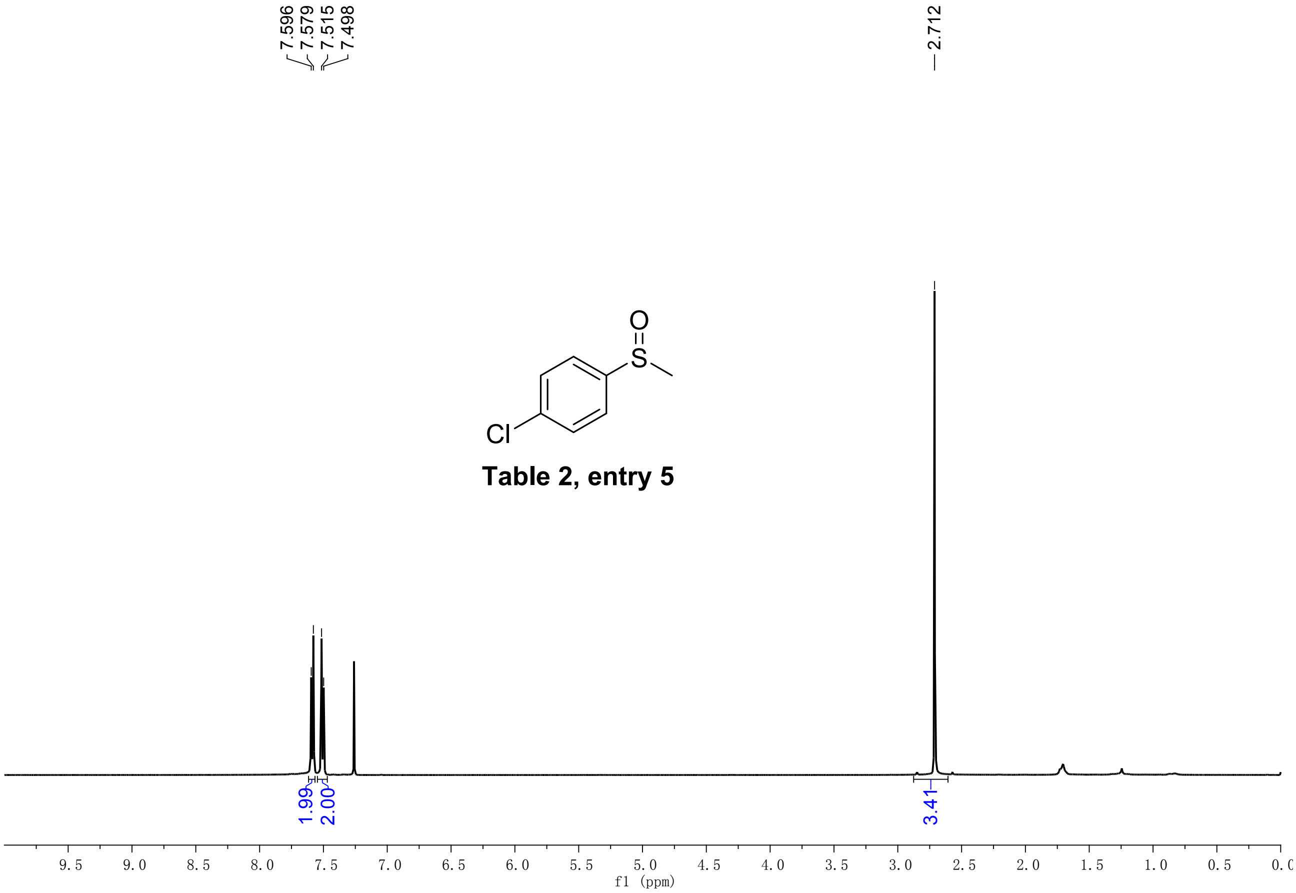

Table 2, entry 5

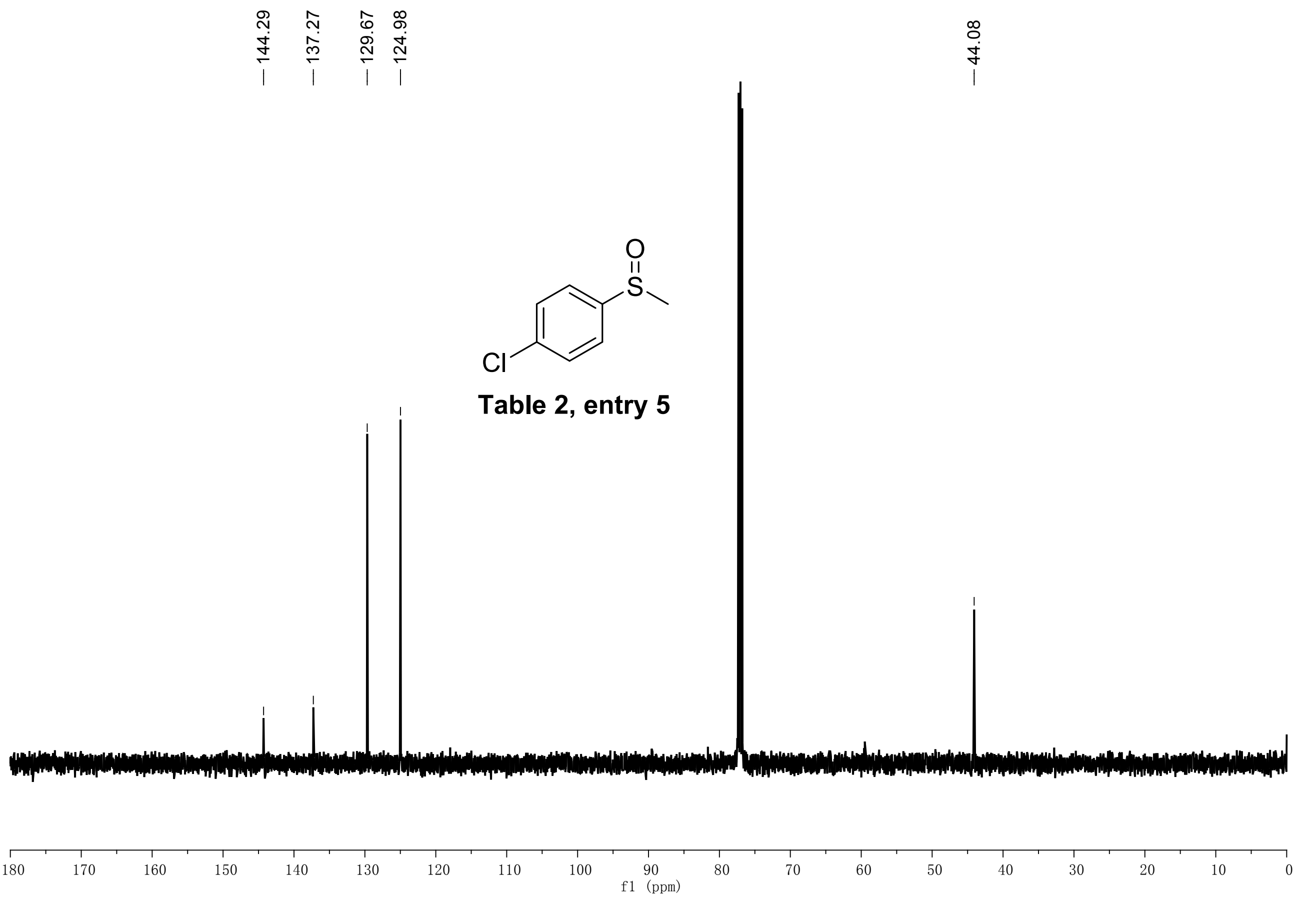

^{1}H NMR and ^{13}C NMR of **2f.**

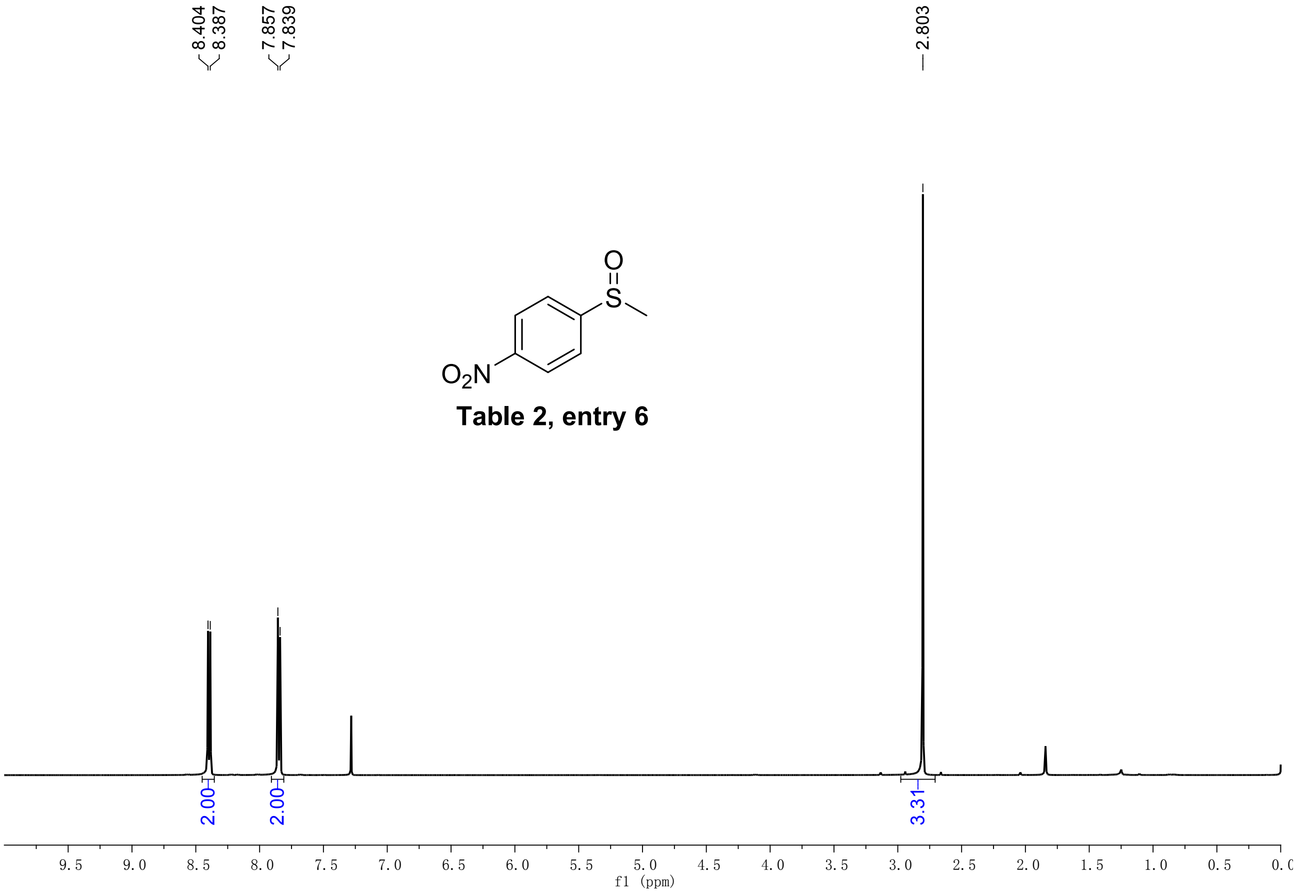

Table 2, entry 6

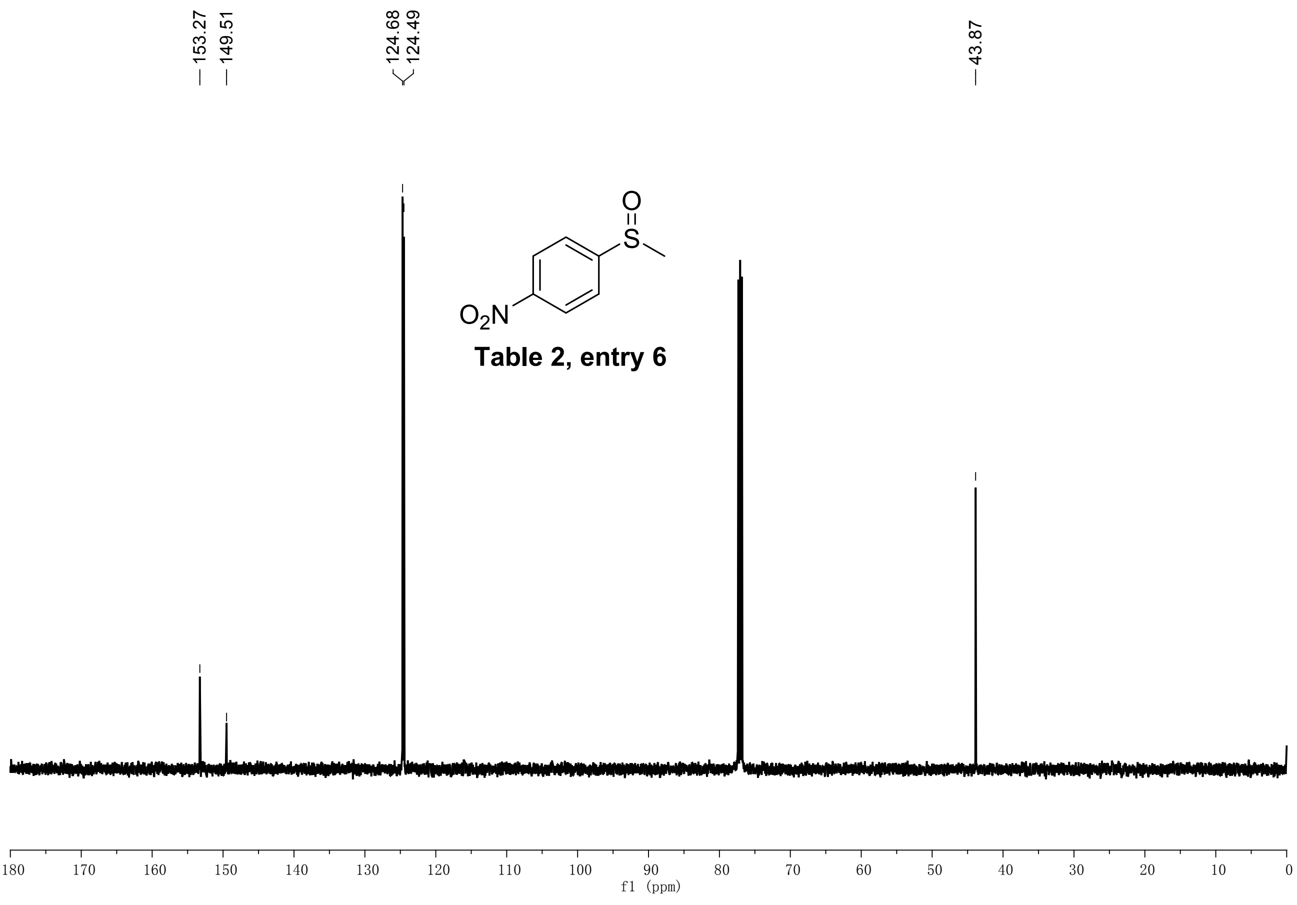

^{1}H NMR and ^{13}C NMR of **2g.**

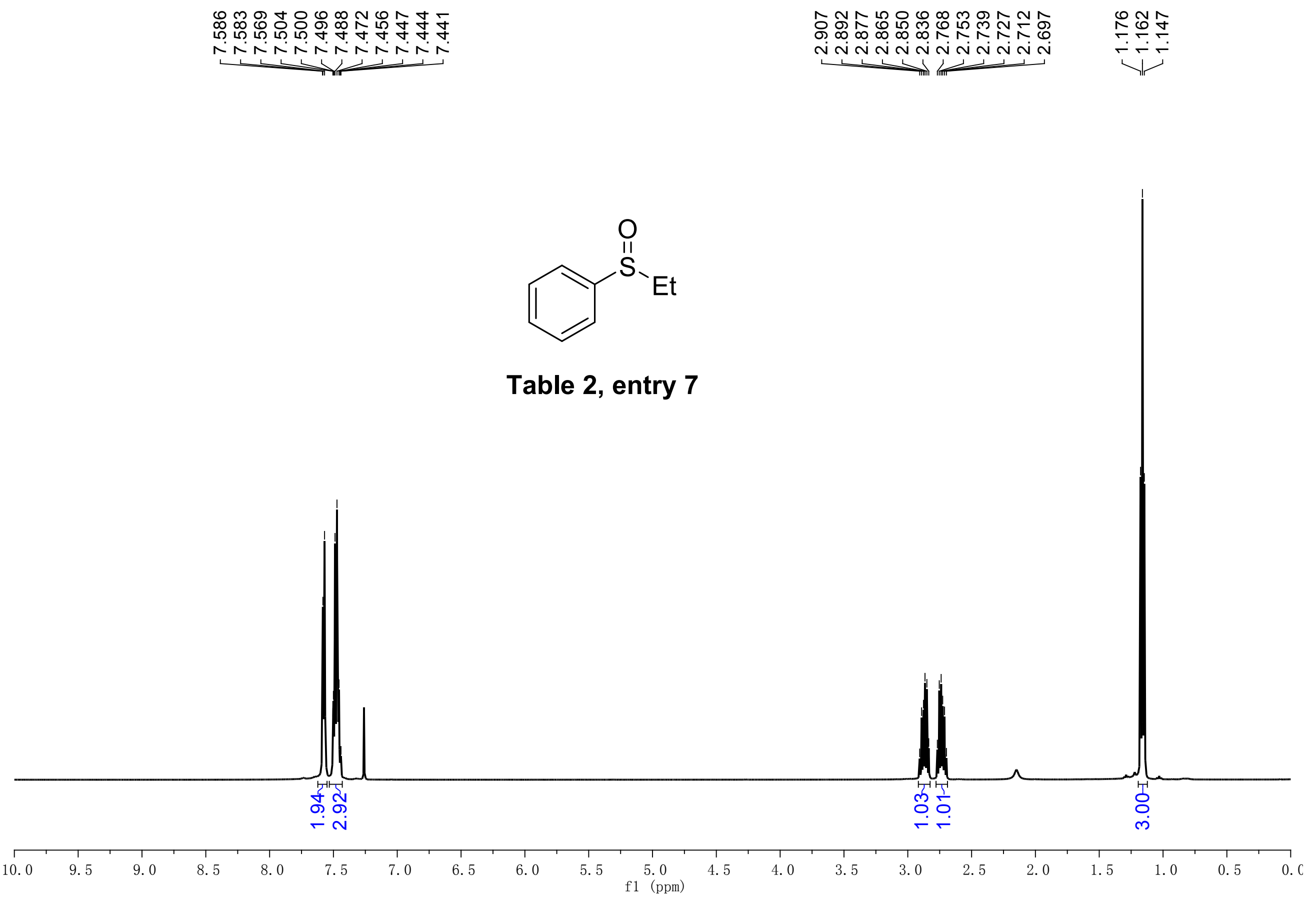
7.586
7.583
7.569
7.504
7.500
7.496
7.488
7.472
7.456
7.447
7.444
7.441
2.907
2.892
2.877
2.865
2.850
2.836
2.768
2.753
2.739
2.727
2.712
2.697
1.176
1.162
1.147
O
S
Et
Table 2, entry 7
1.94
2.92
1.03
1.01
3.00
10.0 9.5 9.0 8.5 8.0 7.5 7.0 6.5 6.0 5.5 5.0 4.5 4.0 3.5 3.0 2.5 2.0 1.5 1.0 0.5
f1 (ppm)

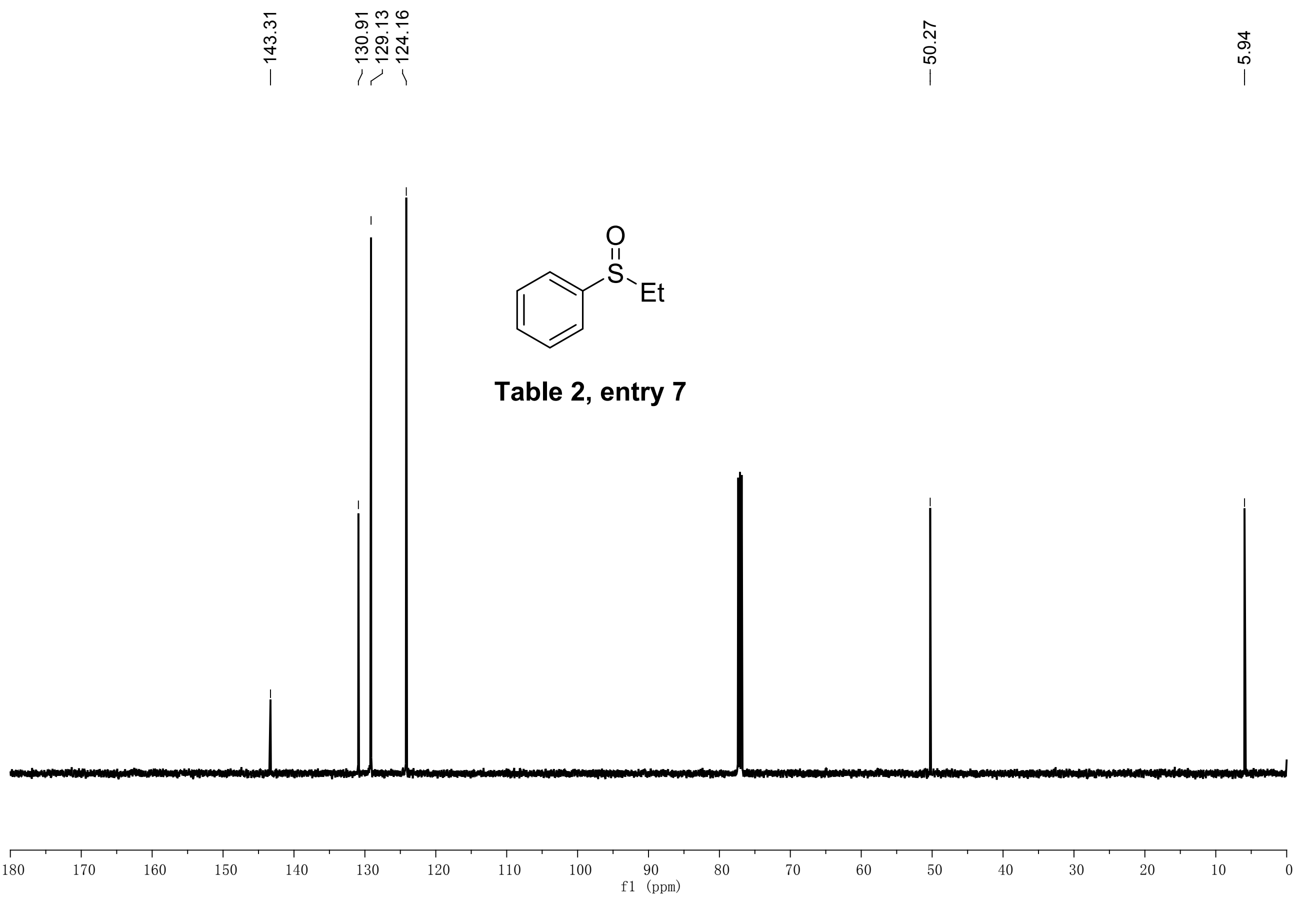

^{1}H NMR and ^{13}C NMR of **2h.**

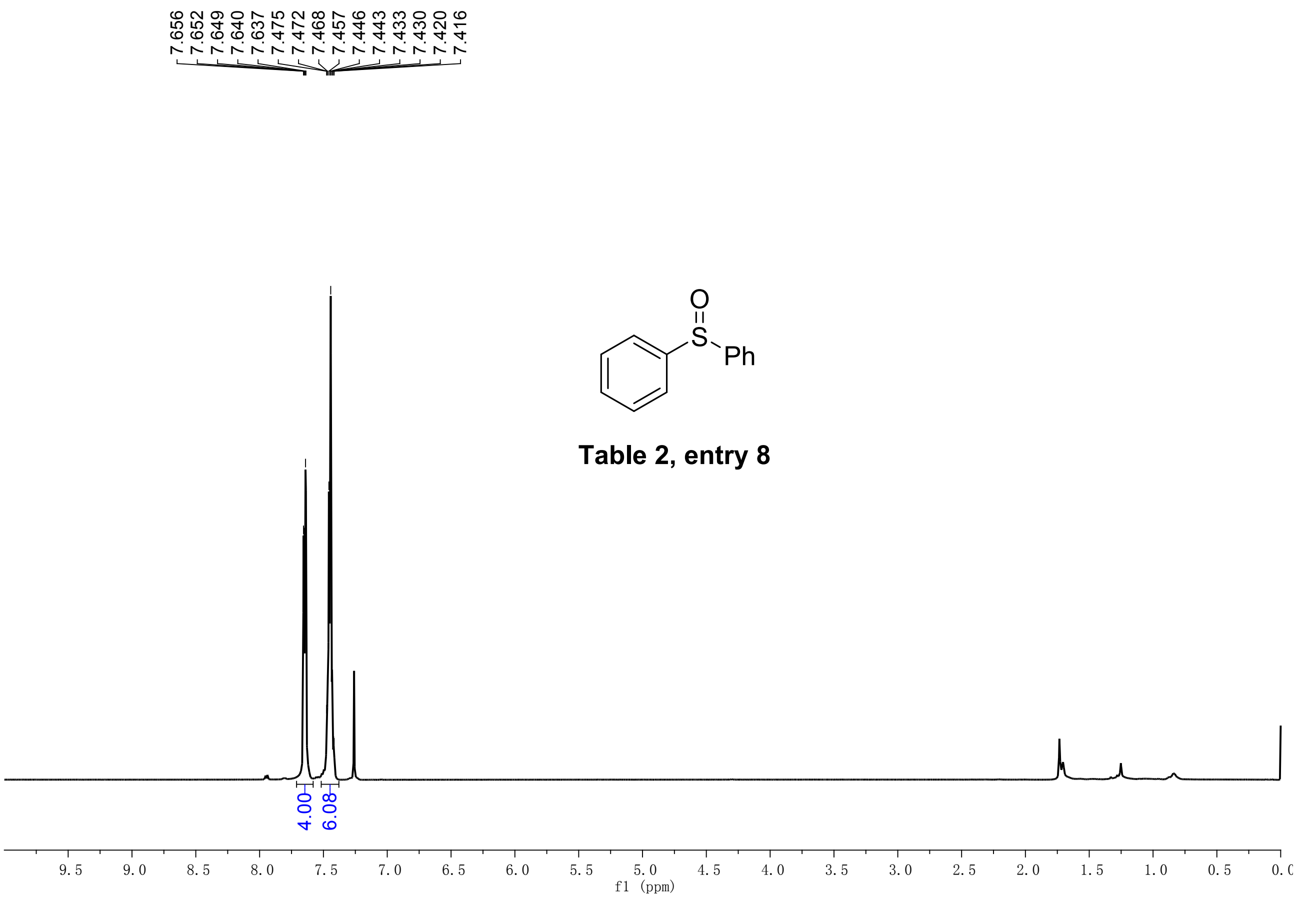

Table 2, entry 8

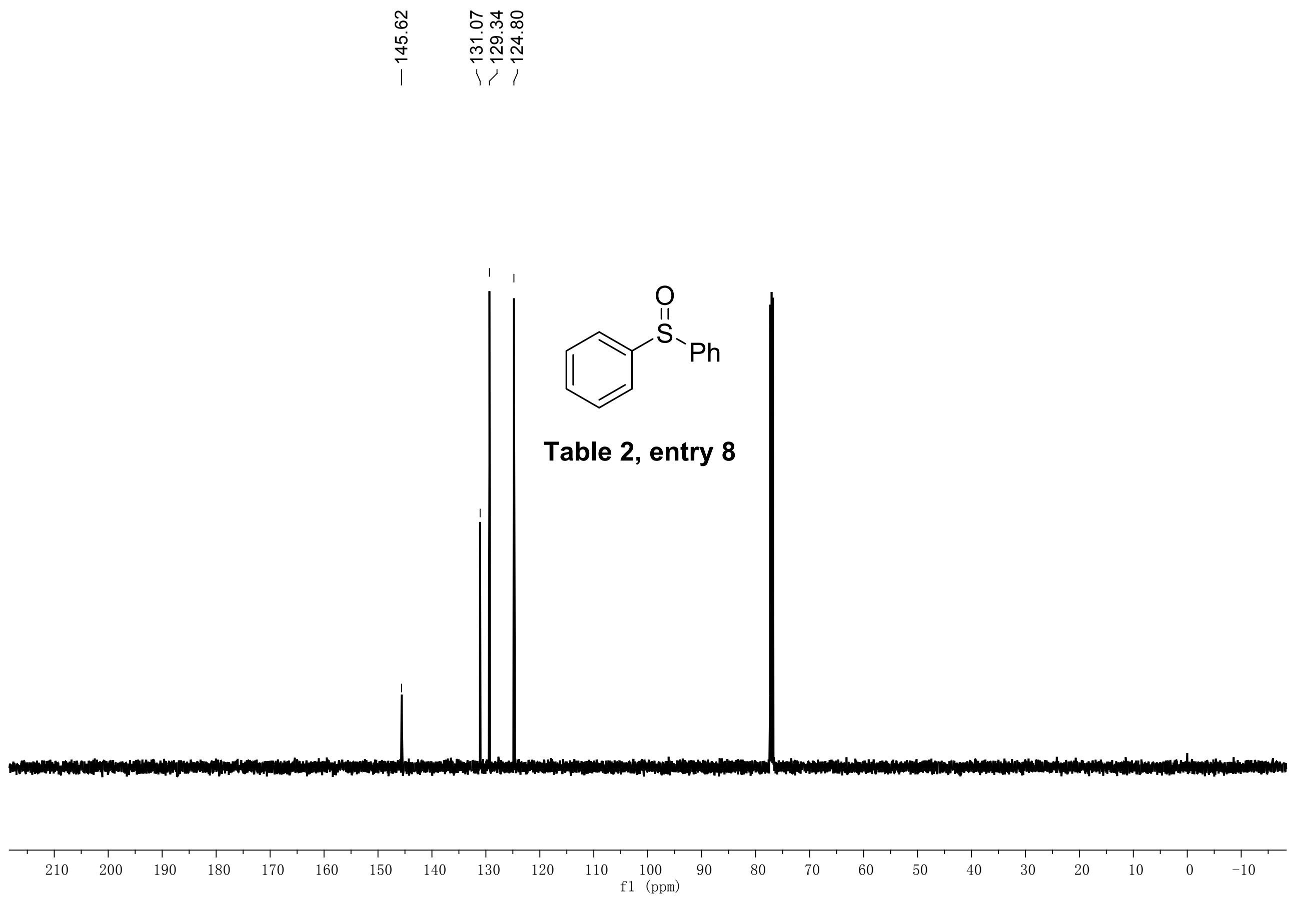

^{1}H NMR and ^{13}C NMR of **2i.**

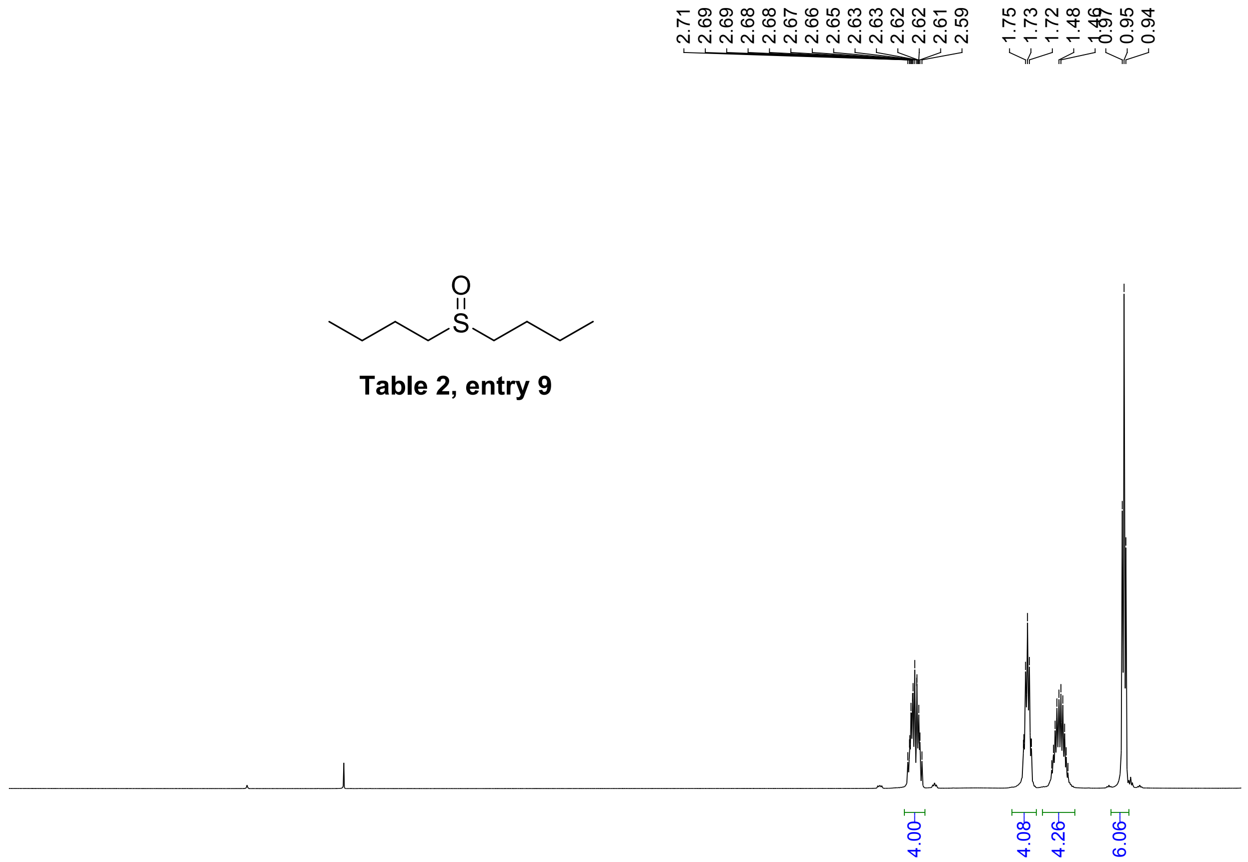

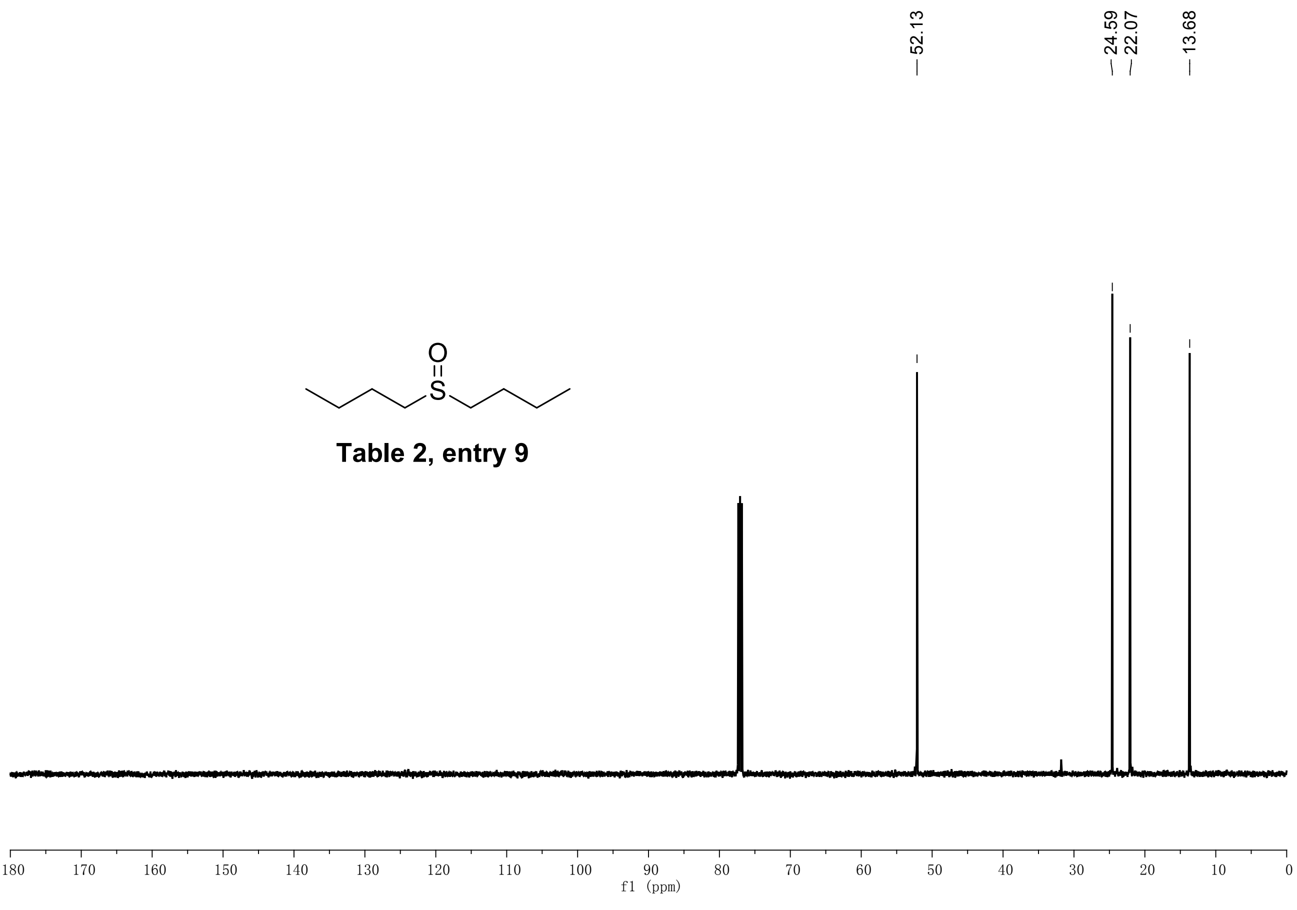

^{1}H NMR and ^{13}C NMR of **3a.**

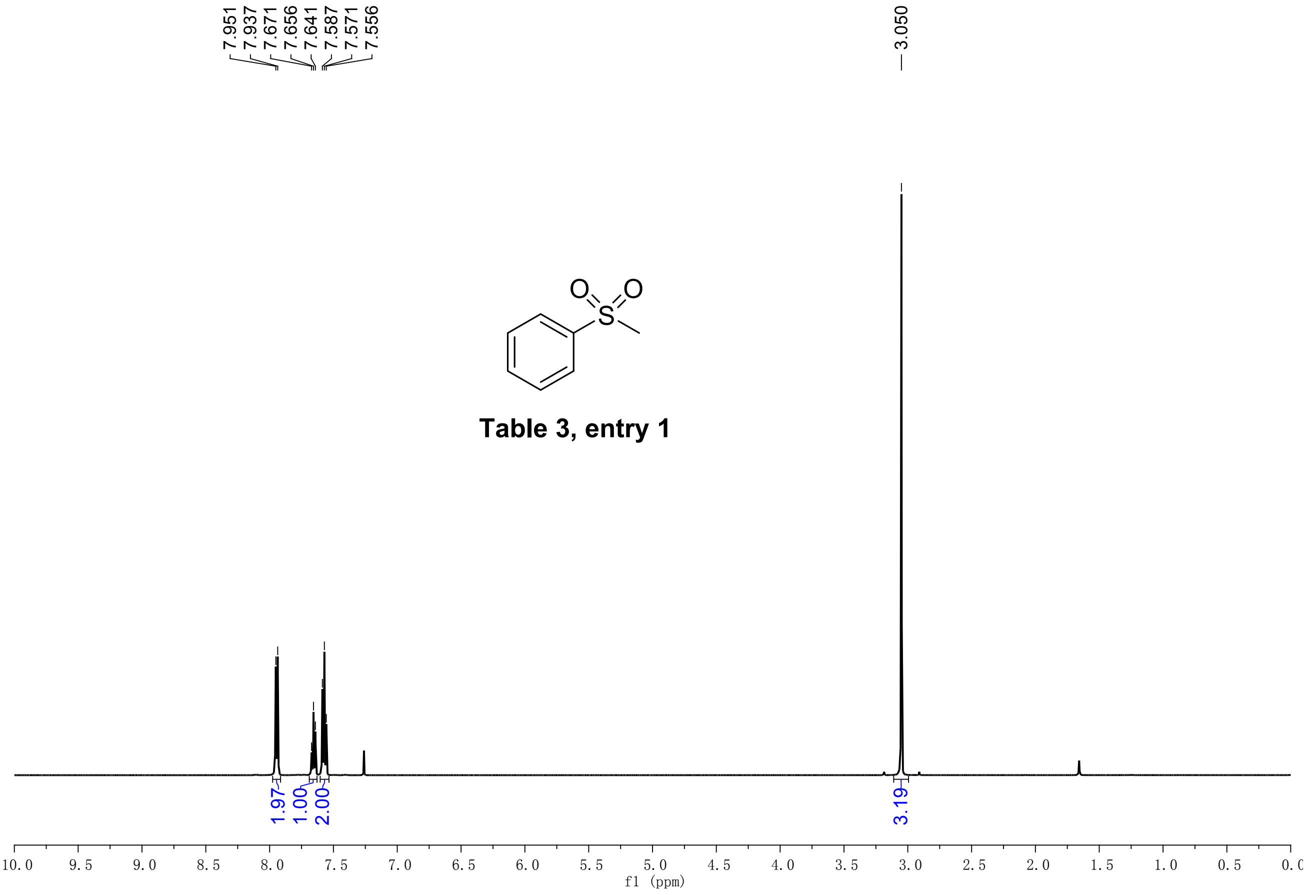
7.951
7.937
7.671
7.656
7.641
7.587
7.571
7.556
3.050
Table 3, entry 1
1.97
1.00
2.00
3.19
10.0 9.5 9.0 8.5 8.0 7.5 7.0 6.5 6.0 5.5 5.0 4.5 4.0 3.5 3.0 2.5 2.0 1.5 1.0 0.5 0.0
f1 (ppm)

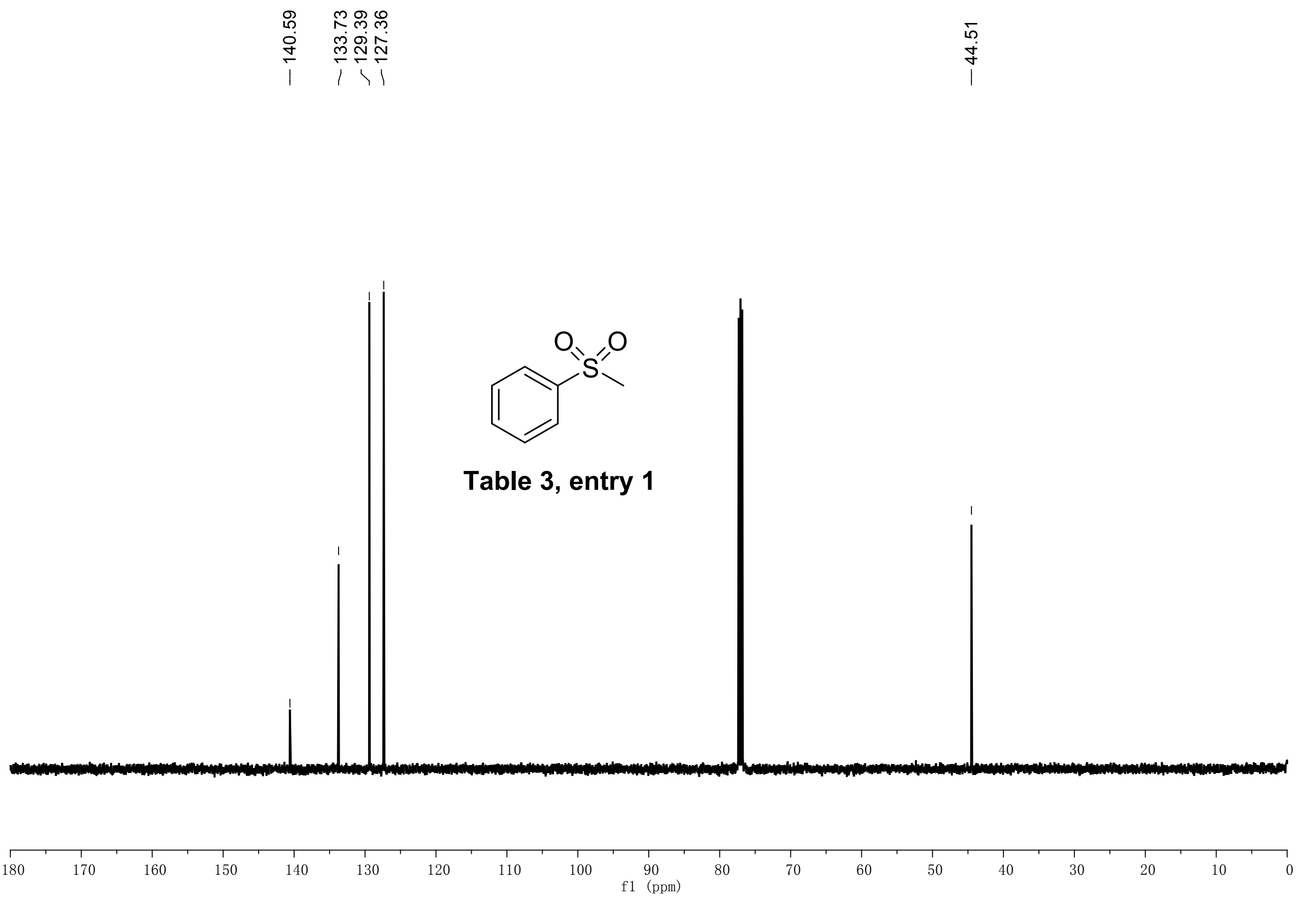

^{1}H NMR and ^{13}C NMR of **3b.**

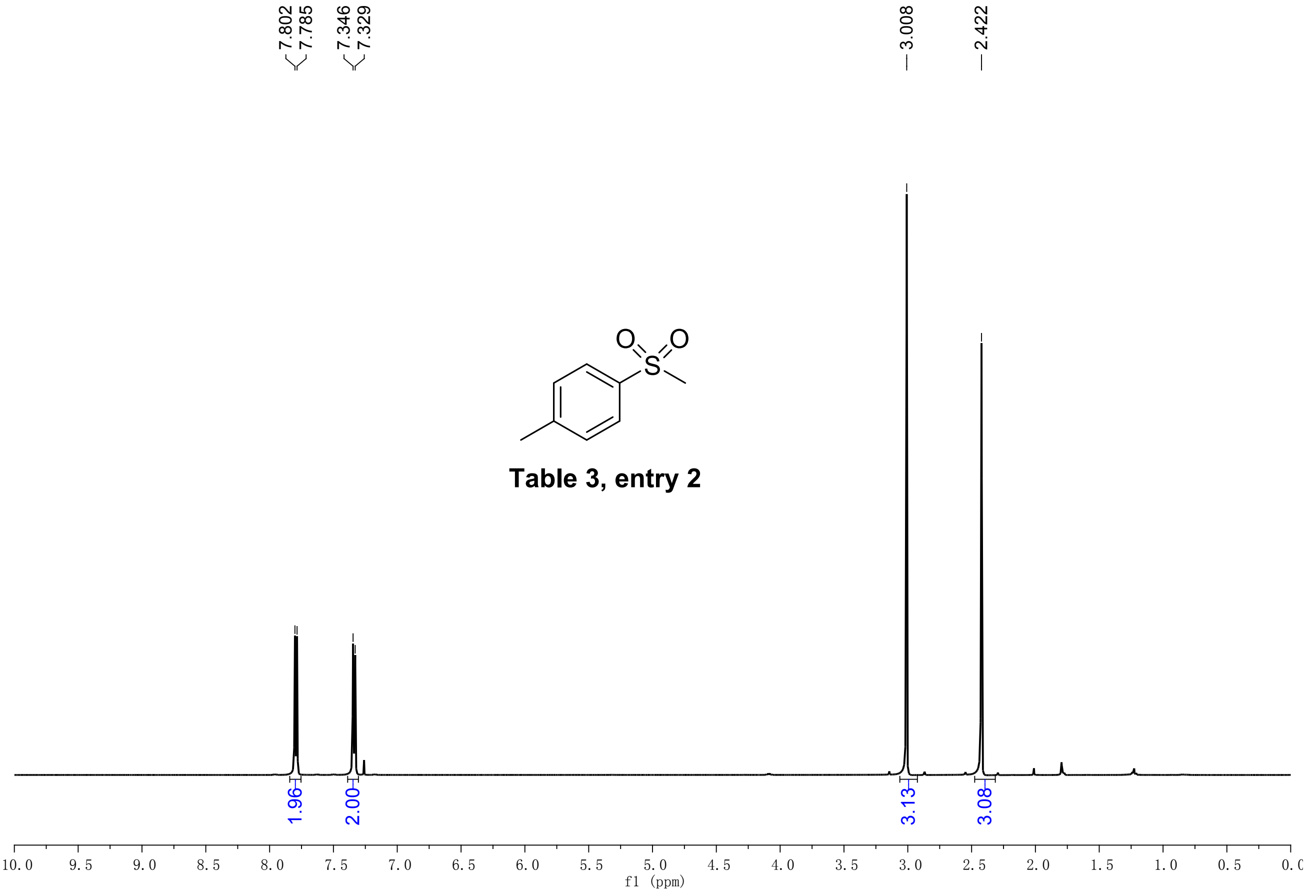

7.802
7.785
7.346
7.329
3.008
2.422
Table 3, entry 2
1.96
2.00
3.13
3.08
10.0 9.5 9.0 8.5 8.0 7.5 7.0 6.5 6.0 5.5 5.0 4.5 4.0 3.5 3.0 2.5 2.0 1.5 1.0 0.5 0.0
f1 (ppm)

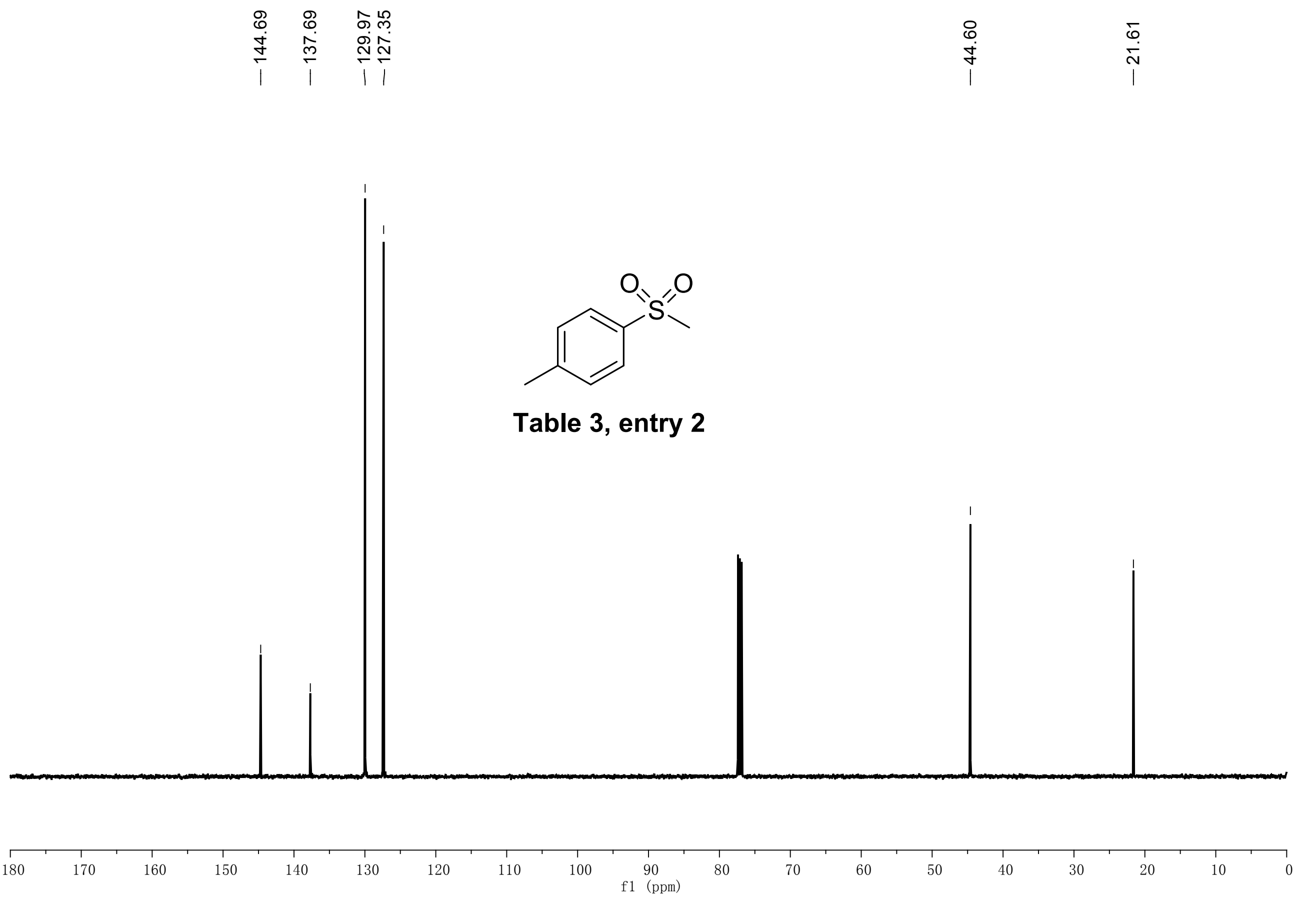

^{1}H NMR and ^{13}C NMR of **3c.**

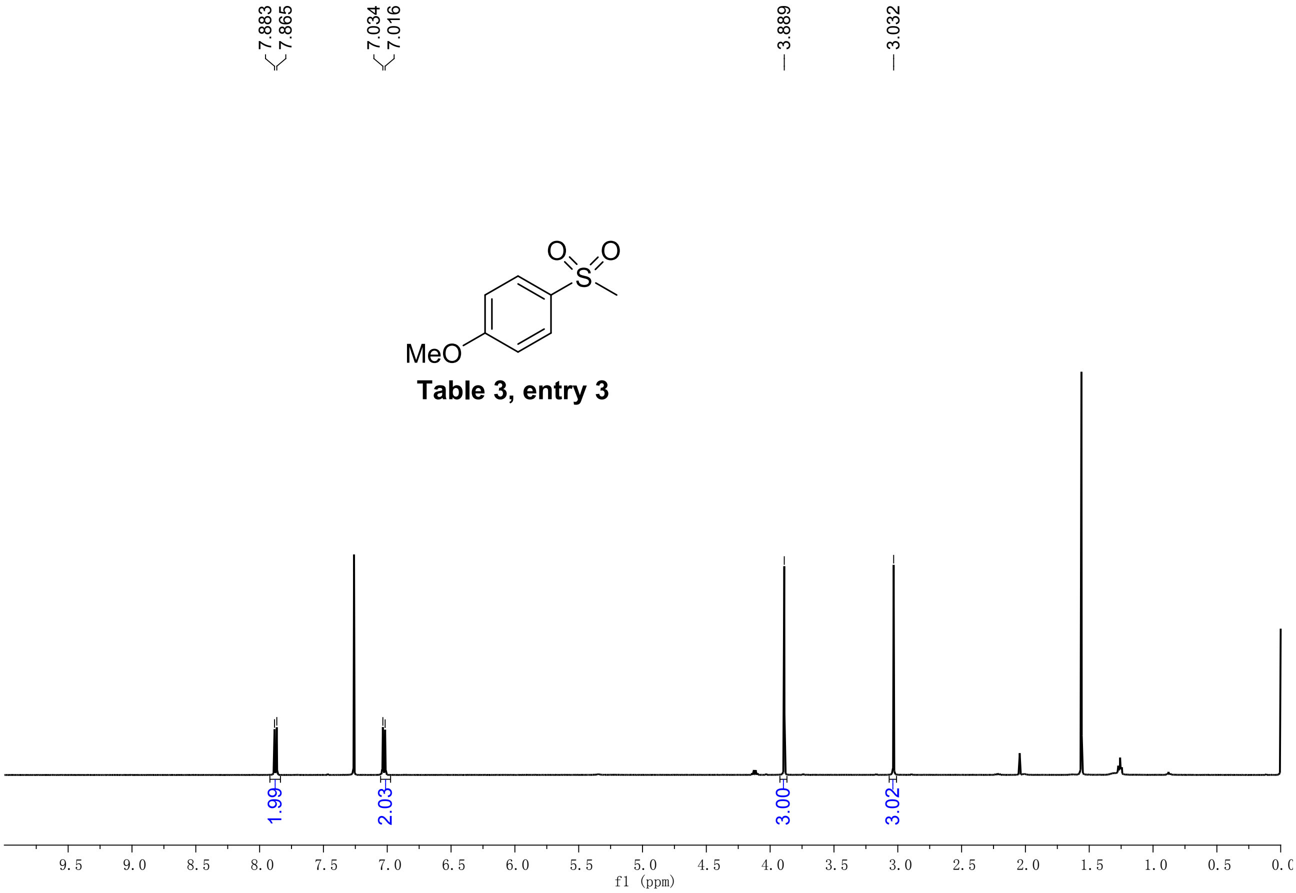
7.883
7.865
7.034
7.016
3.889
3.032
MeO
O
S
O
Table 3, entry 3
1.99
2.03
3.00
3.02
9.5
9.0
8.5
8.0
7.5
7.0
6.5
6.0
5.5
5.0
4.5
4.0
3.5
3.0
2.5
2.0
1.5
1.0
0.5
f1 (ppm)

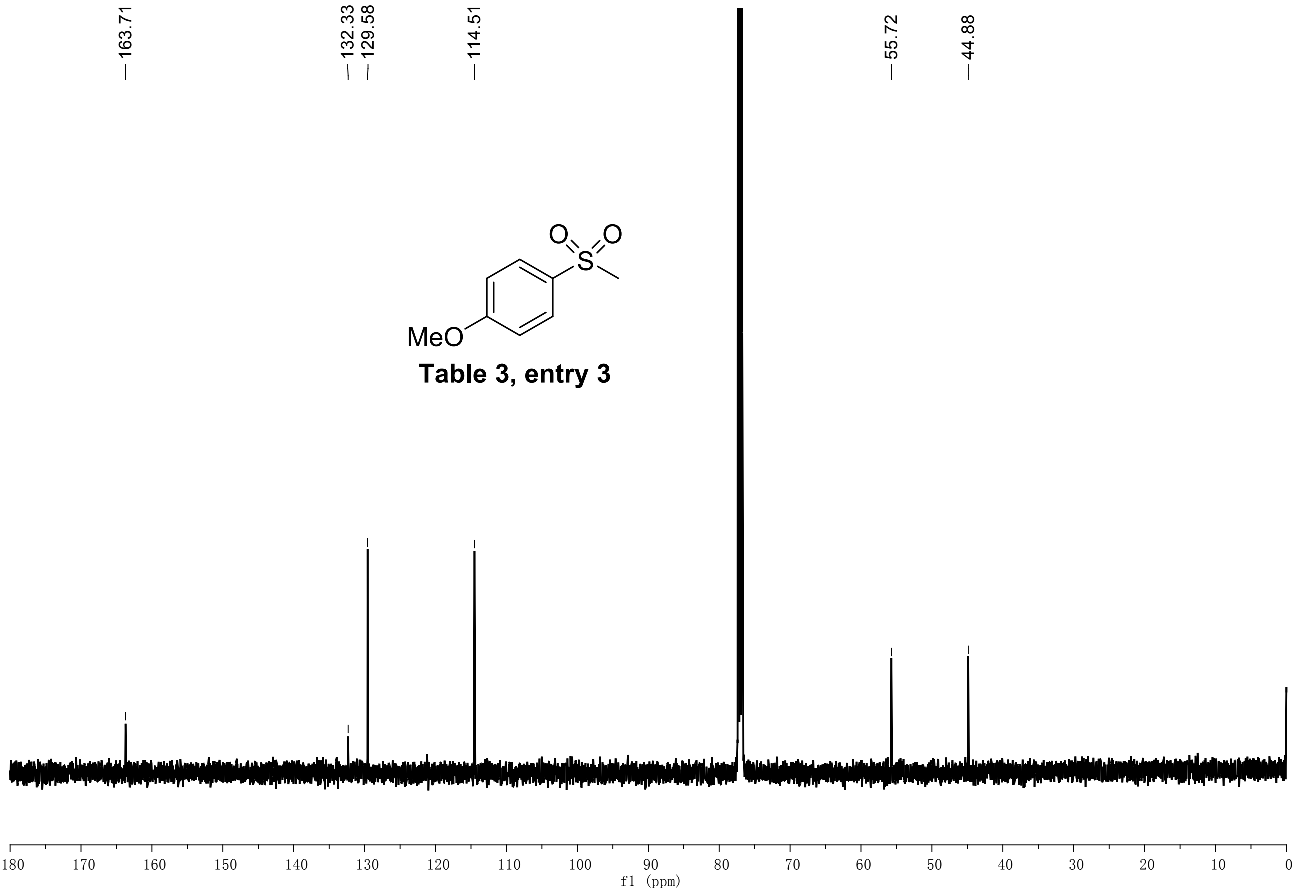

^1H NMR and ^13C NMR of **3d.**

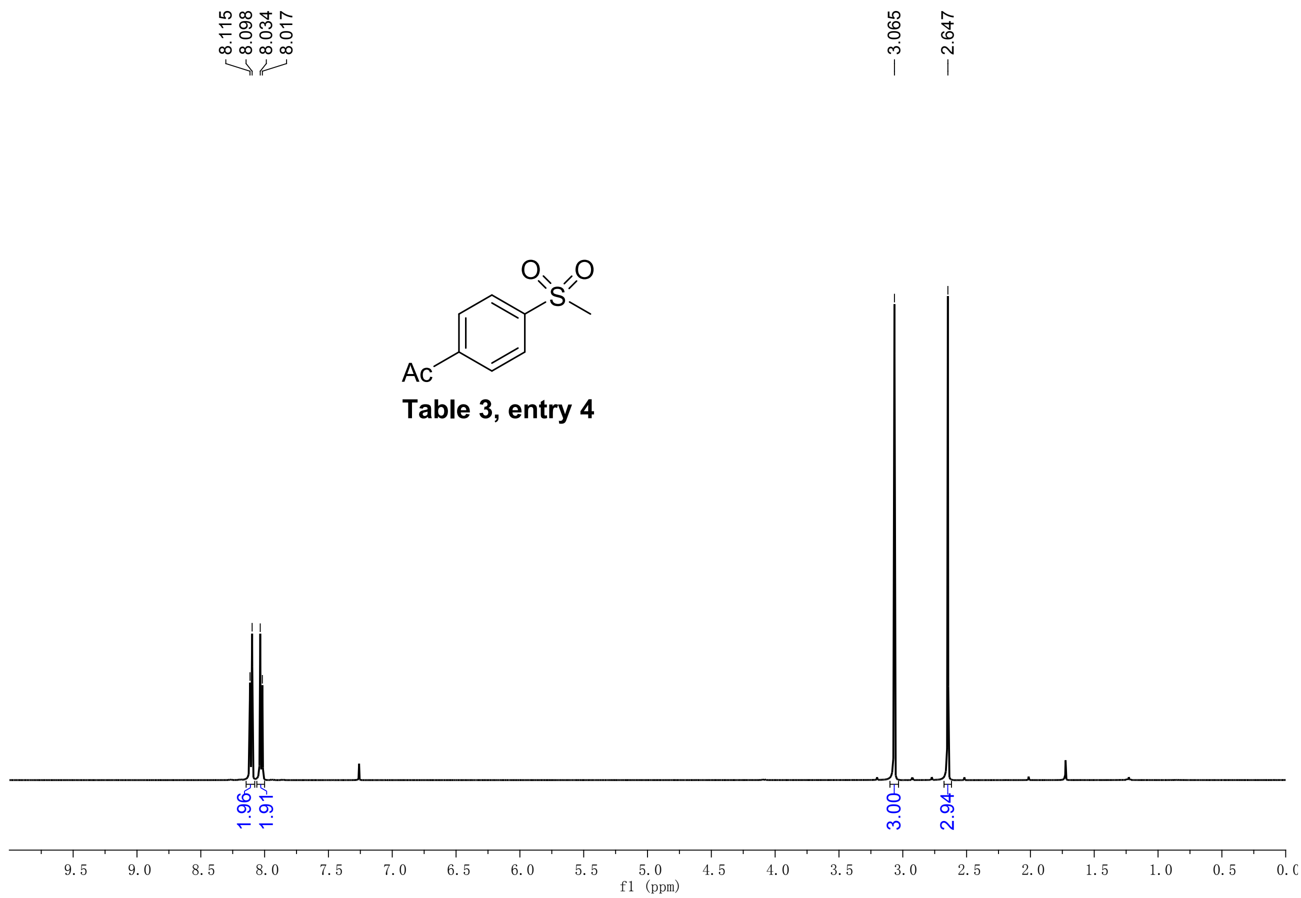
8.115
8.098
8.034
8.017
3.065
2.647
Ac
Table 3, entry 4
1.96
1.91
3.00
2.94
9.5 9.0 8.5 8.0 7.5 7.0 6.5 6.0 5.5 5.0 4.5 4.0 3.5 3.0 2.5 2.0 1.5 1.0 0.5 0.0
f1 (ppm)

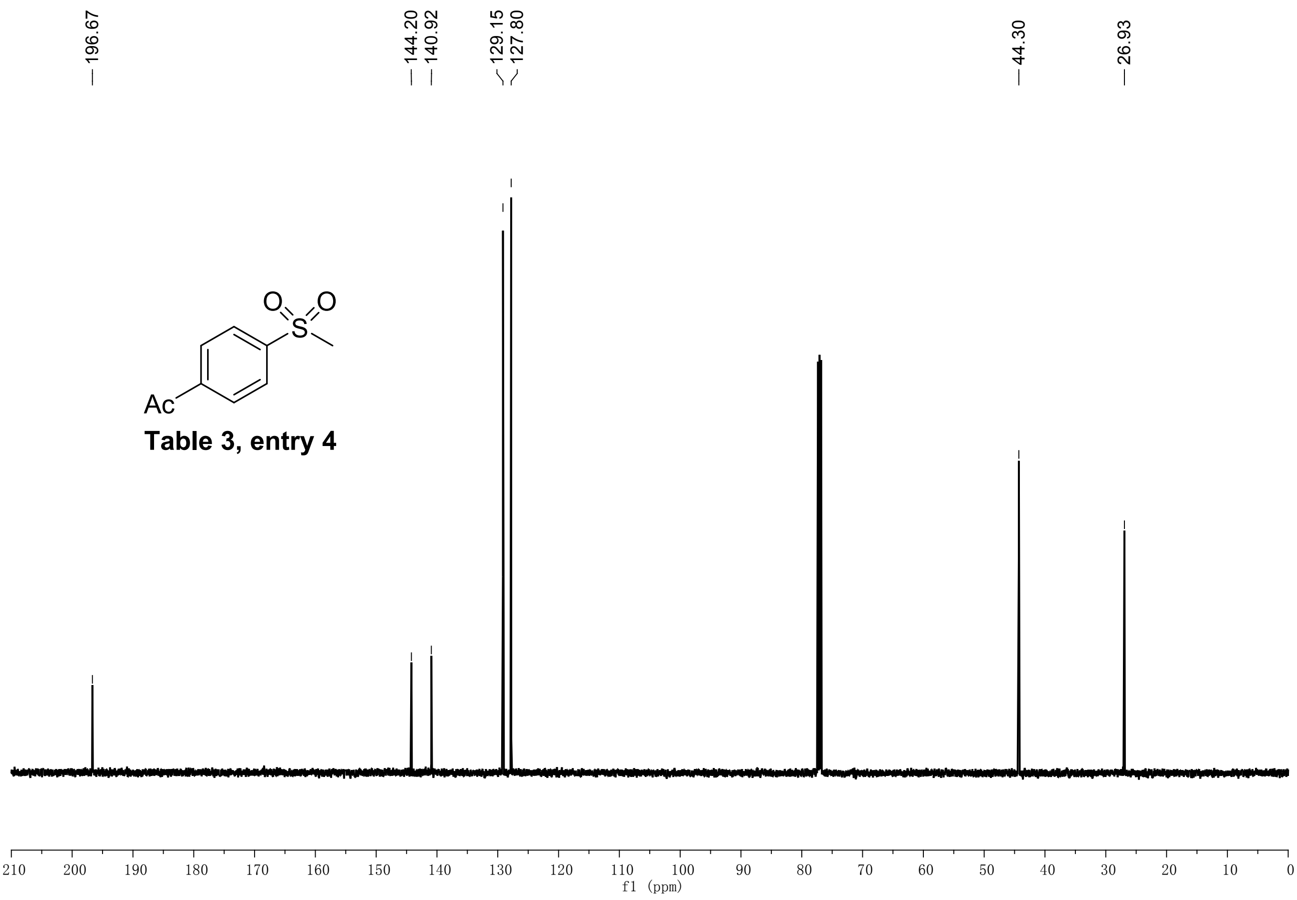

^{1}H NMR and ^{13}C NMR of **3e.**

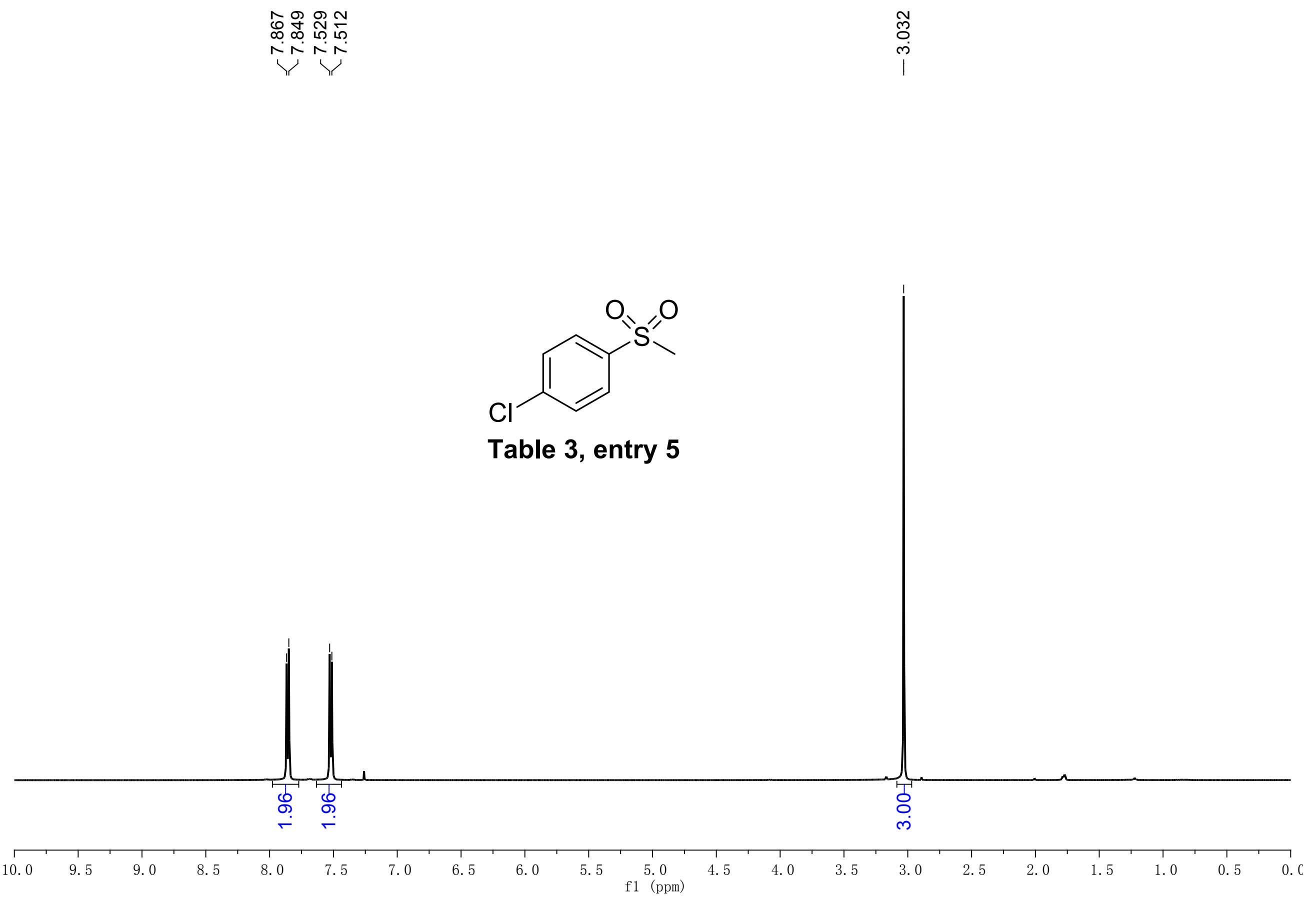
7.867
7.849
7.529
7.512
3.032
O
S
O
Cl
Table 3, entry 5
1.96
1.96
3.00
10.0 9.5 9.0 8.5 8.0 7.5 7.0 6.5 6.0 5.5 5.0 4.5 4.0 3.5 3.0 2.5 2.0 1.5 1.0 0.5 0.0
f1 (ppm)

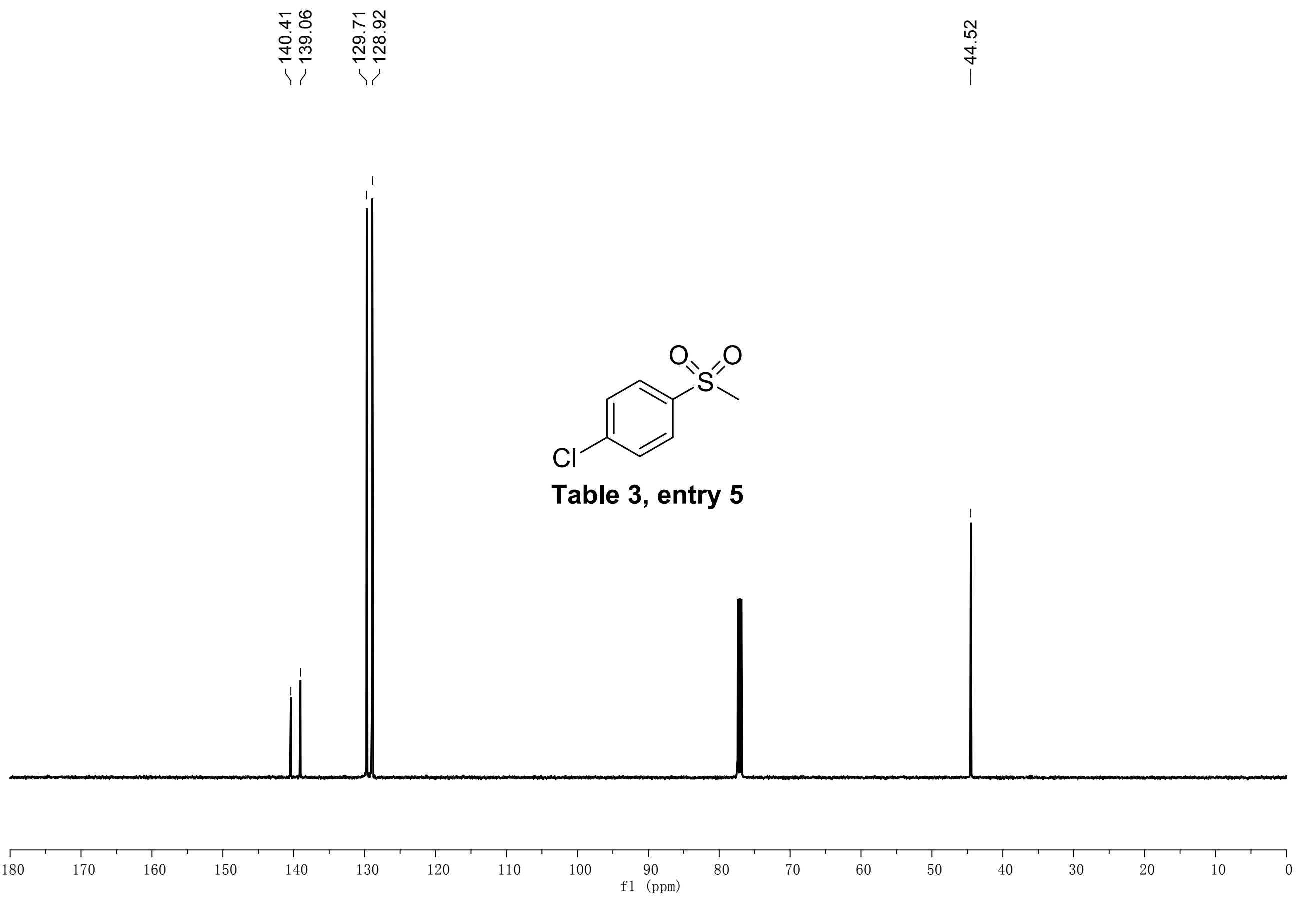

^{1}H NMR and ^{13}C NMR of **3f.**

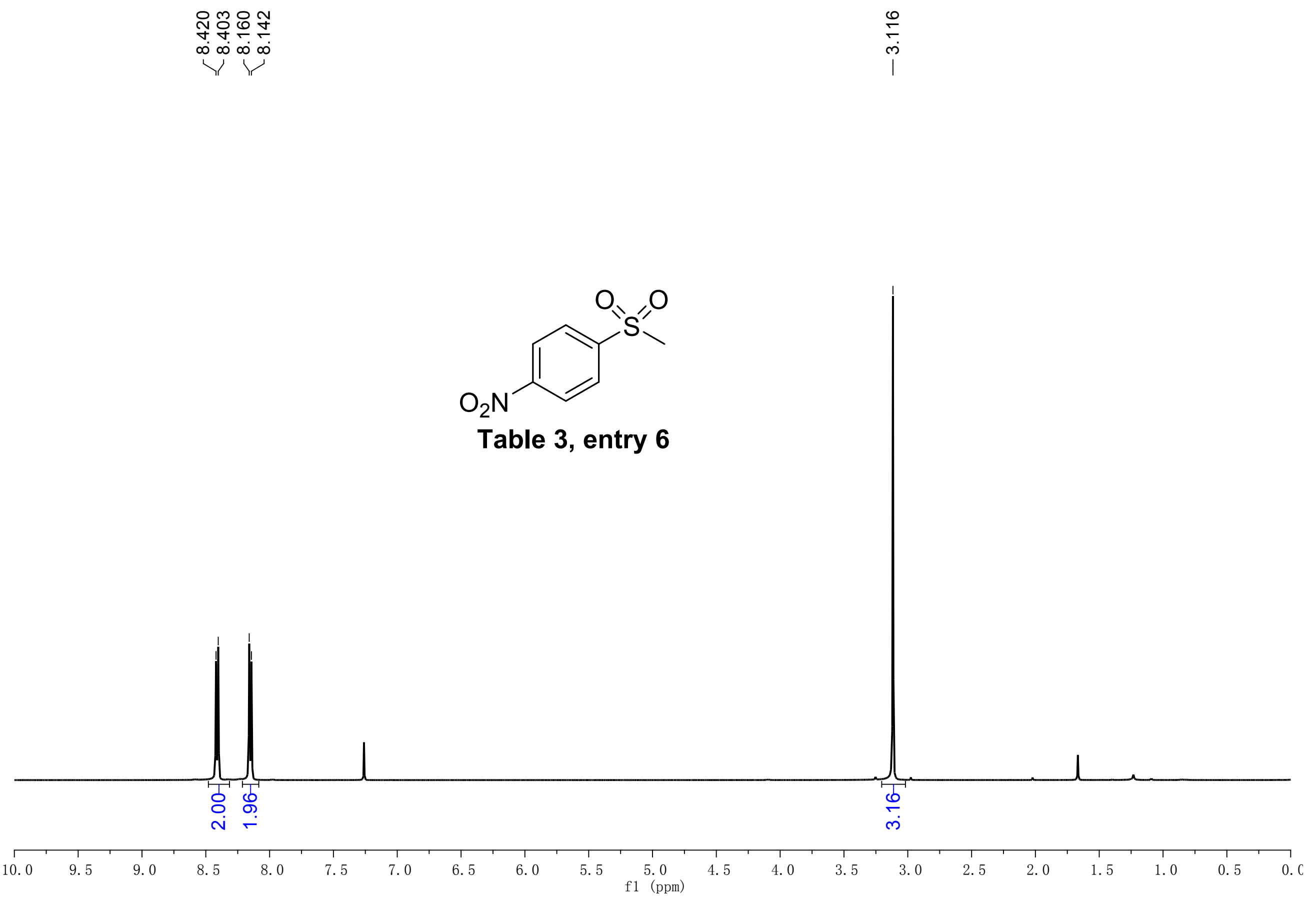
8.420
8.403
8.160
8.142
3.116
O
S
O
O_2N
Table 3, entry 6
2.00
1.96
3.16
10.0 9.5 9.0 8.5 8.0 7.5 7.0 6.5 6.0 5.5 5.0 4.5 4.0 3.5 3.0 2.5 2.0 1.5 1.0 0.5 0.0
f1 (ppm)

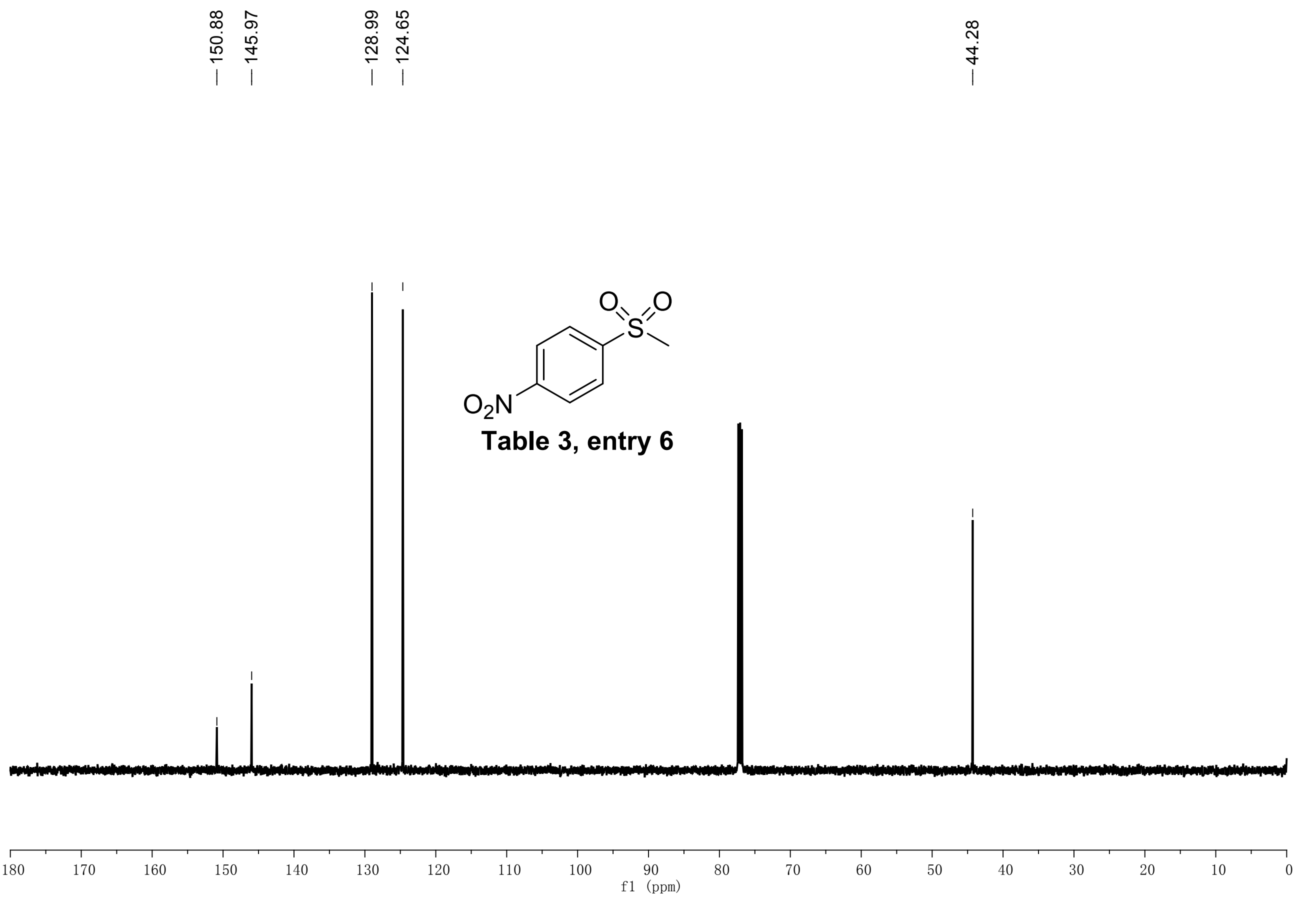

^{1}H NMR and ^{13}C NMR of **3g.**

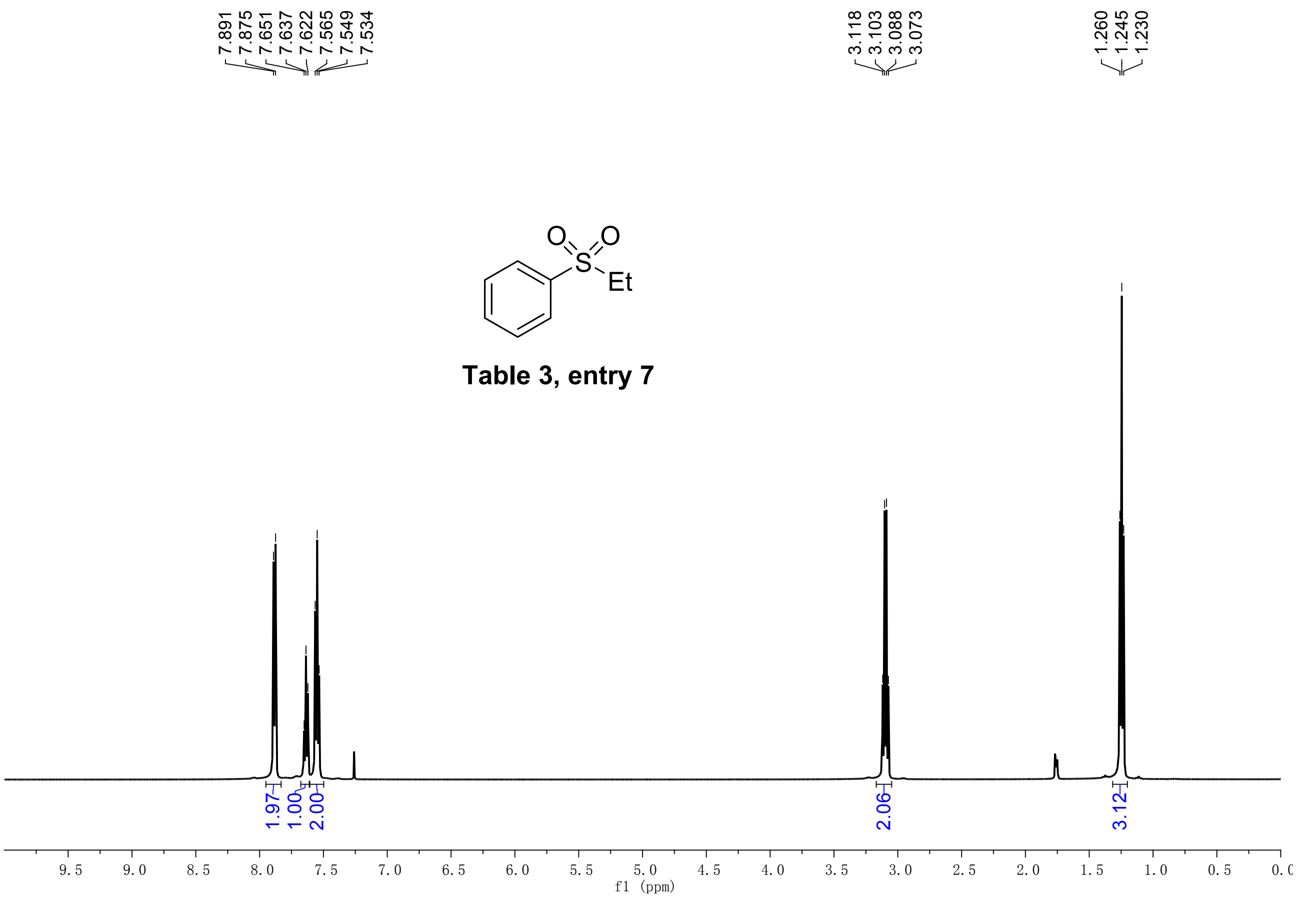
7.891
7.875
7.651
7.637
7.622
7.565
7.549
7.534
3.118
3.103
3.088
3.073
1.260
1.245
1.230
O
O
S
Et
Table 3, entry 7
1.97
1.00
2.00
2.06
3.12
9.5 9.0 8.5 8.0 7.5 7.0 6.5 6.0 5.5 5.0 4.5 4.0 3.5 3.0 2.5 2.0 1.5 1.0 0.5 0.0
f1 (ppm)

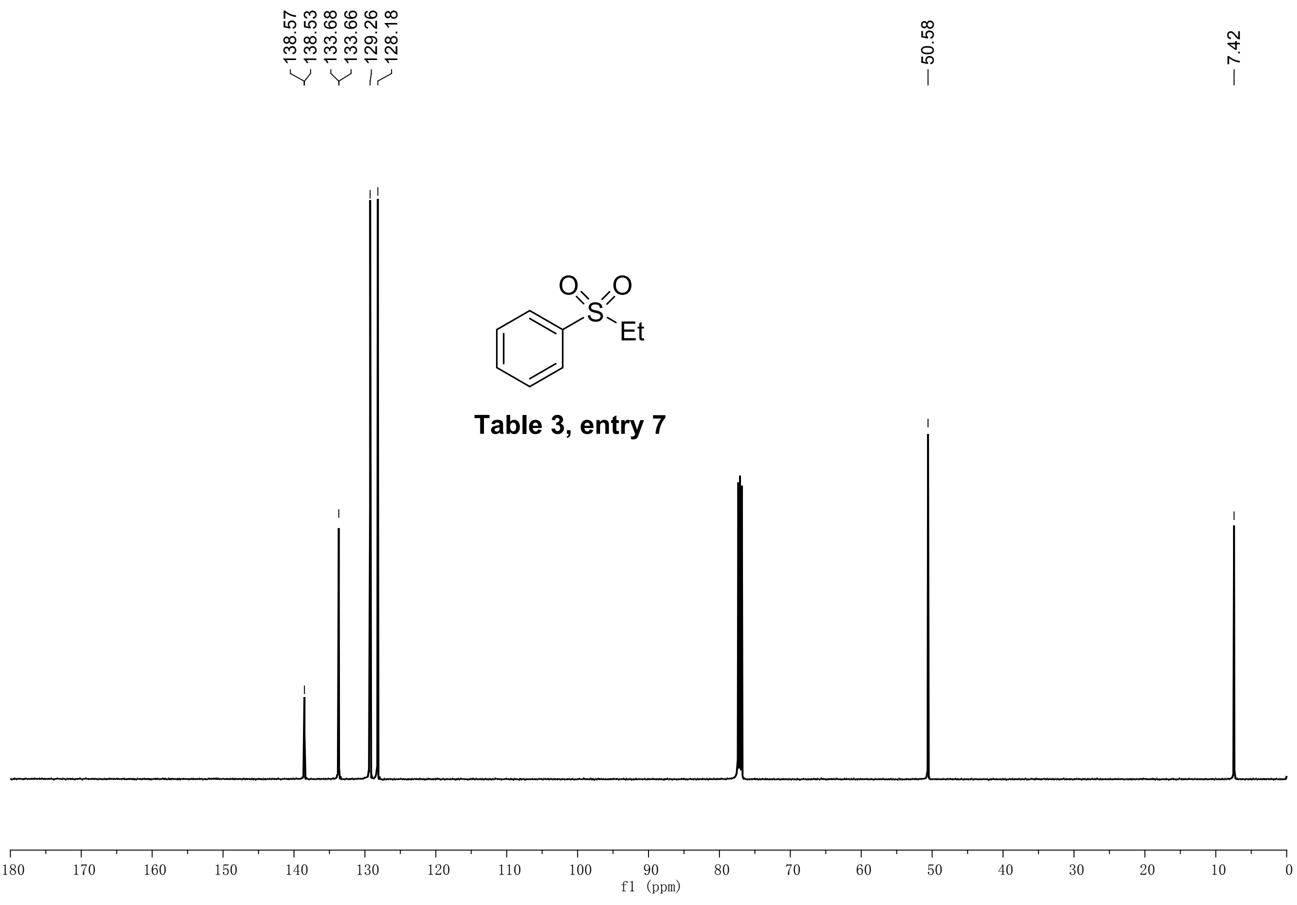

^{1}H NMR and ^{13}C NMR of **3h.**

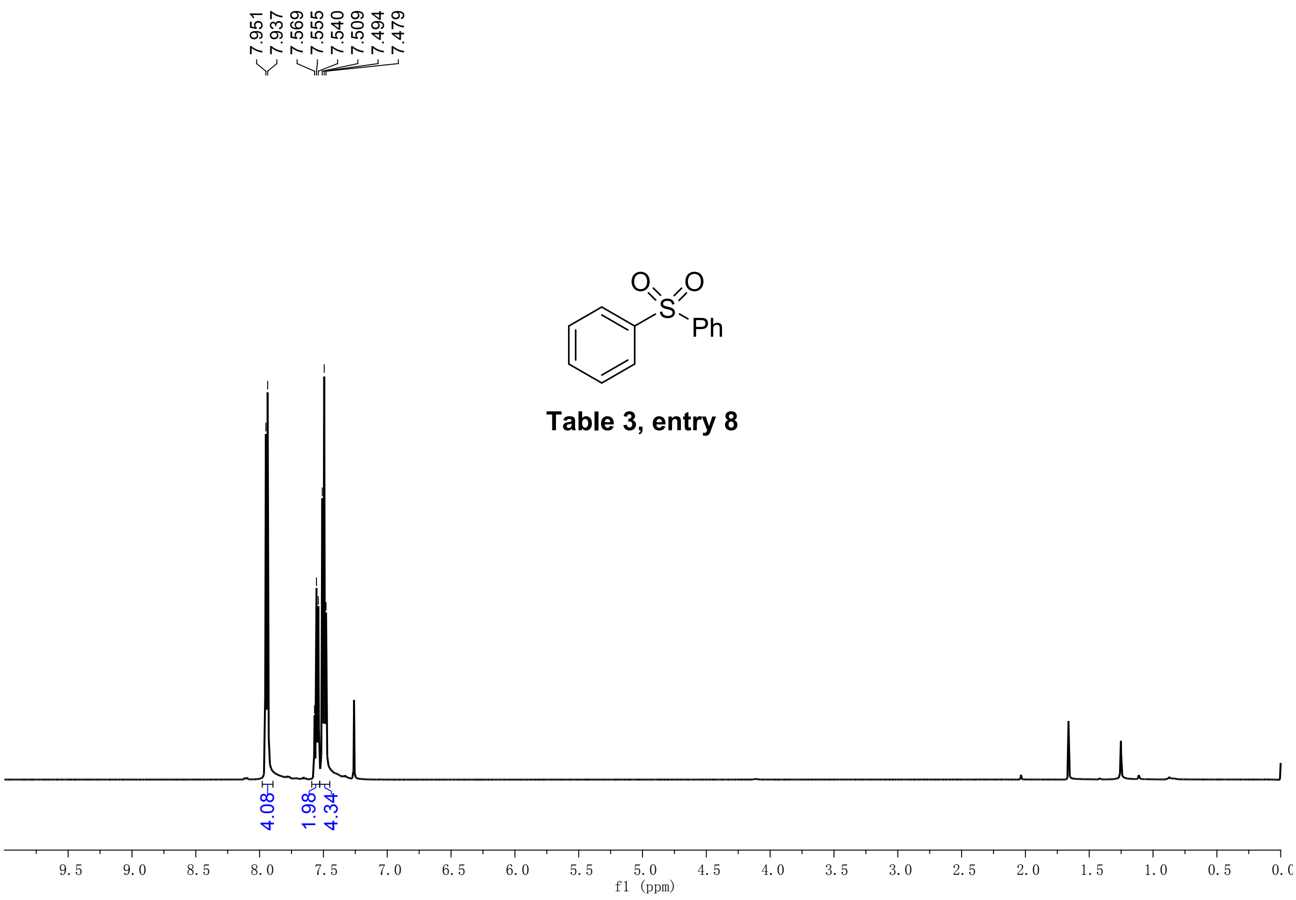

Table 3, entry 8

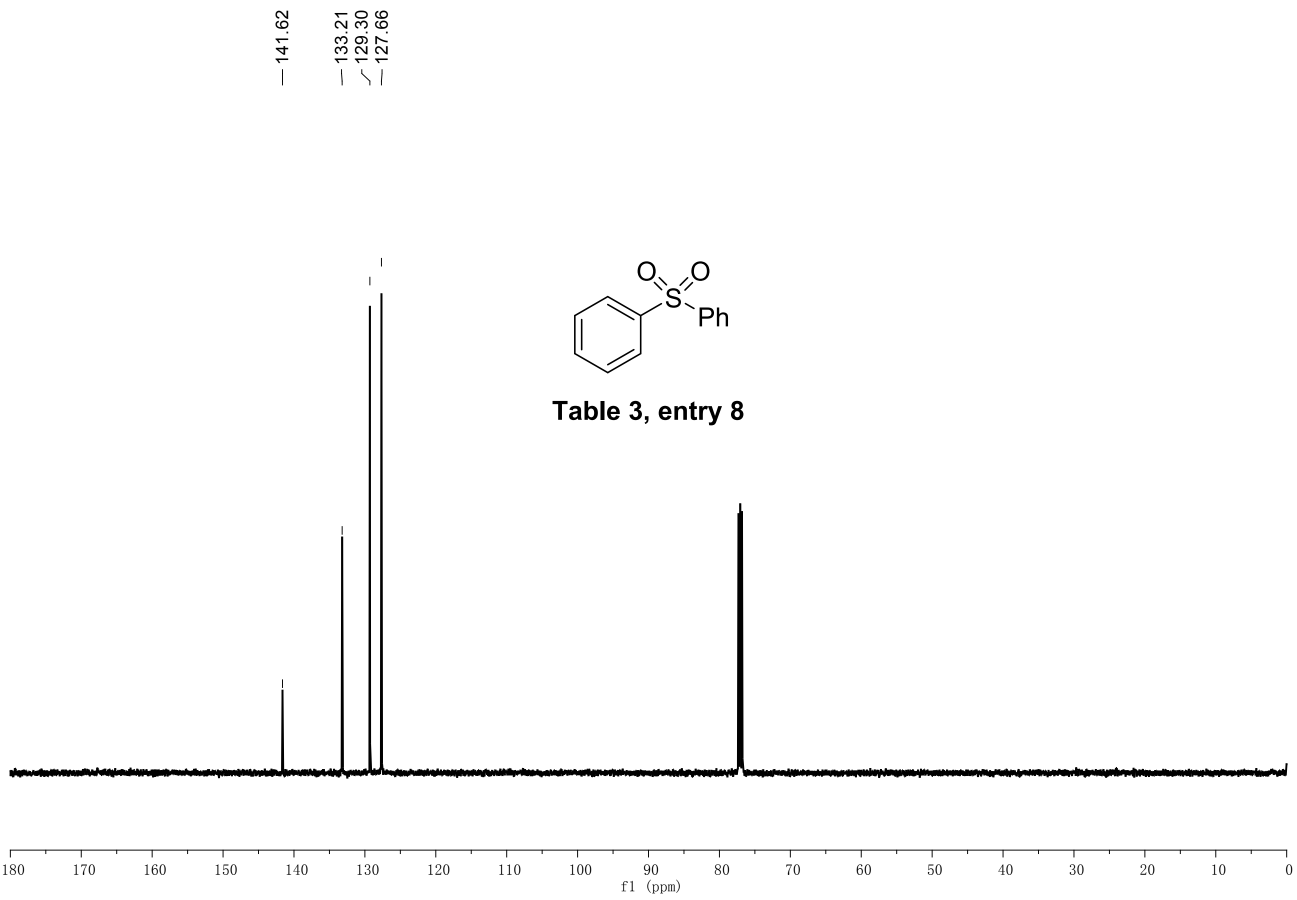

^{1}H NMR and ^{13}C NMR of **3i.**

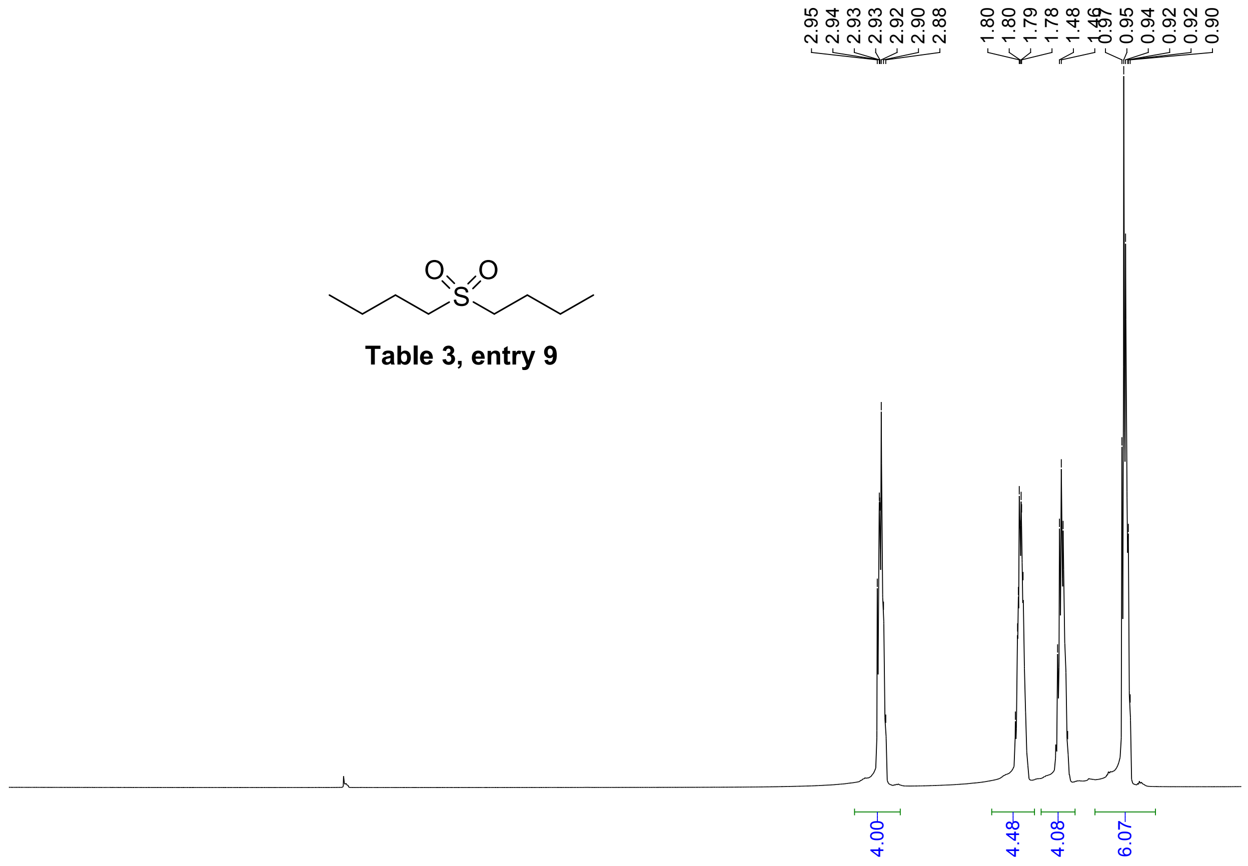

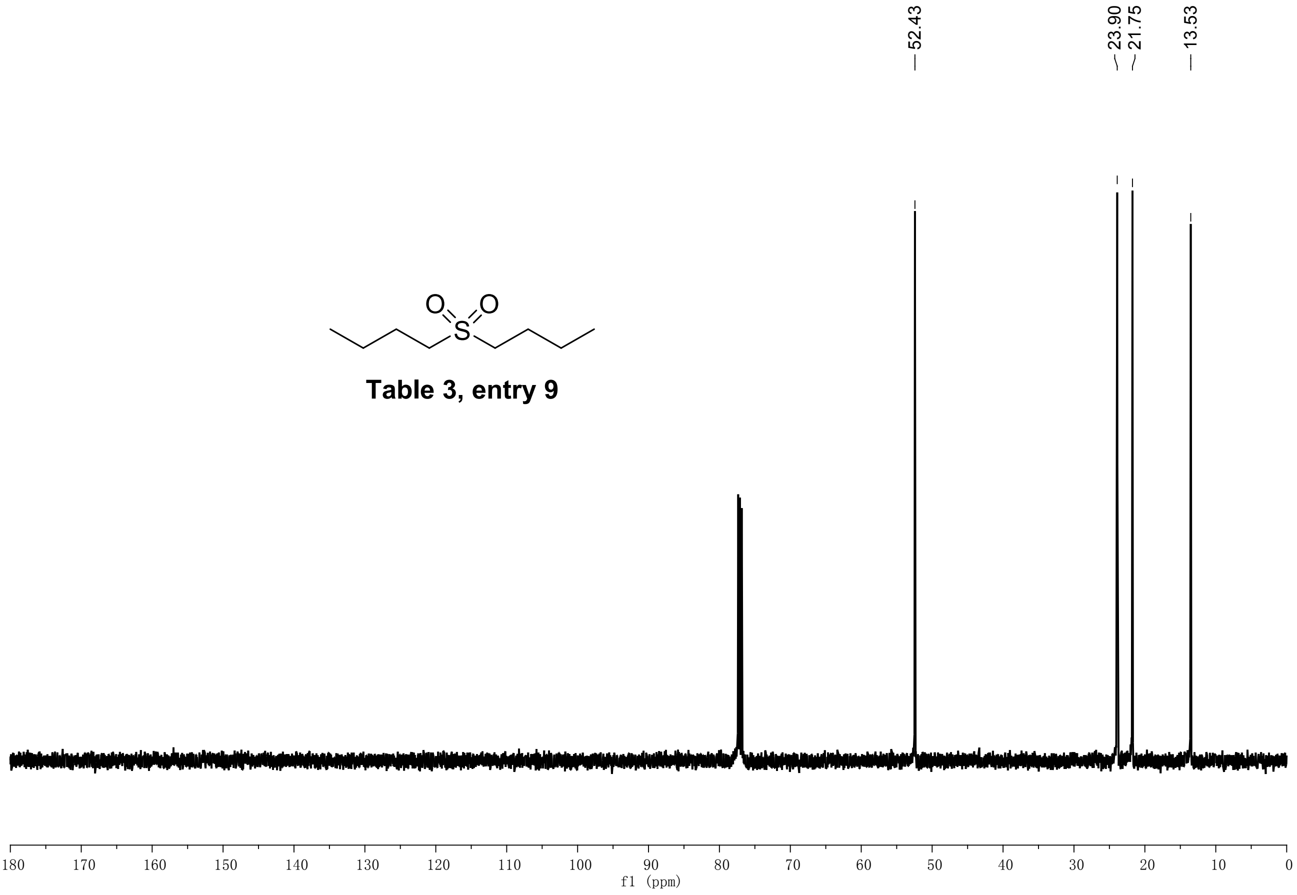
52.43
23.90
21.75
13.53
Table 3, entry 9
180 170 160 150 140 130 120 110 100 90 80 70 60 50 40 30 20 10 0
f1 (ppm)
